# Phage portal proteins counteract stringent-response–mediated restriction

**DOI:** 10.1038/s41467-026-75532-5

**Published:** 2026-07-11

**Authors:** Kristina Kronborg, Luokai Wang, Muriel Leandra Schicketanz, Kenn Gerdes, Yong Everett Zhang

**Affiliations:** 1 Department of Biology, University of Copenhagen, Copenhagen, Denmark; 2 School of Clinical and Basic Medical Sciences, Shandong First Medical University & Shandong Academy of Medical Sciences, Jinan, China; 3 Independent researcher, Copenhagen, Denmark; 4 Present Address: Novonesis A/S, Kgs, Lyngby, Denmark

**Keywords:** Bacteriophages, Bacteriology, Bacterial physiology, Phage biology, Viral immune evasion

## Abstract

Bacteria restrict viral replication not only through dedicated defense systems but also by entering physiological states that limit cellular resources, yet how phages overcome such host-imposed barriers remains unclear. The stringent response, driven by the alarmone nucleotides ppGpp and pppGpp, can impose a growth-restrictive state that hinders phage infection in specific phage–host contexts. Here, we show that alarmone signaling constrains bacteriophage T7 infection and that the portal protein Gp8 counteracts this barrier by engaging RelA and SpoT, inhibiting their synthetase activities and suppressing alarmone accumulation. Portal mutations that disrupt this interaction sustain alarmone elevation, delay lysis and impair replication in a manner relieved in alarmone-deficient hosts. Portal proteins from representative coliphages share related stringent-response-linked features, indicating that essential virion components can moonlight as antagonists of host stress physiology and that this mechanism is not unique to T7 and may extend to additional coliphages.

## Abstract (web summary)

Bacteria can protect themselves against viral infections by entering physiological states, such as the stringent response, that limit cellular resources. Here, the authors show that phages can use their portal proteins to suppress the bacterial stringent response, thus promoting infection.

## Introduction

Bacteria employ multilayered defense networks to restrict viral infection, combining dedicated systems such as restriction-modification, CRISPR-Cas, abortive infection modules, and nucleotide-based immune signaling pathways with broader physiological states that limit viral replication^1–4^. Whereas many antiviral mechanisms rely on molecular recognition of invading phages, global physiological responses can impose resource constraints that indirectly restrict infection. Physiological growth arrest, a common outcome of abortive infection systems, limits phage spread by sacrificing infected cells^5^. How phages contend with such host-imposed physiological barriers remains incompletely understood.

One of the most ancient and conserved physiological responses is the stringent response (SR), mediated by the alarmones ppGpp and pppGpp^6,7^. Activation of the SR reprograms transcription and metabolism, suppressing ribosome biogenesis and nucleotide synthesis while promoting stress adaptation^8^. Although classically viewed as a stress-adaptation mechanism, the growth-restrictive state imposed by the SR creates an intracellular environment that is unfavorable for bacteriophage replication, suggesting that it can function as a physiological barrier to infection.

Consistent with this view, (p)ppGpp signaling influences bacteriophage infection outcomes. Cells lacking alarmone synthesis are hypersensitive to phage killing, whereas stationary-phase cells, characterized by elevated alarmone levels, often exhibit increased resistance^9–11^. Toxic Rel-family proteins have also emerged in multiple abortive infection systems^12–15^, underscoring the recurrent involvement of RelA-SpoT Homologs (RSH)-associated regulatory pathways in phage–host interactions. Conversely, certain phage-encoded factors, such as the alarmone-degrading enzyme ATD1^16^, directly modulate (p)ppGpp metabolism during infection. Together, these observations indicate that alarmone-linked stress networks are repeatedly engaged during defense and counter-defense processes.

Phages, in turn, have evolved diverse strategies to neutralize host defenses, including inhibitors of CRISPR-Cas systems, restriction enzymes, abortive infection pathways, and cyclic-nucleotide immune signaling modules^17,18^. Most characterized anti-defense factors target discrete molecular effectors. Whether phages also encode dedicated mechanisms to counteract global physiological restriction states, such as the SR, remains largely unexplored. Because the SR is governed by a small number of conserved enzymes, it represents a potentially vulnerable node for viral counter-defense.

In *Escherichia coli*, the SR is mediated primarily by the (p)ppGpp synthetases RelA and SpoT^19–23^. Elevated alarmone levels remodel transcription through direct interactions with RNA polymerase (RNAP)^24,25^, limiting biosynthetic capacity and slowing growth^26–28^. Although this reprogramming enhances stress survival, it may also constrain the metabolic demands of phage replication. Consistent with this, bacteriophage T7 encodes multiple inhibitors of host RNAP^11,29–31^, highlighting the importance of transcriptional control during infection. These observations raise the possibility that phages actively modulate SR signaling to optimize infection.

Here, we investigate the interplay between the SR and infection by bacteriophage T7. Using complementary genetic, biochemical, and phage mutational approaches, we identify the essential T7 capsid portal protein Gp8 as a direct inhibitor of (p)ppGpp synthesis. Gp8 physically interacts with RelA and SpoT, selectively suppressing their synthetase activities and reducing intracellular alarmone accumulation during infection. Disruption of this interaction impairs phage replication and delays host lysis. Portal proteins from diverse *E. coli* phages exhibit similar properties, suggesting that this strategy may extend beyond T7, by which structural virion components moonlight as modulators of host stress physiology to promote infection.

## Results

### The stringent response limits bacteriophage infection

**Fig. 1:**
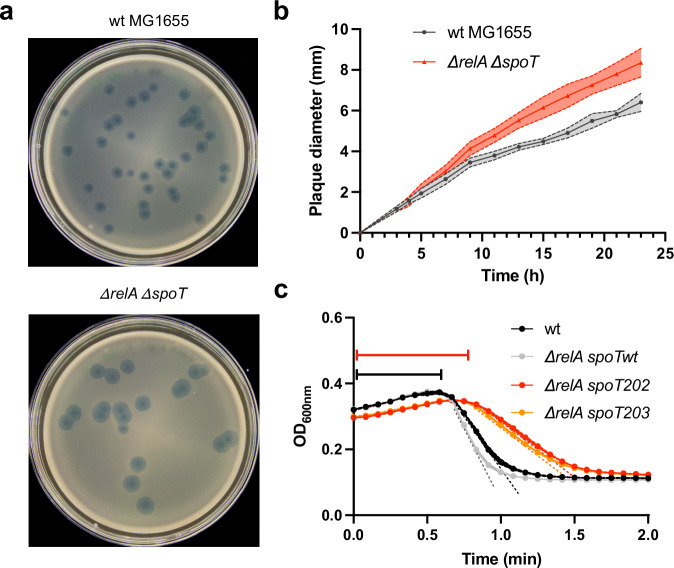
The stringent response restricts bacteriophage infection. **a** Plaques of T7 phage after 24 h infection of wild-type and (p)ppGpp⁰ (Δ*relA* Δ*spoT*) *Escherichia coli* on LB medium. Representative plates from three independent biological replicates are shown. **b** Quantification of plaque expansion kinetics for T7 infection of wild-type and (p)ppGpp⁰ (Δ*relA* Δ*spoT*) cells, as in (**a**). Data represent mean ± s.d (shaded areas) from five independent plaques per strain. **c** Lysis kinetics of wild-type MG1655, Δ*relA spoT*^wt^ and high-(p)ppGpp strains (Δ*relA spoT202* and Δ*relA spoT203*) during T7 infection. Data represent mean values from three independent biological experiments with s.d. indicated by shaded areas. The lag phase preceding lysis and the lysis rate (slope) are indicated. Source data are provided as a Source Data file.

Bacterial stress responses reshape cellular physiology in ways that can influence susceptibility to viral infection. To test whether the stringent response (SR) affects bacteriophage replication, we examined infection by bacteriophage T7 in a (p)ppGpp⁰ *Escherichia coli* strain lacking both *relA* and *spoT*. Compared with wild-type cells, the alarmone-deficient host exhibited accelerated killing kinetics and more rapid lysis, as reflected by larger plaque sizes and increased plaque expansion rates (Fig. 1a, b). These results indicate that loss of alarmone signaling enhances phage infection, consistent with a previous report^11^.

We next tested whether elevated alarmone levels have the opposite effect. Strains carrying *spoT* alleles that increase basal (p)ppGpp concentrations (*spoT202* and *spoT203*)^32,33^ displayed delayed lysis kinetics relative to *spoT* wild-type allele cells, characterized by prolonged lag phases prior to host population collapse and reduced lysis slopes (Fig. 1c). In the strains tested, particularly within the Δ*relA spoT* allelic series, higher intracellular alarmone levels were associated with slower infection progression.

Together, these results demonstrate that alarmone signaling establishes a growth-restrictive intracellular environment that limits bacteriophage replication in the T7-*E. coli* system examined here. The SR can therefore function as a physiological barrier to infection, suggesting that T7 must encode mechanisms to overcome this defense-associated state.

### T7 encodes factors that interfere with stringent-response-dependent host physiology

Because elevated alarmone levels restrict phage infection (Fig. 1), we reasoned that T7 may encode factors that counteract the SR**-**associated physiological state. Anti-defense systems often manifest as proteins that perturb host pathways required specifically under stress or nutrient-limited conditions. We therefore performed a systematic functional screen to identify T7 proteins that interfere with host growth under conditions in which (p)ppGpp signaling is required.

**Fig. 2:**
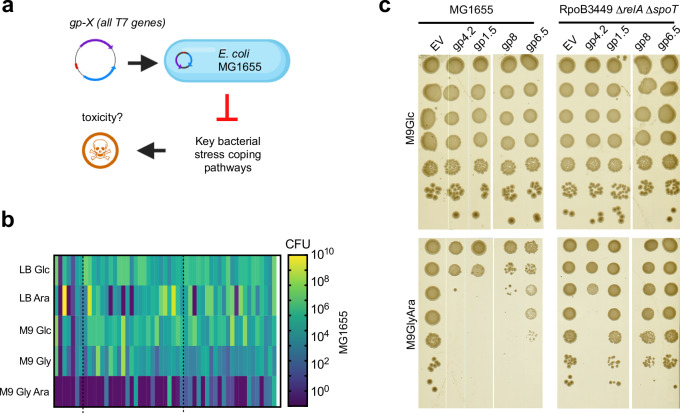
Identification of T7 proteins linked to stringent-response-dependent physiology. **a** Schematic of the systematic testing of all 53 phage T7 genes into the inducible plasmid vector pBAD33 and their expression in *Escherichia coli* MG1655 to screen for phage proteins that impair bacterial growth under conditions requiring stringent-response function. (Created in BioRender. ZHANG, Y. (2026) https://BioRender.com/ rud90dy. **b** Growth of *E. coli* expressing individual T7 genes in rich and minimal media, quantified as CFU ml⁻¹ normalized to OD_600nm_. LB Glc and LB Ara are LB media supplemented with 0.2% glucose and 0.2% arabinose, respectively. M9 Glc, M9 Gly, and M9 Gly Ara denote M9 minimal media supplemented with 0.4% glucose, 0.4% glycerol, or 0.4% glycerol plus 0.2% arabinose, respectively. Original plate-based growth assays are shown in Supplementary Fig. 1. **c** Suppression of T7 protein**-**induced growth defects in (p)ppGpp⁰ (*ΔrelA ΔspoT*) strains carrying stringent-like RNA polymerase allele (*rpoB*3449; RNAP^SR^). Serial dilutions of the indicated strains expressing *gp1.5*, *gp4.2*, gp6.5, *gp8*, or empty vector are shown. Source data are provided as a Source Data file.

We cloned 53 T7 genes under the arabinose inducible control of the pBAD33 vector and assessed their effects on *E. coli* growth in rich (LB) and minimal media (M9) (Fig. 2a). Strikingly, 34 of 53 T7 proteins caused severe growth inhibition when expressed in minimal medium, whereas most exhibited little or no toxicity in rich medium (Fig. 2b and Supplementary Fig. 1). This conditional toxicity pattern suggests that T7 encodes numerous factors targeting host processes that become critical under nutrient limitation, a physiological context strongly influenced by alarmone signaling.

To identify T7 proteins specifically linked to SR-dependent physiology, we next exploited RNA polymerase (RNAP) mutants with a stringent-like (RNAP^SR^) phenotype that bypass the requirement for (p)ppGpp during growth in minimal medium^34,35^. For four T7 proteins, Gp1.5, Gp4.2, Gp6.5, and Gp8, the growth defect observed in wild-type cells was reproducibly alleviated in two independent RNAP^SR^ backgrounds (Supplementary Fig. 2). Notably, suppression persisted in a (p)ppGpp⁰ strain carrying the RNAP^SR^ allele^34^ (Fig. 2c), indicating that rescue does not require alarmone synthesis itself but rather depends on SR-regulated transcriptional programs.

Collectively, these results identify a subset of T7 proteins genetically linked to host SR pathways, consistent with the presence of viral factors that interfere with this defense-associated physiological state.

### The portal protein Gp8 directly targets the alarmone synthetases RelA and SpoT

**Fig. 3:**
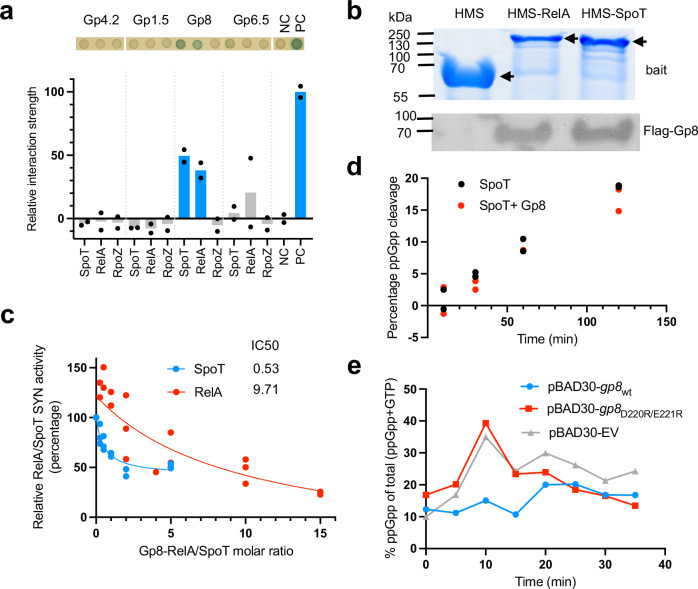
The portal protein Gp8 inhibits the alarmone synthetases RelA and SpoT. **a** Bacterial two-hybrid assays testing interactions between T7 proteins and RelA, SpoT, or RpoZ (the ω subunit of RNA polymerase). Representative spots are shown above the corresponding bars. Bar plots show quantification of relative interaction strengths, normalized to the positive control (PC). Data represent the mean of *n* = 2 independent biological experiments. NC and PC denote negative and positive controls, respectively. **b** Pull-down assays showing co-purification of Flag**-**Gp8 with His**-**MBP**-**SUMO (HMS)**-**tagged RelA or SpoT, or with the HMS tag alone. Arrows indicate bands corresponding to the bait proteins. Uncropped blots are provided in Source Data. Images representative of two independent experiments. **c** In vitro (p)ppGpp synthetase activity of RelA and SpoT in the presence of increasing amounts of purified Gp8. Gp8 concentrations are plotted as molar ratios to RelA or SpoT using Gp8 monomer equivalents. The inhibitory plateau is not interpreted as a binding stoichiometry. Data were fitted in GraphPad Prism using the inhibitor vs. response model, three parameters. Corresponding IC50 molarity values are indicated in the panel. **d** SpoT hydrolase activity measured in the absence or presence of Gp8 in two independent biological experiments. **e** Intracellular ppGpp levels, normalized to total (ppGpp + GTP), following Gp8 expression from the pBAD30 vector, measured by thin-layer chromatography. EV, empty vector control. The representative thin-layer chromatograph of two independent biological replicates is shown in Supplementary Fig. 3b. Source data are provided as a Source Data file.

The genetic link between a subset of T7 proteins and SR**-**dependent host physiology prompted us to test whether these candidates directly target components of the alarmone signaling machinery. We therefore examined physical interactions between Gp1.5, Gp4.2, Gp6.5, and Gp8 and the central SR regulators RelA, SpoT, and RNAP (i.e., the ω subunit RpoZ) using a bacterial two-hybrid assay. Among the proteins tested, the T7 portal protein Gp8 showed robust and reproducible interactions with both RelA and SpoT (Fig. 3a), whereas the other candidates did not display consistent interactions.

We validated these associations biochemically by co-purification assays using Flag-tagged Gp8 and His**-**MBP**-**SUMO(HMS)-tagged RelA or SpoT. In both cases, Gp8 specifically co-purified with the alarmone synthetases, supporting direct physical engagement in vitro (Fig. 3b). These results identify Gp8, an essential structural component of the T7 capsid portal vertex, as a viral protein that engages the core enzymatic machinery responsible for (p)ppGpp synthesis.

We next asked whether Gp8 binding affects the catalytic activities of RelA and SpoT. In vitro enzymatic assays revealed that purified Gp8 inhibited the synthetase activities of both enzymes in a dose-dependent manner (Fig. 3c and Supplementary Fig. 3a). We note that the RelA assay used here represents a simplified biochemical setting and was not intended to fully reconstitute the ribosome-associated activation state of RelA in vivo. Rather, these experiments establish that Gp8 is sufficient to suppress RelA and SpoT synthetase activity in the absence of additional cellular components. In contrast, Gp8 had no detectable effect on the hydrolase activity of SpoT (Fig. 3d), indicating that the portal protein selectively suppresses alarmone synthesis rather than globally perturbing SpoT function. Consistent with this, expression of Gp8 in vivo reduced intracellular (p)ppGpp accumulation relative to control conditions (Fig. 3e and Supplementary Fig. 3b).

The combined genetic and biochemical evidence support the conclusion that the phage portal protein Gp8 directly engages RelA and SpoT and inhibits their alarmone synthetase activities, revealing a mechanism by which T7 suppresses the SR signaling pathway.

### Electrostatic surfaces on the portal ring mediate targeting of RelA and SpoT

**Fig. 4:**
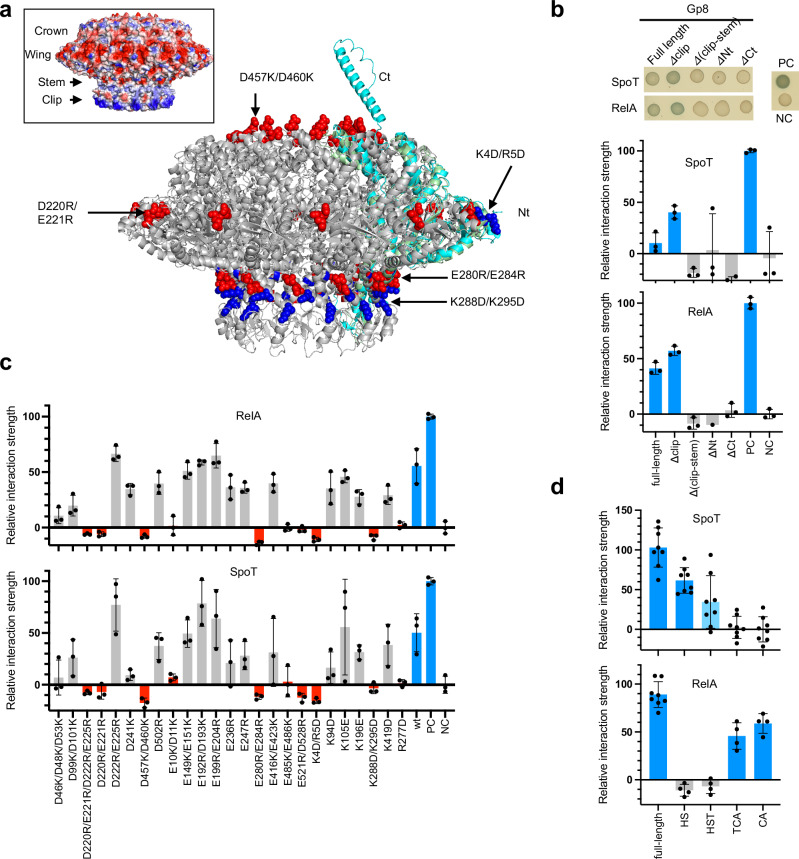
Charged surfaces of Gp8 portal ring contribute to interactions with RelA and SpoT. **a** Structure of the T7 Gp8 portal ring (gray ribbon model; PDB 7EY6). An AlphaFold model of the full-length Gp8 monomer (cyan) is overlaid with one monomer from the portal ring structure (green) to indicate the positions of the N- and C-terminal regions, including the K4 and R5 residues. Residues whose mutation abolished interaction with RelA and SpoT are shown as spheres and labeled. Negatively and positively charged residues are shown in red and blue, respectively. Inset: electrostatic surface representation of the Gp8 portal ring. **b** Bacterial two-hybrid assays assessing interactions between Gp8 truncation variants and full-length RelA or SpoT. Δclip, deletion of residues 299**–**330; Δ(clip**-**stem), deletion of residues 269**–**355; ΔNt, deletion of residues 6**–**42; ΔCt, deletion of residues 484**–**543. Bar plots show relative interaction strengths normalized to the positive control. NC and PC denote negative and positive controls, respectively. NC corresponds to cells carrying empty vectors. Data are mean ± s.d., *n* = 3 independent biological experiments. **c** Bacterial two-hybrid assays testing interactions between Gp8 point mutants and full-length RelA or SpoT. Bar plots show relative interaction strengths normalized to the positive control. WT denotes wild-type Gp8. NC denotes cells carrying empty vectors. Data are mean ± s.d., *n* = 3 independent biological experiments. Red bars indicate Gp8 mutants that lost interaction with both RelA and SpoT. Representative raw spot images are shown in Supplementary Fig. 4a–e. **d** Bacterial two-hybrid assays testing interactions between Gp8 and truncated variants of RelA or SpoT. Interaction strengths are normalized to the respective full-length RelA or SpoT proteins. Data are mean ± s.d., *n* = 4 or 8 independent biological experiments. Domain abbreviations: HST, hydrolase**-**synthetase**-**TGS domains; HS, hydrolase**-**synthetase domains; TCA, TGS**-**CC**-**ACT domains; CA, CC**-**ACT domains. Precise domain boundaries of the RelA and SpoT truncations, along with representative raw spot images, are provided in Supplementary Fig. 4f, g. Source data are provided as a Source Data file.

Gp8 assembles into a dodecameric ring that forms the portal vertex of the T7 capsid, with exterior surfaces enriched in charged residues (Fig. 4a). To define the regions of Gp8 required for interaction with RelA and SpoT, we generated domain truncations and assessed binding using the bacterial two-hybrid assay. Deletion of either the N- or C-terminal regions of Gp8 abolished interactions with both RelA and SpoT, whereas removal of the clip domain reproducibly enhanced interaction signals. Further deletion extending into the stem domain eliminated binding entirely (Fig. 4b). These results indicate that an intact portal ring architecture is required for targeting the alarmone synthetases and suggest that the clip motif does not itself contribute to binding but instead modulates access to the interaction surface.

Notably, the clip motif is enriched in positively charged residues, whereas the remainder of the portal exterior is predominantly electronegative (Fig. 4a). The observation that deletion of a positively charged element enhances binding suggests that RelA and SpoT preferentially engage negatively charged surfaces on the assembled portal ring and that the clip motif may partially shield or sterically constrain these interfaces in the intact protein. We therefore tested whether electrostatic interactions underlie RelA/SpoT binding by generating 43 charge-reversal mutants targeting clusters of surface-exposed acidic and basic residues.

Approximately half of the charge-reversal mutants exhibited substantial loss of interaction with RelA, and the same substitutions similarly impaired SpoT binding (Fig. 4c and Supplementary Fig. 4a–e). Several mutations, including D220R/E221R, D457K/D460K, E280R/E284R, K4D/R5D and K288D/K295D, abolished detectable interaction with both RelA and SpoT in the bacterial two-hybrid assay, indicating that these charged regions are important for the Gp8-dependent interaction readout (Fig. 4a). Because some of these charge-reversal mutations lie within the stem/clip region, altered local electrostatics may also affect portal conformation or oligomer stability, potentially explaining the lack of viable mutant phage recovery described below.

To define the corresponding interfaces on the host proteins, we performed domain mapping of RelA and SpoT. Removal of the N-terminal catalytic domains of SpoT eliminated its interaction with Gp8, whereas truncation of the C-terminal regulatory domains of RelA disrupted binding (Fig. 4d and Supplementary Fig. 4f, g). Thus, Gp8 engages RelA and SpoT through distinct domain interfaces, while relying on a common set of electrostatically defined surfaces on the portal ring.

In combination, these data indicate that the portal protein uses a spatially organized pattern of surface charges to engage the alarmone synthesis machinery, consistent with a specific interaction interface on Gp8.

### Suppression of alarmone signaling by Gp8 is important for efficient phage infection

**Fig. 5:**
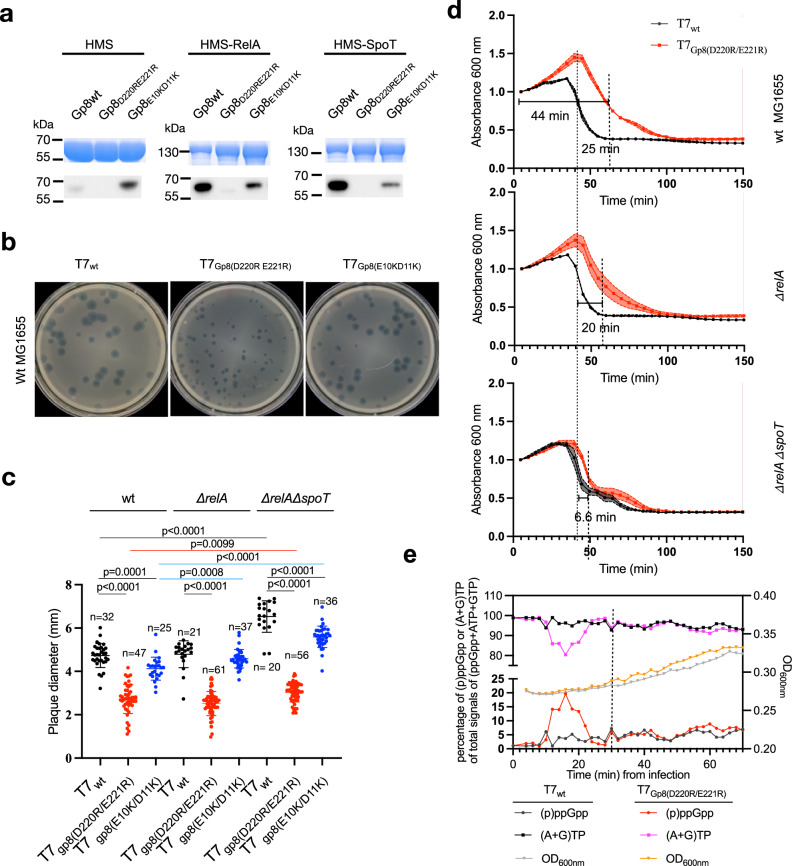
Portal-mediated suppression of alarmone signaling is required for efficient infection. **a** Pull-down assays using HMS-tag, HMS-RelA, or HMS-SpoT as bait with Flag-tagged wild-type (WT) or mutant Gp8 proteins. Top: SDS-PAGE analysis of the bait proteins; bottom: immunoblot detection of co-purified Flag-Gp8 using an anti-Flag antibody. Images representative of two independent experiments. **b** Representative plaque morphologies of WT or mutant T7 phages infecting WT *E. coli* MG1655 cells, imaged 18 h post infection. Representative plates from three independent biological replicates are shown. **c** Quantification of plaque sizes corresponding to panel (**b**) for WT and mutant T7 phages infecting WT, Δ*relA*, or (p)ppGpp⁰ (Δ*relA* Δ*spoT*) strains. Data are shown as scatter plots with mean ± s.d. The numbers of quantified plaques are indicated above each plot. Statistical significance was assessed using an unpaired two-tailed Student’s *t* test; exact *P-*values are indicated. **d** Liquid infection assays of WT or mutant T7 phages infecting WT, Δ*relA*, or (p)ppGpp⁰ (Δ*relA* Δ*spoT*) strains. Dashed lines indicate the time points at which 50% of the total OD_600nm_ decrease associated with lysis had occurred; corresponding durations and differences are indicated. Lines and shaded areas indicate mean ± s.d. **e** Quantification of ATP, GTP, and (p)ppGpp levels in radiolabeled *E. coli* MG1655 cells following infection with WT or mutant T7 phage. OD_600nm_ values of the host cultures are plotted on the secondary (right) *y*-axis. Experiments were performed in MOPS medium supplemented with 0.2 mM K₂HPO₄ and 0.2% (w/v) glucose. Representative thin-layer chromatography plates are shown in Supplementary Fig. 5b, c. The first cycle of cell lysis (indicated by a dashed line) occurred approximately 30 min post infection. Source data are provided as a Source Data file.

To determine whether Gp8-mediated targeting of RelA and SpoT contributes to phage fitness, we generated T7 phages carrying portal mutations that disrupt interaction with the alarmone synthetases. Using recombineering, we isolated mutant phages bearing substitutions D220R/E221R (interaction-defective) and E10K/D11K (partially defective), and confirmed impaired binding to RelA and SpoT by pull-down assays (Fig. 5a).

In plaque assays on wild-type *E. coli*, the interaction-defective mutant formed significantly smaller plaques than T7_wt_, whereas the partially defective mutant displayed an intermediate phenotype (Fig. 5b, c). These defects were alleviated in hosts lacking SR signaling: plaque sizes increased in Δ*relA* cells for the partially defective mutant and in (p)ppGpp⁰ cells for all phages tested, indicating that impaired infection is linked to the host alarmone pathway.

We next quantified infection dynamics in liquid culture at a multiplicity of infection (MOI) of 0.1. Compared with T7_wt_, the interaction-defective mutant exhibited a marked delay in host cell lysis during infection of wild-type cells (Fig. 5d). This delay was substantially reduced in Δ*relA* and nearly abolished in (p)ppGpp⁰ strains, demonstrating that elevated alarmone levels are responsible for much of the infection defect (Fig. 5d). This is consistent with the prior observation that hosts with genetically elevated basal (p)ppGpp levels displayed delayed lysis (Fig. 1c), supporting an inverse relationship between alarmone abundance and infection efficiency.

To directly monitor alarmone dynamics during infection, we radiolabeled intracellular nucleotide pools and quantified (p)ppGpp levels over time. Because complex media such as LB interfere with nucleotide labeling, these experiments were performed in defined MOPS medium. Importantly, the relative infection phenotypes observed in LB were preserved under these conditions, with wild-type T7 lysing *E. coli* more rapidly than the interaction-defective mutant (Supplementary Fig. 5a).

Infections were performed at the same MOI of 0.1, such that population-wide lysis requires at least two rounds of infection, and alarmone dynamics are most clearly resolved during the first, relatively synchronized infection cycle. During this initial cycle, infection with the interaction-defective T7 mutant resulted in sustained elevation of (p)ppGpp prior to the onset of lysis, whereas alarmone levels were markedly reduced during wild-type T7 infection (Fig. 5e and Supplementary Fig. 5b, c). Elevated (p)ppGpp levels coincided with pronounced depletion of the GTP pool, consistent with SR-associated repression of nucleotide biosynthesis^36–39^. At later time points corresponding to subsequent infection cycles and final population collapse, differences in alarmone levels between wild-type and mutant infections were diminished, likely reflecting increased heterogeneity in infection timing following the first lysis event.

Taken together, these results indicate that portal-mediated suppression of alarmone signaling promotes efficient T7 infection and that disruption of the Gp8-RelA/SpoT interaction leads to sustained SR activation and delayed infection progression.

### Portal-mediated targeting of the stringent response extends beyond T7

**Fig. 6:**
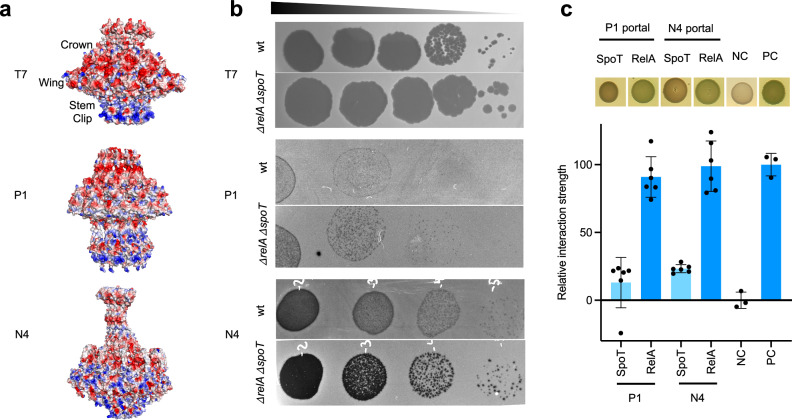
Portal-mediated suppression of the stringent response is conserved among coliphages. **a** Predicted structural models of portal proteins from representative coliphages (T7, P1, N4), colored by electrostatic surface potential, highlighting conserved charge patterning across portals. Due to computational limitations, only half of the portal ring is displayed. **b** Infection efficiency of representative phages assessed by tenfold serial dilution spotting on wild-type and (p)ppGpp⁰ (*ΔrelA ΔspoT*) *E. coli* hosts. Representative plaques from three independent biological replicates are shown. **c** Bacterial two-hybrid assays testing interactions between portal proteins from phages P1 and N4, and the alarmone synthetases RelA and SpoT, on LB agar X-gal plates. NC and PC denote negative and positive controls, respectively. Bar plots show quantification of relative interaction strengths, normalized to the positive control. Error bars represent mean ± s.d. of *n* = 3 or 6 independent colonies. Source data are provided as a Source Data file.

Gp8 is an essential structural component of the phage capsid and is widely conserved among tailed bacteriophages. To determine whether portal-mediated targeting of the SR represents a broader viral strategy, we examined portal proteins from phylogenetically diverse *E. coli* phages in the BASEL collection^40^. Structural predictions indicated that many of these portal proteins assemble into ring-like complexes with predominantly electronegative exterior surfaces, similar to that of T7 Gp8 (Fig. 6a and Supplementary Table 1 and 2).

Consistent with a general role for the stringent response in restricting infection, many of the tested phages (11/18) infected (p)ppGpp⁰ cells more efficiently than wild-type hosts, producing larger or clearer plaques or increased plaque-forming units (Fig. 6b and Supplementary Table 2). Notably, portal proteins from these phages also displayed a conserved pattern of surface charge segregation, with negatively charged exterior regions juxtaposed to discrete positively charged elements (Fig. 6a), suggesting that this electrostatic organization may be a common feature associated with SR sensitivity.

To test whether portal proteins with this charge organization directly engage the alarmone synthesis machinery, we focused on representatives from two unrelated coliphages, P1 and N4. Although these phages differ substantially in genome organization, replication strategy, and virion architecture, their portal proteins form dodecameric ring assemblies analogous to that of T7 Gp8. In both cases, the portal proteins displayed a nonuniform surface charge distribution characterized by a positively charged clip-like region juxtaposed to a predominantly electronegative exterior surface (Fig. 6a). Expression of the P1 and N4 portal proteins was toxic in *E. coli* under conditions requiring SR function, and this toxicity was partially suppressed by RNAP^SR^ alleles (Supplementary Fig. 6a). In quantified LB/X-gal bacterial two-hybrid assays, both portal proteins showed detectable interaction signals with RelA and SpoT, with stronger signals for RelA than for SpoT under these conditions (Fig. 6c and Supplementary Fig. 6b); a MacConkey-based assay gave a similar qualitative pattern (Supplementary Fig. 6c).

These observations support the conclusion that portal proteins from evolutionarily distinct coliphages share related surface charge features and can engage the alarmone synthesis machinery, indicating that portal-mediated modulation of stringent-response signaling is not unique to bacteriophage T7.

## Discussion

The findings reported here indicate that the SR can function not only as a stress adaptation pathway but also as a physiological barrier that constrains phage replication. While the SR has long been recognized for its role in bacterial survival under nutrient limitation and environmental stress, its capacity to globally suppress biosynthesis, transcription, and nucleotide availability also creates an intracellular environment that is unfavorable for viral replication. In contrast to dedicated antiviral defense systems that rely on molecular recognition of invading phages, the SR represents a distinct mode of host defense: rather than detecting phage components directly, it enforces a system-level physiological state that indirectly restricts viral growth by limiting access to essential cellular resources. Such global restriction mechanisms may be particularly effective against diverse phages, as they do not depend on specific recognition motifs that can be readily mutated or evaded.

**Fig. 7:**
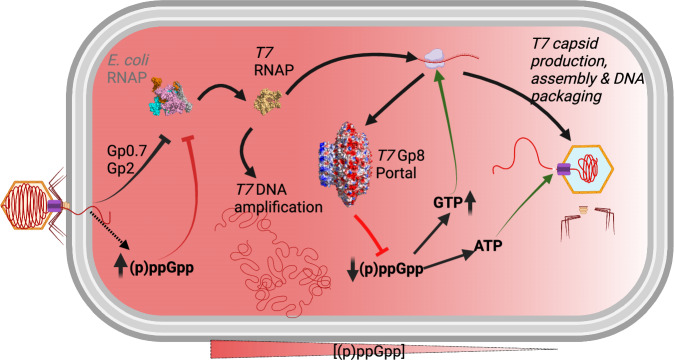
Biphasic model for portal-mediated modulation of the stringent response during phage infection. During early infection, phage entry and host takeover are associated with activation of the stringent response and accumulation of (p)ppGpp. Based on prior literature and the results presented here, we propose that this early alarmone accumulation may help suppress host transcription and favor the transition toward phage-directed gene expression. As infection progresses, the structural portal protein Gp8 accumulates and engages the alarmone synthetases RelA and SpoT, selectively inhibiting (p)ppGpp synthesis. Suppression of alarmone signaling restores nucleotide availability and biosynthetic capacity required for late-stage viral protein synthesis, genome packaging, and lysis. Portal-mediated targeting of stringent-response enzymes was observed for the representative coliphages examined here, suggesting that this mechanism is not unique to T7. (Created in BioRender. ZHANG, Y. (2026) https://BioRender.com/ h71x1zq).

Based on our data and prior literature, we propose a working biphasic model for the role of (p)ppGpp during T7 infection (Fig. 7). Early in infection, phage invasion and host takeover are associated with activation of SR and accumulation of (p)ppGpp. We propose that early alarmone accumulation may contribute to host takeover, as it limits competing cellular processes and facilitates the transition to phage-directed transcription^41^. Notably, (p)ppGpp preferentially inhibits *E. coli* RNAP while having little effect on T7 RNAP^42,43^, allowing phage transcription to proceed efficiently. This effect is reinforced by multiple T7-encoded inhibitors of host RNAP (Gp2^29,31^, Gp5.7^11^, Gp0.7^30^), underscoring the importance of early host transcriptional shutdown for productive infection^41^.

As infection progresses, however, the physiological requirements of the phage shift. Late stages of infection involve massive synthesis of structural proteins and energy-intensive genome packaging by the terminase**-**portal complex, processes that place high demands on cellular amino acids and nucleotide pools^44,45^. Uncontrolled (p)ppGpp accumulation becomes detrimental at this stage, as it depletes GTP and further suppresses their biosynthesis^36–39^. Our data indicate that Gp8-mediated inhibition of RelA and SpoT alleviates this constraint by suppressing (p)ppGpp levels prior to lysis, thereby restoring nucleotide availability and enabling efficient virion assembly and release.

In this framework, the portal protein functions as a molecular gatekeeper that coordinates the transition from early host metabolic repression to late-stage resource mobilization, ensuring optimal timing of lysis and maximal phage yield. This working model also helps reconcile the context-dependent effects of (p)ppGpp observed across phages. Although a majority of the phages tested here (11/18) showed a T7-like pattern consistent with improved infection when SR accumulation is suppressed, a subset of phages, including examples from our BASEL collection panel and from *Pseudomonas putida*^46^, appear to benefit from (p)ppGpp during infection. These cases may reflect a stage-specific advantage of early alarmone accumulation, for example, by promoting host transcriptional repression or metabolic reprogramming during host takeover. More broadly, these considerations suggest that the role of (p)ppGpp during phage infection is unlikely to be uniformly antiviral, but is instead phage-specific, host-specific, and temporally regulated.

The identification of a regulatory role for the phage portal protein is evolutionarily striking. Portal proteins are among the most conserved and structurally constrained components of tailed phages, forming the central channel through which viral DNA is packaged and later delivered into the host. Their essential architectural role would be expected to limit sequence plasticity and functional diversification. Nonetheless, our data reveal that portal proteins have evolved spatially organized, electrostatically defined surface features capable of engaging host regulatory enzymes and modulating global signaling pathways. This suggests that strong selective pressure has favored the coupling of genome delivery and packaging machinery with mechanisms that suppress host stress physiology. Such multifunctionality may ensure that evasion of host physiological restriction is tightly coordinated with late-stage replication demands, minimizing the risk that host defenses interfere with energetically costly assembly and packaging processes. More broadly, these findings expand the conceptual scope of phage anti-defense strategies by demonstrating that essential structural virion components - not only small, dedicated inhibitors - can moonlight as modulators of host stress physiology.

The identification of a regulatory role for the portal protein highlights a broader principle in phage biology: the evolution of multifunctional proteins that target host processes essential under specific physiological conditions. Such functional layering is not unique to Gp8. For example, T7 lysozyme (Gp3.5) not only mediates host cell lysis but also regulates T7 RNAP to coordinate transcription and replication^47,48^. These dual-purpose proteins enable phages to maximize functional output from compact genomes.

Consistent with this idea, our functional screens revealed numerous T7 proteins that are toxic specifically under nutrient-limited conditions but not in rich media. This conditional toxicity suggests that phages encode factors targeting host pathways that become essential during stress, a context frequently encountered in natural and clinical environments but rarely captured in standard laboratory assays. As bacteria in nature often reside in nutrient-poor or otherwise stressful niches, phage strategies that exploit stress-adapted host physiology are likely widespread and ecologically relevant.

The identification of RSH-family toxins such as CapRel in abortive infection systems^12–15^ further underscores the recurrent involvement of alarmone-linked regulatory pathways in phage–host interactions. In these systems, perturbation of translational and nucleotide homeostasis leads to abortive infection outcomes, whereas our findings reveal a complementary viral strategy in which phage portal proteins suppress RelA- and SpoT-mediated (p)ppGpp synthesis during productive infection. Together, these observations suggest that alarmone metabolism constitutes a regulatory interface engaged by both bacterial defense modules and phage countermeasures.

Although our analysis focuses primarily on coliphages, the SR is universal across bacterial phyla, raising the possibility that alarmone signaling may constitute a broader target for viral counter-defense strategies. It remains to be determined whether portal-mediated suppression of (p)ppGpp synthesis extends beyond enterobacterial phages. Moreover, while our purified-component biochemical assays demonstrate that Gp8 inhibits RelA and SpoT synthetase activity without requiring ribosomes or other cellular factors, the precise molecular mechanism underlying this inhibition remains to be resolved. Future structural and interface-mapping studies will be needed to define the relevant binding surfaces, conformational states, and the physiological context of RelA targeting during infection, including its relationship to ribosome-associated activation states. Finally, because (p)ppGpp signaling intersects with multiple defense systems and stress-response pathways, suppression of the SR may indirectly influence other layers of bacterial defense. Dissecting these interactions will be an important avenue for future work aimed at understanding how global physiological defenses integrate into the broader phage**-**host arms race.

## Materials and methods

### Bacterial strains and growth conditions

All *Escherichia coli* strains and primers used in this study are listed in Supplementary Table 3 and 4, respectively. Unless otherwise stated, *E. coli* K-12 MG1655 was used as the wild-type background for infection assays and physiological analyses, and all derivative strains were constructed in this background. *E. coli* DH5α was used for routine cloning, and *E. coli* BL21(DE3) was used for protein expression. Bacteria were routinely grown in lysogeny broth (LB) at 37 °C with aeration, with antibiotics added as required. For nucleotide labeling experiments, cells were grown in defined MOPS minimal medium formulated with low phosphate (0.2 mM K_2_HPO_4_), and 0.2% (v/v) glucose^36^, to minimize background labeling from inorganic phosphate. For experiments in defined nutrient conditions, cells were grown in M9 minimal medium supplemented with glucose (0.2% w/v), glycerol (0.4% v/v) as indicated. Arabinose-inducible expression was achieved by the addition of L-arabinose to a final concentration of 0.2% (w/v). Growth and induction conditions are specified in the corresponding figure legends.

### Phages and propagation

Phages were propagated using *E. coli* MG1655 as host. A 50 mL MG1655 culture in LB supplemented with 1 mM MgCl_2_ and 1 mM CaCl_2_ was grown at 37 °C with shaking at 160 rpm to an OD_600nm_ of ~ 0.5. Phage stock (100 µL; PFU ~ 10^8^−10^10^) was added, and incubation was continued until extensive lysis was observed (1–3 h for T7). The lysate was treated with chloroform (1 mL per 10 mL culture), vortexed, incubated for 5–10 min at room temperature, and centrifuged (5 min, 2430 × *g*). The supernatant was transferred to a new tube, centrifuged again and collected into a clean tube. The phage stock was concentrated, and the buffer was exchanged following the protocol described here^49^. Briefly, a 5 × PEG solution (5 M NaCl, 20% (w/v) PEG800) was added to the cleared lysate and incubated overnight at 4 °C. The precipitated phages were pelleted (1 h, 4 °C, 19000 × *g*), resuspended in 0.5-1 mL phage buffer (150 mM NaCl, 40 mM Tris, 10 mM MgSO_4_), and PFU was determined via plaque assay. Phage stocks were stored at 4 °C.

#### Liquid killing assay

*E. coli* strains MG1655, *∆relA*, and *∆relA∆spoT*, were incubated overnight in LB medium at 37 °C with shaking at 160 rpm. The following day, cultures were backdiluted into fresh LB medium and incubated in flasks at 37 °C with shaking at 160 rpm until reaching an OD_600nm_ of ~ 0.4. Cultures were normalized to OD_600nm_ = 0.4, and 150 µL normalized culture was mixed with T7 phage in a clear flat-bottom 96-well plate to the MOI = 0.1. The OD_600nm_ was recorded via a plate reader (BioTek) every five minutes at 37 °C without shaking.

#### Plaque assay

Plaque assays were performed as described here^50^. Briefly, cultures of the appropriate strains were prepared in LB with appropriate antibiotics and incubated overnight at 37 °C, shaking at 160 rpm. The following day, cultures were adjusted to OD_600nm_ = 2, and 200 µL normalized culture was mixed with 100 µL of 10-fold serially diluted phage and incubated at room temperature for 10 min. The mixtures were then mixed with 3 ml melted 0.7% LB agar (kept at 50 °C) and immediately poured onto prewarmed 1% LB agar plates. Plates were incubated overnight at 37 °C. For time-resolved plaque growth measurements, plates were imaged at 37 °C using the Reshape machine, and plaque diameter expansion over time was quantified using ImageJ.

#### T7 phage engineering

To enable targeted mutagenesis of the essential T7 *gp8* gene, we established a genetic dependency between the host and the phage based on the *trxA* gene, which is a host factor essential for T7 replication. The endogenous *trxA* gene was deleted from the host chromosome, rendering the host non-permissive to wild-type T7 infection. To restore infectivity, *trxA* was introduced into the T7 genome via homologous recombination in place of *gp8*, and the *gp8* was instead provided on the plasmid vector pCA24N^51^ transformed to this *∆trxA* host. This created an interdependent system where the *gp8* sequence on the plasmid could be easily manipulated. Due to homology sequences, the plasmid-encoded *gp8* mutant could integrate into its original position on the T7 genome and thus create a mutant T7 particle that can be counter selected using its independence of a plasmid borne *gp8*.

#### Delete *trxA* in MG1655 using P1 phage transduction

To delete *trxA* from *E. coli* MG1655, a P1 lysate was first prepared using *E. coli ∆trxA* strain from the Keio collection^52^. Then, the P1 transduction method was used to generate an MG1655 *∆trxA::kan* strain^53^, and the kanamycin resistance marker was flipped out by using pCP20^54^.

#### Replace *gp8* with *trxA* in T7

The *trxA* gene was PCR-amplified using genomic DNA from *E. coli* strain MG1655 as a template and primers pYZ1189 and pYZ1190, each containing 40 bp extensions homologous to sequences flanking the *gp8* locus in the T7 phage genome. The purified PCR product was introduced into *E. coli* MG1655 cells by electroporation during co-infection with T7 phage. Immediately following electroporation, LB medium was added, and the culture was incubated at 37 °C, 160 rpm until lysis was observed (around 1.5 h). Phage lysates were then harvested with chloroform and clarified via centrifugation (5 min, 430 × *g*).

#### Cloning of pCA24N-*gp8* and quick-change mutagenesis of *gp8*

The *gp8* gene, as well as 350 bp flanking regions from T7, was PCR amplified using primers pYZ1191 and pYZ1192 that also encoded an *XhoI* and HindIII restriction site, respectively. This PCR product, as well as the pCA24N^51^ plasmid were both digested with *XhoI* and HindIII and ligated together using T4 ligase. The constructed pCA24N-*gp8* was purified and used as a template for quick-change mutagenesis with primers pYZ1085-pYZ1136 (see Primer list).

#### Constructing mutant T7 phages with *gp8* mutations

pCA24N plasmids encoding mutant *gp8* were purified and transformed into *E. coli* MG1655 *∆trxA*. This strain was then infected with T7 *gp8::trxA* phage lysate (prepared as described above). Through homologous recombination between the plasmid-encoded *gp8* mutant and the corresponding flanking regions in the T7 genome (where *trxA* is inserted), the mutant *gp8* sequence can replace *trxA*, and results in T7 progeny that carry the desired *gp8* mutation. The mutant T7 phage particles can be counter selected using its independence of the pCA24N-*gp8*.

### Measurement of (p)ppGpp by radiolabeling and autoradiography

(p)ppGpp is a very labile molecule not suited for analysis by HPLC or mass spectrometry^55^. We therefore performed thin- layer chromatography (TLC) to quantify both pppGpp and ppGpp. As phosphates in the medium impair efficient incorporation of H_3_^32^PO_4_ during labeling, MOPS medium supplemented with low phosphate concentration (0.2 mM K_2_HPO_4_), and 0.2% (v/v) glucose was used^36^. MG1655 was grown for ca. 18 hrs in MOPS-lowP_i_-Glc medium at 37 °C with agitation (160 rpm). Afterwards, the strains were adjusted to OD_600nm_ = 0.05 in MOPS-lowP_i_-Glc and grown at 37 °C with agitation (160 rpm) until OD_600nm_ of 0.2-0.3 was reached. Then, cells were transferred to a 2-ml Eppendorf tube and H_3_^32^PO_4_ (equal to 100 μCi/ml) (Revvity, NEX053001MC) was added. After 1.5 h at 37 °C with 600 rpm agitation, cells were transferred to a new tube and infected with T7 phage to an MOI of 0.1. Immediately after, 150 µl infected cells were transferred to a clear 96-well plate and OD_600nm_ at 37 °C was monitored every 2 min via a plate reader (BioTek). Simultaneously, 25 µl sample was collected every 2 min and transferred to 5 µl cold 2 M formic acid. Samples were stored at − 20 °C until analyzed by TLC using 1.5 M KH_2_PO_4_ (pH 3.4) as mobile phase.

### Cloning all T7 genes into the pET28b and pBAD33 vectors

Genomic DNA of *E. coli* T7 bacteriophage was used as the template for amplification of individual T7 genes with Q5 High-Fidelity DNA polymerase (New England Biolabs). PCR amplicons were gel-purified, quantified spectrophotometrically, and digested with EcoRI and *NcoI* at 37 °C for 3 h followed by enzyme inactivation at 80 °C for 20 min. The digested fragments were ligated into the similarly treated pET28b vector using T4 DNA ligase at room temperature for 30 min. Ligation mixtures were transformed into chemically competent *E. coli* DH5α cells and plated on LB agar supplemented with kanamycin (25 µg mL⁻¹). Individual transformants were screened by colony PCR, and clones displaying expected insert sizes were cultured in LB medium for plasmid purification. All constructs were verified by Sanger sequencing, yielding a pET28b**-**T7 gene library encompassing 51 of 59 targeted genes; 8 highly toxic genes could not be cloned or yielded unstable plasmids.

To place the T7 genes under the arabinose-inducible promoter, verified pET**-**T7 plasmids were digested with XbaI, HindIII and *EcoRV*, and ligated into pBAD33 linearized with XbaI and HindIII. This procedure transferred the pET Shine-Dalgarno sequence and corresponding T7 gene inserts into pBAD33 under the control of the pBAD promoter. Using the iVEC cloning strategy^56^, we successfully obtained pBAD33-*gp0.4* and pBAD33-*gp0.7* plasmids, the pET versions of which were impossible. The resulting library, designated pBAD**-**T7, comprised 53 T7 genes. For expression of T7 proteins in strain YZ921, the T7 genes were subcloned from the pBAD33 (cat) vector into the pBAD30 (bla) vector using the XbaI and HindIII restriction sites, as YZ921 carries a chloramphenicol resistance gene (cat).

### Toxicity assay of pBAD33-T7 genes on five media

Each of the 53 verified pBAD**-**T7 plasmids was individually transformed into chemically competent *E. coli* MG1655 cells. Transformants were streaked as single colonies on LB agar plates supplemented with chloramphenicol (25 µg mL⁻¹) and a single colony from each transformant was inoculated into 1 mL LB broth and cultured overnight at 37 °C with shaking (200 rpm). From each culture, 300 µL was diluted in 800 µL fresh LB to measure optical density at 600 nm (OD_600nm_), while another 300 µL aliquot was pelleted by centrifugation at 15,871 × *g* for 3 min, washed twice with 1 mL PBS, and resuspended to an OD_600nm_ = 1. Serial tenfold dilutions (10⁸**-**10¹ CFU mL⁻¹) were prepared in 96-well plates by mixing 20 µL of bacterial suspension with 180 µL PBS per well. From each dilution, 5 µL was spotted onto five pre-dried chloramphenicol-containing agar media: LB + 0.4% glucose, LB + 0.2% L-arabinose, M9 + 0.4% glucose, M9 + 0.4% glycerol, and M9 + 0.4% glycerol + 0.2% L-arabinose. Plates were incubated at 37 °C and photographed every 24 h to monitor colony growth and induction-dependent phenotypic changes.

### Construction of bacterial two-hybrid vectors

#### Cloning *relA*, *spoT* and their truncates

*spoT* and *relA* were cloned into the bacterial two-hybrid vectors pUT18C and pKT25. The full-length genes were amplified from *E. coli* MG1655 chromosomal DNA using primer pairs pYZ479/pYZ480 (*spoT*) and pYZ481/pYZ482 (*relA*). PCR products and recipient plasmids were digested with XbaI and XmaI, ligated, and transformed into *E. coli*. Truncated *spoT* variants corresponding to the HS, HST, TCA, and CA regions were generated by PCR using primer pairs pYZ473/pYZ474, pYZ473/pYZ475, pYZ477/pYZ476, and pYZ478/pYZ476, respectively. The resulting fragments were digested with XbaI/XmaI and cloned into pUT18C or pKT25. Truncated *relA* variants (HS, HST, TCA, and CA) were amplified using primer pairs pYZ917/pYZ919, pYZ917/pYZ918, pYZ920/pYZ921, and pYZ920/pYZ922, respectively, digested with XbaI/XmaI, and cloned into pUT18C.

#### Cloning of the portal protein from phages P1 and N4 into pKT25

Portal protein from phages P1 and N4 were amplified using primer pairs pYZ1285/pYZ1286 and pYZ1287/pYZ1288 for phage P1 and N4, respectively. The pKT25 was digested with KpnI and EcoRI, assembled with both PCR products by the iVEC cloning method^56^.

#### pUT18-*rpoZ* and pKNT25-*rpoZ*

To generate pUT18-*rpoZ*, the full-length *rpoZ* gene was PCR-amplified from *E. coli* MG1655 genomic DNA using primers pYZ867 and pYZ868. The amplified fragment and pUT18 vector were digested with HindIII and XbaI, ligated, and transformed into *E. coli* DH5α. For the construction of pKNT25-*rpoZ*, the pKNT25 vector was first modified by site-directed mutagenesis using primers pYZ871 and pYZ872 following the QuickChange protocol to introduce an *NcoI* restriction site, generating pKNT25-*NcoI*. The *rpoZ* insert was excised from pUT18-rpoZ by digestion with *ScaI*, HindIII, and XbaI, and ligated into HindIII/XbaI-digested pKNT25-*NcoI*.

#### Cloning of pUT18/KT25-*gp4.2/gp1.5/gp8/gp6.5*

An *NcoI* restriction site was introduced into pUT18C (YZ709) by site-directed mutagenesis using primers pYZ864 and pYZ866, generating pUT18C-*NcoI*. For pKT25 (YZ705), the native *NcoI* site was first removed using primers pYZ863 and pYZ862, followed by the introduction of a new *NcoI* site using primers pYZ864 and pYZ865 to generate pKT25-*NcoI*. Plasmids pET28-*gp4.2*, pET28-*gp1.5*, pET28-*gp8*, and pET28-*gp6.5* were digested with EcoRI, *NcoI*, and HindIII, and the resulting inserts were individually cloned into pUT18C-*NcoI* or pKT25-*NcoI* vectors digested with *NcoI* and HindIII.

#### Construction of Gp8 truncations

Truncations of Gp8 were generated by site-directed mutagenesis of the *gp8* gene on the pKT25 vector in *E. coli* DH5a using the QuickChange method. Primer pairs pYZ1063/pYZ1064, pYZ1065/pYZ1066, pYZ1067/pYZ1068, and pYZ1069/pYZ1070 were used to construct the clip truncation, clip+stem truncation, N-terminal truncation, and C-terminal truncation, respectively. PCR reactions were carried out with Q5 DNA polymerase using an extension time of 3 min and an annealing temperature gradient of 50–70 °C across eight 12 µL samples. After amplification, the samples were pooled and digested with 2.5 µL *DpnI* at 37 °C for 3 h, followed by inactivation at 80 °C for 20 min. The reaction mixture was then transformed into *E. coli* DH5a via chemical transformation. Successful truncations were confirmed by Sanger sequencing using primers pYZ471 and pYZ472.

### Bacterial two-hybrid assay

The bacterial two-hybrid assay was performed using *Escherichia coli* strain BTH101. Cells were co-transformed with plasmids pUT18C and pKT25, with one plasmid encoding either *relA* or *spoT* and the other encoding the phage gene of interest. Transformants were selected on LB agar supplemented with 0.2% (w/v) glucose, 25 µg mL⁻¹ kanamycin, and 100 µg mL⁻¹ ampicillin and incubated at 37 °C. Single colonies were used to inoculate overnight cultures grown at 37 °C with shaking at 170 rpm. The following day, cultures were washed twice with phosphate-buffered saline (PBS) and resuspended in PBS to a final optical density of OD_600nm_ = 5. Aliquots (5 µL) of the normalized cell suspensions were spotted onto LB agar containing 25 µg mL⁻¹ kanamycin, 100 µg mL⁻¹ ampicillin, and 40 µg mL⁻¹ X-gal. Plates were incubated at 30 °C overnight. Plates were scanned, and spot intensities were quantified using ImageJ. Images were converted to RGB stacks, the red channel was isolated and inverted, and the mean intensity of each spot was measured. Background signal from negative controls was subtracted from all samples. Interaction strengths were normalized and expressed as a percentage of the positive control or full-length protein interaction, as specified in the figure legends. For the MacConkey-based bacterial two-hybrid assay shown only as a supplementary qualitative readout (Supplementary Fig. 6c), the same normalized cultures were spotted onto MacConkey agar supplemented with maltose, kanamycin and ampicillin, and incubated at 30 °C until color development was observed.

### Cloning of the pET28-HMS-relA and -spoT vectors

#### pET28-HMS-*spoT* and derivatives

Plasmids pET28a and pMAL-c2x were digested with *NdeI* and EcoRI and ligated to generate pET28-His₆-MBP (YZ913). The *sumo* and *spoT* genes were PCR-amplified using primers pYZ692/693 and pYZ694/695, fused by overlap PCR, and cloned into pET28a using *NcoI* and HindIII to generate pET28a-His₆-SUMO-SpoT (YZ307). The SUMO-SpoT fragment was excised with EcoRI and HindIII and ligated into pET28a-His₆-MBP. Missing SUMO sequences were amplified using primers pYZ684/685, digested with EcoRI, and inserted into this intermediate construct to generate pET28-His₆-MBP-SUMO-SpoT (pET28-HMS-spoT).

#### Generation of SpoT point mutants

The E319Q and H72A/D73A mutations were introduced into pET28-HMS-spoT using PCR-amplified fragments derived from chromosomal *spoT* mutant strains YZ586 and YZ905, respectively^57^. PCR products generated with primers pYZ702 and pYZ703 were digested with *PmlI* and *SalI* and ligated into pET28-HMS-spoT to generate the corresponding mutant constructs. The *spoT* gene was deleted from pET28-HMS-spoT using primers pYZ905 and pYZ906 to generate the pET28b-HMS vector (YZ1515).

#### Construction of pET28-HMS-*relA*

The *relA* gene was PCR-amplified from *E. coli* MG1655 using primers pYZ927 and pYZ928. Both the PCR product and pET28b-HMS were digested with *BamHI* and HindIII, ligated using T4 DNA ligase, and transformed into *E. coli* DH5α to generate pET28-HMS-relA.

### Pull down and Western blot

Overnight cultures of *E. coli* BL21 DE3 strains expressing pRARE, pET28b-HMS, -*relA* or -*spoT*, and pBAD30-*gp8-Flag* (see Strain list) were incubated in LB medium with 25 µg/mL kanamycin, 25 µg/mL chloramphenicol, and 30 µg/mL ampicillin at 37 °C with shaking at 160 rpm. The following day, cultures were back-diluted to an OD_600nm_ of 0.05 in 50 mL fresh LB medium containing the same antibiotics as above and incubated at 37 °C with shaking at 160 rpm until reaching OD_600nm_ of ~ 0.2-0.3. Gene expression was induced by the addition of arabinose and IPTG to final concentrations of 0.2% and 0.5 mM, respectively, followed by incubation for 4 h at 37 °C with shaking at 160 rpm. Cells were harvested by centrifugation (10 min, 1932 × *g*, 4 °C), resuspended in 10 mL PBS and centrifuged again as above. Supernatant was discarded, and cell pellets were stored at − 20 °C. Pellets were thawed on ice, resuspended in 400 µL lysis buffer (50 mM HEPES (pH 8), 0.25 M NaCl, 10 mM imidazole, 200 µM ZnCl_2_, and 2 mM MgCl_2_) and disrupted with glass beads (Sigma, G4649) using a MagNA Lyser (20 s at 6500 rpm, done twice with cooling on ice between runs). Lysates were centrifuged (5 min, 2348 × *g*, 4 °C) and supernatants were collected. Total protein concentration was measured via Bradford Assay, and samples were normalized with lysis buffer to equal total protein concentrations. Equal volumes of normalized lysates were incubated with 50 µL Ni-NTA Agarose resins (Qiagen) overnight at 4 °C with gentle inversion. The next day, samples were centrifuged (2 min, 94 × *g*, 4 °C), and supernatant was removed. Resins were gently resuspended with 1 mL lysis buffer and incubated for 1 h at 4 °C with gentle inversion. Samples were centrifuged (2 min, 94 × *g*, 4 °C), and the supernatant was removed. Resins were subsequently resuspended in 1 mL wash buffer (50 mM HEPES (pH 8), 0.25 M NaCl, 20 mM imidazole, 200 µM ZnCl_2_, 2 mM MgCl_2_) and incubated for 1 h at 4 °C with gentle inversion. Following incubation, samples were transferred to a 0.5 mL Eppendorf tube and centrifuged (first for 2 min at 94 × *g*, then for 1 min at 15,871 × *g*). Supernatant was removed, and proteins were eluted with 20 µL elution buffer (50 mM HEPES (pH 8), 0.25 M NaCl, 1.25 M imidazole, 200 µM ZnCl_2_, 2 mM MgCl_2_) to yield a final imidazole concentration of approximately 500 mM. Samples were incubated for 15 min at 4 °C (gently disturbing the resins occasionally). Samples were then centrifuged (2 min, 94 × *g*), and 35 µL supernatant was transferred to 11.6 µL 4x Laemmli buffer, boiled for 15 min at 95 °C and 12 µL of each sample was loaded onto a 10% SDS-PAGE gel. Following electrophoresis, proteins were transferred to a PVDF membrane by wet transfer (300 mA, 1 h, 4 °C) using transfer buffer composed of 20% methanol, 0.2 M glycine, and 25 mM Tris (pH 8.3). Membrane was blocked overnight at 4 °C in TBST + 5% (w/v) milk powder. Next day, the membrane was incubated with ANTI-FLAG® M2 antibody (mouse monoclonal, Merck) diluted (1:2000) in TBST + 5% (w/v) milk powder for 1 h, 4 °C, washed three times (10 min each) in TBST, and subsequently incubated with anti-mouse secondary antibody in TBST + 5% (w/v) milk powder for 1 h, 4 °C. After three additional washes in TBST, the membrane was developed using Pierce^®^ ECL Western Blotting Substrate (Thermo Scientific, #32209) and imaged via ImageQuant LAS4000.

### Protein purification for biochemical assays

RelA (YZ1607), SpoT hydrolysis only (HYD, YZ1063), SpoT synthesis only (SYN, YZ1070), and Gp8 (YZ1632) constructs bearing an N-terminal His**-**MBP**-**SUMO (HMS) tag were expressed from pET28b in *E. coli* BL21(DE3). Overnight cultures of these strains were grown in LB medium supplemented with appropriate antibiotics at 37 °C, with shaking at 160 rpm. The following day, cultures were back-diluted 1:50 into 0.5 L fresh LB medium supplemented with appropriate antibiotics and grown at 37 °C with shaking at 160 rpm until reaching an OD of 0.4–0.7. Protein expression was induced by the addition of IPTG to a final concentration of 0.1 mM, and cultures were incubated overnight at 24 °C, shaking with aeration. The next day, cells were harvested by centrifugation (6227 × *g*, 10 min, 4 °C), washed in 30 mL cold PBS, and pelleted again (1932 × *g*, 20 min, 4 °C). Resulting pellets were resuspended in 40 mL cold lysis buffer (50 mM Tris-HCl pH = 8, 1 M NaCl, 2 mM DTT, 10 mM imidazole) supplemented with Pierce^TM^ Protease Inhibitor Mini (Thermo Scientific, A32955). Cells were lysed by sonication on ice for 24 min at 20% amplitude, and lysates were cleared by centrifugation (23,666 × *g* for 45 min at 4 °C). Cleared lysates were incubated with 0.5-1 mL Ni-NTA Agarose resins (Qiagen) overnight at 4 °C with gentle inversion. The next day, the resins were collected by centrifugation (169 × *g*, 2 min) and washed once with 30 mL lysis buffer (1 h, 4 °C), followed by 30 mL wash buffer (same composition as lysis buffer, except imidazole increased to 20 mM). Resins were transferred onto a Bio-Rad Gravity Flow Column, and bound proteins were eluted with 700 µL elution buffer (50 mM tris-HCl pH = 8, 1 M NaCl, 2 mM DTT, 500 mM imidazole). The eluted fraction was subjected to size-exclusion chromatography using an ӒKTA Pure^TM^ system (Cytiva) equilibrated gel filtration buffer (50 mM HEPES pH = 8, 1 M NaCl, 2 mM DTT, 5% glycerol). Protein concentrations were determined via Bradford Assay.

### Synthesis of [α-³²P](p)ppGpp via RelSeq

[α-³²P](p)ppGpp was synthesized using [α-³²P]GTP (Revvity, BLU006X250UC) as substrate in a RelSeq-catalyzed reaction^58^. RelSeq (YZ8) was expressed and purified as described above, except that protein expression was induced with 0.5 mM IPTG for 3 h at 30 °C. The purification buffer contained 500 mM NaCl, 50 mM Tris-HCl (pH 8), and 5% glycerol, with imidazole concentrations of 20, 40, and 500 mM for lysis, wash, and elution buffers, respectively. The gel filtration buffer was identical, but without imidazole. For the enzyme reaction, a 10 × reaction buffer was prepared containing 250 mM Tris (pH 9), 1 M NaCl, and 150 mM MgCl_2_. The final reaction system contained 1 × reaction buffer, 8 mM ATP, 125 nM [α-³²P]GTP, and 4 µM RelSeq, and was incubated at 37 °C for 1 h. The reaction was terminated by boiling for 5 min at 95 °C, followed by centrifugation (20,238 × *g*, 5 min). Conversion of substrate was verified by thin-layer chromatography (TLC) using 0.5 µL of the reaction mixture and 1.5 M KH_2_PO_4_ (pH 3.4) as the mobile phase.

### Biochemical assays

Synthetase activity of RelA and SpoT (SYN) was assayed in reaction mixtures containing 50 mM HEPES (pH 7.4), 100 mM KCl, 5 mM MgCl_2_, 2 mM DTT, 0.2 mg/mL BSA, 2 mM GTP, and 6 nM [α-³²P] GTP (Revvity, BLU006X250UC) were prepared. Protein was added to final concentrations of 0.5 µM (RelA) or 3 µM (SpoT (SYN)). Gp8 was added to varying final concentrations, and samples were incubated at room temperature for 10 min before enzyme reactions were initiated by the addition of 5 mM ATP. Hydrolase activity of SpoT (HYD) was assayed in a reaction mixture containing 50 mM HEPES (pH 8), 100 mM KCl, 5 mM MnCl_2_, 2 mM DTT, and 0.2 mg/mL BSA was prepared. SpoT (HYD) was added to a final concentration of 100 nM, while Gp8 was added at varying final concentrations. Reaction mixtures were incubated at room temperature for 10 min before enzyme reaction was initiated by the simultaneous addition of cold ppGpp (Jena Bioscience, NU-884L), and hot [α-³²P](p)ppGpp to final concentrations of 31.25 and 6 nM, respectively. Enzyme reactions were stopped by transferring 4 µL of the reaction mixtures into 1 µL cold 250 mM EDTA (final concentration 50 mM). Samples were loaded onto a PEI cellulose plate and resolved using 1.5 M KH_2_PO_4_ (pH 3.4) as the mobile phase. Plates were exposed to a BAS-MS imaging plate and scanned using a Typhoon phosphorimager.

### Structural analysis and AlphaFold modeling

For structural analyses of phage portal proteins, experimentally determined structures were used where available: T5 portal protein (PDB: 9ILP), T7 portal protein (PDB: 7EY6), and HK97 portal protein (PDB: 8CEZ). For portal proteins without available experimental structures, models were generated using AlphaFold 3^59^. Because of computational limitations associated with modeling the full dodecameric portal ring, six protomers were modeled to represent one half of the ring assembly. Portal protein sequences from the BASEL phage collection were obtained from the NCBI GenBank accessions reported by Maffei et al.^40^, with the exception of Bas66/T3, which was retrieved from Swiss-Model. AlphaFold 3 models for the BASEL phage portal proteins were generated/accessed on 17 January 2025. For phages T2, P1, and N4, portal protein sequences were retrieved from NCBI Reference Sequences YP_010073821.1, YP_006480.1, and YP_950537.1, respectively, and AlphaFold 3 models were generated/accessed on 20 January 2025. The corresponding UniProt identifiers for the AlphaFold-modeled portal proteins were as follows: T2, A0A2Z5WLT7; P1, Q71TR7; N4, A0MZE1; Bas02, A0AAE7VVD2; Bas11, A0AAE8B112; Bas12, A0AAE7VPV7; Bas17, A0AAE7VVV2; Bas23, A0AAE7W1U1; Bas26, A0AAE7VSI8; Bas51, A0AAE8B8L0; Bas63, A0AAE7VVG7; Bas64, A0AAE8B0S6; Bas65, A0AAE8B0K4; Bas66/T3, Q778N3 or P20323; Bas68, A0AAE7VPU5; and Bas69, QXV75784, corresponding to UniParc entry UPI001EA6E945. For predicted structures, model confidence was assessed using predicted local distance difference test (pLDDT) scores and predicted aligned error (PAE) matrices. Per-residue pLDDT values, encoded in the B-factor field of the AlphaFold output models, were visualized in PyMOL (ver. 2.6.2) using the B-factor putty representation and rendered from two orientations for each model. PAE matrices were extracted from the AlphaFold output files and visualized using PAE Viewer^60^ (accessed on 19 May 2026). The resulting pLDDT-based structural views and corresponding PAE matrices are provided in Supplementary Table 1.

### Statistics and reproducibility

For Western blots, SDS-PAGE gels, TLC plates, and plate images, representative results from at least two independent biological replicates are shown.

### Reporting summary

Further information on research design is available in the Nature Portfolio Reporting Summary linked to this article.

## Supplementary information

Supplementary information

Reporting Summary

Transparent Peer Review file

## Source data

Source data

## Data Availability

All data that support the findings of this study are available in the article, its supplementary materials, as well as in the Source Data file. Source data are provided in this paper.

## References

[1] Egido, J. E., Costa, A. R., Aparicio-Maldonado, C., Haas, P. J. & Brouns, S. J. J. Mechanisms and clinical importance of bacteriophage resistance. *FEMS Microbiol. Rev.* **46**, 10.1093/femsre/fuab048 (2022). 10.1093/femsre/fuab048 PMC8829019 34558600

[2] Georjon, H. & Bernheim, A. The highly diverse antiphage defence systems of bacteria. *Nat. Rev. Microbiol.* **21**, 686–700 (2023). 37460672 10.1038/s41579-023-00934-x

[3] Hochhauser, D. & Sorek, R. Manipulation of the nucleotide pool in human, bacterial and plant immunity. *Nat. Rev. Immunol.* **26**, 7–22 (2026). 40730656 10.1038/s41577-025-01206-w

[4] Bernheim, A. & Sorek, R. The pan-immune system of bacteria: antiviral defence as a community resource. *Nat. Rev. Microbiol* **18**, 113–119 (2020). 31695182 10.1038/s41579-019-0278-2

[5] Lopatina, A., Tal, N. & Sorek, R. Abortive infection: bacterial suicide as an antiviral immune strategy. *Annu. Rev. Virol.* **7**, 371–384 (2020). 32559405 10.1146/annurev-virology-011620-040628

[6] Potrykus, K. & Cashel, M. (p)ppGpp: still magical? *Annu. Rev. Microbiol.* **62**, 35–51 (2008). 10.1146/annurev.micro.62.081307.162903 18454629

[7] Irving, S. E., Choudhury, N. R. & Corrigan, R. M. The stringent response and physiological roles of (pp)pGpp in bacteria. *Nat. Rev. Microbiol.* **19**, 256–271 (2021). 33149273 10.1038/s41579-020-00470-y

[8] Hauryliuk, V., Atkinson, G. C., Murakami, K. S., Tenson, T. & Gerdes, K. Recent functional insights into the role of (p)ppGpp in bacterial physiology. *Nat. Rev. Microbiol.* **13**, 298–309 (2015). 25853779 10.1038/nrmicro3448 PMC4659695

[9] Nowicki, D., Kobiela, W., Wegrzyn, A., Wegrzyn, G. & Szalewska-Palasz, A. ppGpp-dependent negative control of DNA replication of Shiga toxin-converting bacteriophages in Escherichia coli. *J. Bacteriol.* **195**, 5007–5015 (2013). 23995636 10.1128/JB.00592-13 PMC3811577

[10] Bryan, D., El-Shibiny, A., Hobbs, Z., Porter, J. & Kutter, E. M. Bacteriophage T4 infection of stationary phase E. coli: life after log from a phage perspective. *Front Microbiol.* **7**, 1391 (2016). 10.3389/fmicb.2016.01391 PMC5014867 27660625

[11] Tabib-Salazar, A. et al. T7 phage factor required for managing RpoS in Escherichia coli. *Proc. Natl. Acad. Sci. USA* **115**, E5353–E5362 (2018). 29789383 10.1073/pnas.1800429115 PMC6003314

[12] Zhang et al. Direct activation of a bacterial innate immune system by a viral capsid protein. *Nature* **612**, 132–140 (2022). 36385533 10.1038/s41586-022-05444-z PMC9712102

[13] Jimmy, S. et al. A widespread toxin-antitoxin system exploiting growth control via alarmone signaling. *Proc. Natl. Acad. Sci. USA* **117**, 10500–10510 (2020). 32345719 10.1073/pnas.1916617117 PMC7229694

[14] Kurata, T. et al. RelA-SpoT Homolog toxins pyrophosphorylate the CCA end of tRNA to inhibit protein synthesis. *Mol. Cell* **81**, 3160–3170 (2021). 34174184 10.1016/j.molcel.2021.06.005

[15] Zhang, T. et al. A bacterial immunity protein directly senses two disparate phage proteins. *Nature* **635**, 728–735 (2024). 39415022 10.1038/s41586-024-08039-y PMC11578894

[16] Ho, P. et al. Bacteriophage antidefense genes that neutralize TIR and STING immune responses. *Cell Rep.* **42**, 112305 (2023). 36952342 10.1016/j.celrep.2023.112305

[17] Niault, T., van Houte, S., Westra, E. & Swarts, D. C. Evolution and ecology of anti-defence systems in phages and plasmids. *Curr. Biol.* **35**, R32–R44 (2025). 39765217 10.1016/j.cub.2024.11.033

[18] Murtazalieva, K., Mu, A., Petrovskaya, A. & Finn, R. D. The growing repertoire of phage anti-defence systems. *Trends Microbiol.* **32**, 1212–1228 (2024). 38845267 10.1016/j.tim.2024.05.005

[19] Haseltine, W. A. & Block, R. Synthesis of guanosine tetra- and pentaphosphate requires the presence of a codon-specific, uncharged transfer ribonucleic acid in the acceptor site of ribosomes. *Proc. Natl. Acad. Sci. USA* **70**, 1564–1568 (1973). 4576025 10.1073/pnas.70.5.1564 PMC433543

[20] Xiao, H. et al. Residual guanosine 3’,5’-bispyrophosphate synthetic activity of relA null mutants can be eliminated by spoT null mutations. *J. Biol. Chem.* **266**, 5980–5990 (1991). 2005134

[21] Fernández-Coll, L. & Cashel, M. Possible Roles for Basal Levels of (p)ppGpp: Growth Efficiency Vs. Surviving Stress. *Front. Microbiol.* **11**, 592718 (2020). 33162969 10.3389/fmicb.2020.592718 PMC7581894

[22] Vinella, D., Albrecht, C., Cashel, M. & D’Ari, R. Iron limitation induces SpoT-dependent accumulation of ppGpp in Escherichia coli. *Mol. Microbiol.* **56**, 958–970 (2005). 15853883 10.1111/j.1365-2958.2005.04601.x

[23] Spira, B., Silberstein, N. & Yagil, E. Guanosine 3’,5’-bispyrophosphate (ppGpp) synthesis in cells of Escherichia coli starved for Pi. *J. Bacteriol.* **177**, 4053–4058 (1995). 7608079 10.1128/jb.177.14.4053-4058.1995 PMC177136

[24] Ross, W., Vrentas, C. E., Sanchez-Vazquez, P., Gaal, T. & Gourse, R. L. The magic spot: a ppGpp binding site on E. coli RNA polymerase responsible for regulation of transcription initiation. *Mol. Cell* **50**, 420–429 (2013). 23623682 10.1016/j.molcel.2013.03.021 PMC3654024

[25] Ross, W. et al. ppGpp Binding to a Site at the RNAP-DksA Interface Accounts for Its Dramatic Effects on Transcription Initiation during the Stringent Response. *Mol. Cell* **62**, 811–823 (2016). 27237053 10.1016/j.molcel.2016.04.029 PMC4912440

[26] Sanchez-Vazquez, P., Dewey, C. N., Kitten, N., Ross, W. & Gourse, R. L. Genome-wide effects on Escherichia coli transcription from ppGpp binding to its two sites on RNA polymerase. *Proc. Natl. Acad. Sci. USA* **116**, 8310–8319 (2019). 30971496 10.1073/pnas.1819682116 PMC6486775

[27] Durfee, T., Hansen, A. M., Zhi, H., Blattner, F. R. & Jin, D. J. Transcription profiling of the stringent response in Escherichia coli. *J. Bacteriol.* **190**, 1084–1096 (2008). 18039766 10.1128/JB.01092-07 PMC2223561

[28] Traxler, M. F. et al. The global, ppGpp-mediated stringent response to amino acid starvation in Escherichia coli. *Mol. Microbiol.* **68**, 1128–1148 (2008). 18430135 10.1111/j.1365-2958.2008.06229.x PMC3719176

[29] Bae, B. et al. Phage T7 Gp2 inhibition of Escherichia coli RNA polymerase involves misappropriation of sigma70 domain 1.1. *Proc. Natl. Acad. Sci. USA* **110**, 19772–19777 (2013). 24218560 10.1073/pnas.1314576110 PMC3856789

[30] Severinova, E. & Severinov, K. Localization of the Escherichia coli RNA polymerase beta’ subunit residue phosphorylated by bacteriophage T7 kinase Gp0.7. *J. Bacteriol.* **188**, 3470–3476 (2006). 16672600 10.1128/JB.188.10.3470-3476.2006 PMC1482854

[31] Savalia, D., Robins, W., Nechaev, S., Molineux, I. & Severinov, K. The role of the T7 Gp2 inhibitor of host RNA polymerase in phage development. *J. Mol. Biol.* **402**, 118–126 (2010). 20650282 10.1016/j.jmb.2010.07.012 PMC2941873

[32] Sarubbi, E., Rudd, K. E. & Cashel, M. Basal ppGpp level adjustment shown by new spoT mutants affect steady state growth rates and rrnA ribosomal promoter regulation in Escherichia coli. *Mol. Gen. Genet.* **213**, 214–222 (1988). 2460731 10.1007/BF00339584

[33] Grucela, P. K. & Zhang, Y. E. Basal level of ppGpp coordinates Escherichia coli cell heterogeneity and ampicillin resistance and persistence. *Micro. Cell* **10**, 248–260 (2023). 10.15698/mic2023.11.808 PMC10625690 37933276

[34] Gray Michael, J. Inorganic polyphosphate accumulation in Escherichia coli Is Regulated by DksA but Not by (p)ppGpp.* J. Bacteriol.*** 201**, 10.1128/jb.00664-00618 (2019). 10.1128/JB.00664-18 PMC6456864 30745375

[35] Gray Michael, J. et al. Interactions between DksA and stress-responsive alternative sigma factors control inorganic polyphosphate accumulation in Escherichia coli.* J. Bacteriol.*** 202**, 10.1128/jb.00133-00120 (2020). 10.1128/JB.00133-20 PMC7317045 32341074

[36] Zhang, Y. E. et al. (p)ppGpp Regulates a bacterial nucleosidase by an allosteric two-domain switch. *Mol. Cell* **74**, 1239–1249 (2019). 31023582 10.1016/j.molcel.2019.03.035

[37] Grucela, P. K., Fuhrer, T., Sauer, U., Chao, Y. & Zhang, Y. E. Ribose 5-phosphate: the key metabolite bridging the metabolisms of nucleotides and amino acids during stringent response in Escherichia coli? *Micro. Cell* **10**, 141–144 (2023). 10.15698/mic2023.07.799 PMC10311079 37395996

[38] Wang, B. et al. Affinity-based capture and identification of protein effectors of the growth regulator ppGpp. *Nat. Chem. Biol.* **15**, 141–150 (2019). 30559427 10.1038/s41589-018-0183-4 PMC6366861

[39] Wang, B., Grant, R. A. & Laub, M. T. ppGpp Coordinates Nucleotide and Amino-Acid Synthesis in E. coli During Starvation. *Mol. Cell* **80**, 29–42 (2020). 32857952 10.1016/j.molcel.2020.08.005 PMC8362273

[40] Maffei, E. et al. Systematic exploration of Escherichia coli phage-host interactions with the BASEL phage collection. *PLoS Biol.* **19**, e3001424 (2021). 34784345 10.1371/journal.pbio.3001424 PMC8594841

[41] Qimron, U., Kulczyk, A. W., Hamdan, S. M., Tabor, S. & Richardson, C. C. Inadequate inhibition of host RNA polymerase restricts T7 bacteriophage growth on hosts overexpressing udk. *Mol. Microbiol.* **67**, 448–457 (2008). 18067538 10.1111/j.1365-2958.2007.06058.x

[42] Kingston, R. E., Nierman, W. C. & Chamberlin, M. J. A direct effect of guanosine tetraphosphate on pausing of Escherichia coli RNA polymerase during RNA chain elongation. *J. Biol. Chem.* **256**, 2787–2797 (1981). 7009598

[43] Tabib-Salazar, A. & Wigneshweraraj, S. RNA Management during T7 infection. *Phage* **3**, 136–140 (2022). 36793551 10.1089/phage.2022.0029 PMC9917321

[44] Rodnina, M. V. Translation in Prokaryotes.* Cold Spring Harb. Perspect. Biol.*** 10**, 10.1101/cshperspect.a032664 (2018). 10.1101/cshperspect.a032664 PMC6120702 29661790

[45] Casjens, S. R. The DNA-packaging nanomotor of tailed bacteriophages. *Nat. Rev. Microbiol.* **9**, 647–657 (2011). 21836625 10.1038/nrmicro2632

[46] Lewanczyk, A. C. et al. The opposite effects of stringent response on phage infection of Pseudomonas putida. *Microlife* **7**, uqaf048 (2026). 41561391 10.1093/femsml/uqaf048 PMC12814882

[47] Stano, N. M. & Patel, S. S. T7 lysozyme represses T7 RNA polymerase transcription by destabilizing the open complex during initiation. *J. Biol. Chem.* **279**, 16136–16143 (2004). 14764584 10.1074/jbc.M400139200

[48] Zhang, X. & Studier, F. W. Multiple roles of T7 RNA polymerase and T7 lysozyme during bacteriophage T7 infection. *J. Mol. Biol.* **340**, 707–730 (2004). 15223315 10.1016/j.jmb.2004.05.006

[49] Borges, A. & Athukoralage, J.* Phage amplification and concentration.* https://www.protocols.io/view/phage-amplification-and-concentration-yxmvmnb86g3p/v1/references (2022).

[50] Ceelen, M.* Plaque assay*.https://www.protocols.io/view/plaque-assay-kqdg35ebqv25/v1 (2019).

[51] Kitagawa, M. et al. Complete set of ORF clones of Escherichia coli ASKA library (A Complete Set of E. coli K-12 ORF Archive): Unique Resources for Biological Research. *DNA Res.* **12**, 291–299 (2006). 10.1093/dnares/dsi012 16769691

[52] Baba, T. et al. Construction of Escherichia coli K-12 in-frame, single-gene knockout mutants: the Keio collection. *Mol. Syst. Biol.* **2**, 2006.0008 (2006). 16738554 10.1038/msb4100050 PMC1681482

[53] Thomason, L. C., Costantino, N. & Court, D. L. E. coli genome manipulation by P1 transduction. *Curr. Protoc. Mol. Biol.* **Chapter 1**, 1 17 11–11 17 18 (2007). 10.1002/0471142727.mb0117s79 18265391

[54] Cherepanov, P. P. & Wackernagel, W. Gene disruption in Escherichia coli: TcR and KmR cassettes with the option of Flp-catalyzed excision of the antibiotic-resistance determinant. *Gene* **158**, 9–14 (1995). 7789817 10.1016/0378-1119(95)00193-a

[55] Varik, V., Oliveira, S. R. A., Hauryliuk, V. & Tenson, T. HPLC-based quantification of bacterial housekeeping nucleotides and alarmone messengers ppGpp and pppGpp. *Sci. Rep.* **7**, 11022 (2017). 28887466 10.1038/s41598-017-10988-6 PMC5591245

[56] Nozaki, S. & Niki, H. Exonuclease III (XthA) Enforces In Vivo DNA Cloning of Escherichia coli To Create Cohesive Ends.* J. Bacteriol* **201**, 10.1128/jb.00660-18 (2019). 10.1128/JB.00660-18 PMC6379578 30530516

[57] Liu, Y. et al. Basal ppGpp regulation by SpoT coordinates metabolic homeostasis and acid resistance. Preprint at 10.64898/2026.01.26.700336 (2026).

[58] Zhang, Y., Zborníková, E., Rejman, D. & Gerdes, K. Novel (p)ppGpp Binding and metabolizing proteins of Escherichia coli.* mBio*** 9**, 10.1128/mBio.02188-17 (2018). 10.1128/mBio.02188-17 PMC5845004 29511080

[59] Abramson, J. et al. Accurate structure prediction of biomolecular interactions with AlphaFold 3. *Nature* **630**, 493–500 (2024). 38718835 10.1038/s41586-024-07487-w PMC11168924

[60] Elfmann, C. & Stulke, J. PAE viewer: a webserver for the interactive visualization of the predicted aligned error for multimer structure predictions and crosslinks. *Nucleic Acids Res.* **51**, W404–W410 (2023). 37140053 10.1093/nar/gkad350 PMC10320053

