# Supplementary material for: Phage portal proteins counteract stringent-response–mediated restriction
Source: Nat Commun. 2026 Jul 11;17:8557. doi: 10.1038/s41467-026-75532-5 (PMC13483052; doi:10.1038/s41467-026-75532-5)
Supplement: Supplementary file 1 [file 41467_2026_75532_MOESM1_ESM.pdf]

Phage portal proteins counteract stringent-response–mediated restriction

Table of contents

Supplementary Fig. 1 3
Supplementary Fig. 2 5
Supplementary Fig. 3 6
Supplementary Fig. 4 7
Supplementary Fig. 5 8
Supplementary Fig. 6 9
Supplementary Table 1: PyMOL-rendered pLDDT confidence views and PAE matrices for AlphaFold-predicted phage portal protein models. 10
Supplementary table 2. Plaque formation of wt MG1655 or Δ*relA*Δ*spoT* by 16 different coliphages out of the BASEL collection 15
Supplementary table 3 All strains used in this study 20
Supplementary table 4 All primers used in this study 25

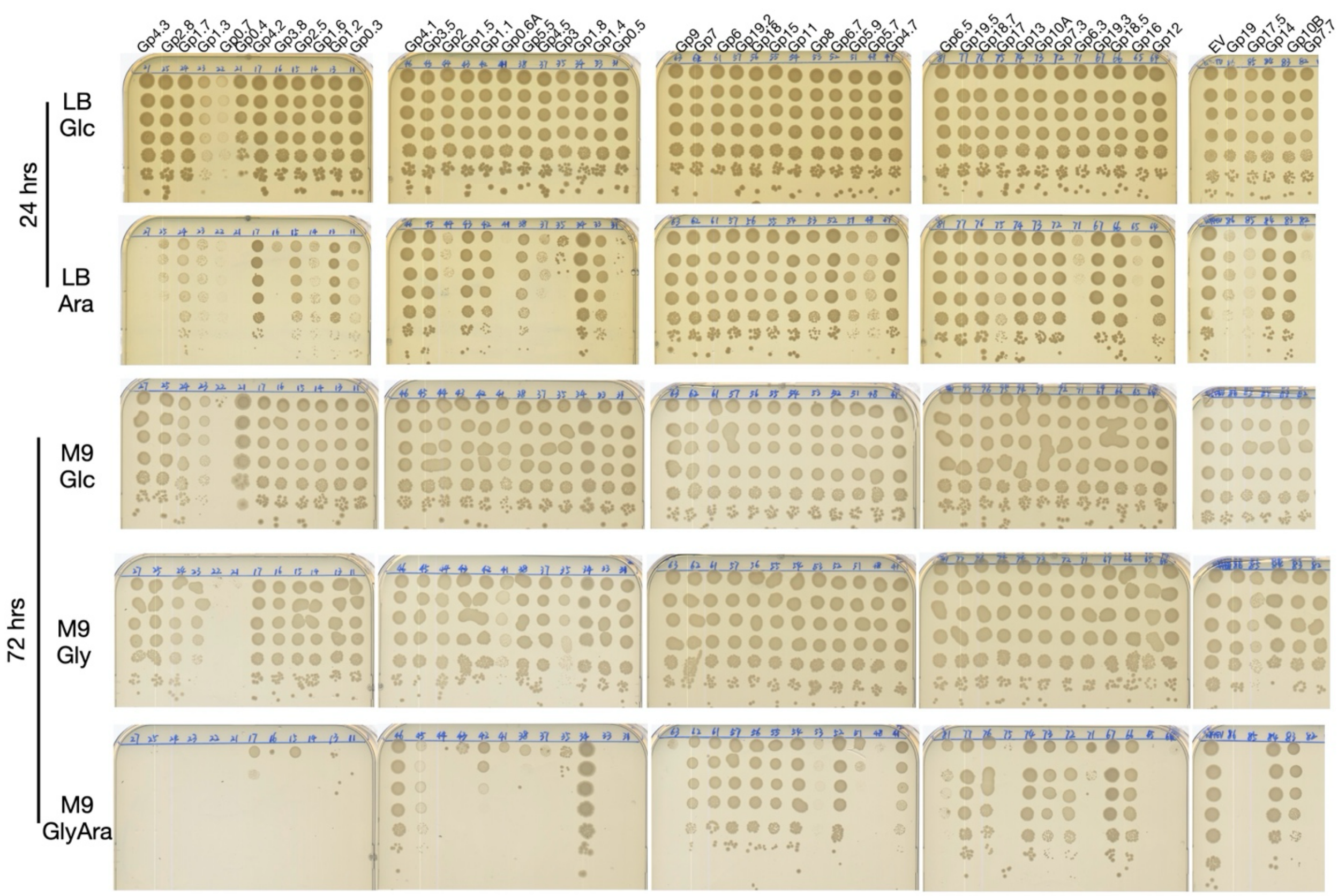

Supplementary Fig. 1

Conditional toxicity of T7 proteins under nutrient limitation.

Serial dilution assays showing growth of *Escherichia coli* expressing individual T7 genes in rich and minimal media. Colony-forming units were normalized to OD_{600nm}. Expression of multiple T7 proteins resulted in growth inhibition specifically under minimal-medium conditions, whereas little or no effect was observed in rich medium. LB Glc and LB Ara are LB media supplemented with 0.2% glucose and 0.2% arabinose, respectively. M9 Glc, M9 Gly, and M9 Gly Ara denote M9 minimal media supplemented with 0.4% glucose, 0.4% glycerol, or 0.4% glycerol plus 0.2% arabinose, respectively. LB plates were imaged after 24 h, and M9 plates after 72 h.

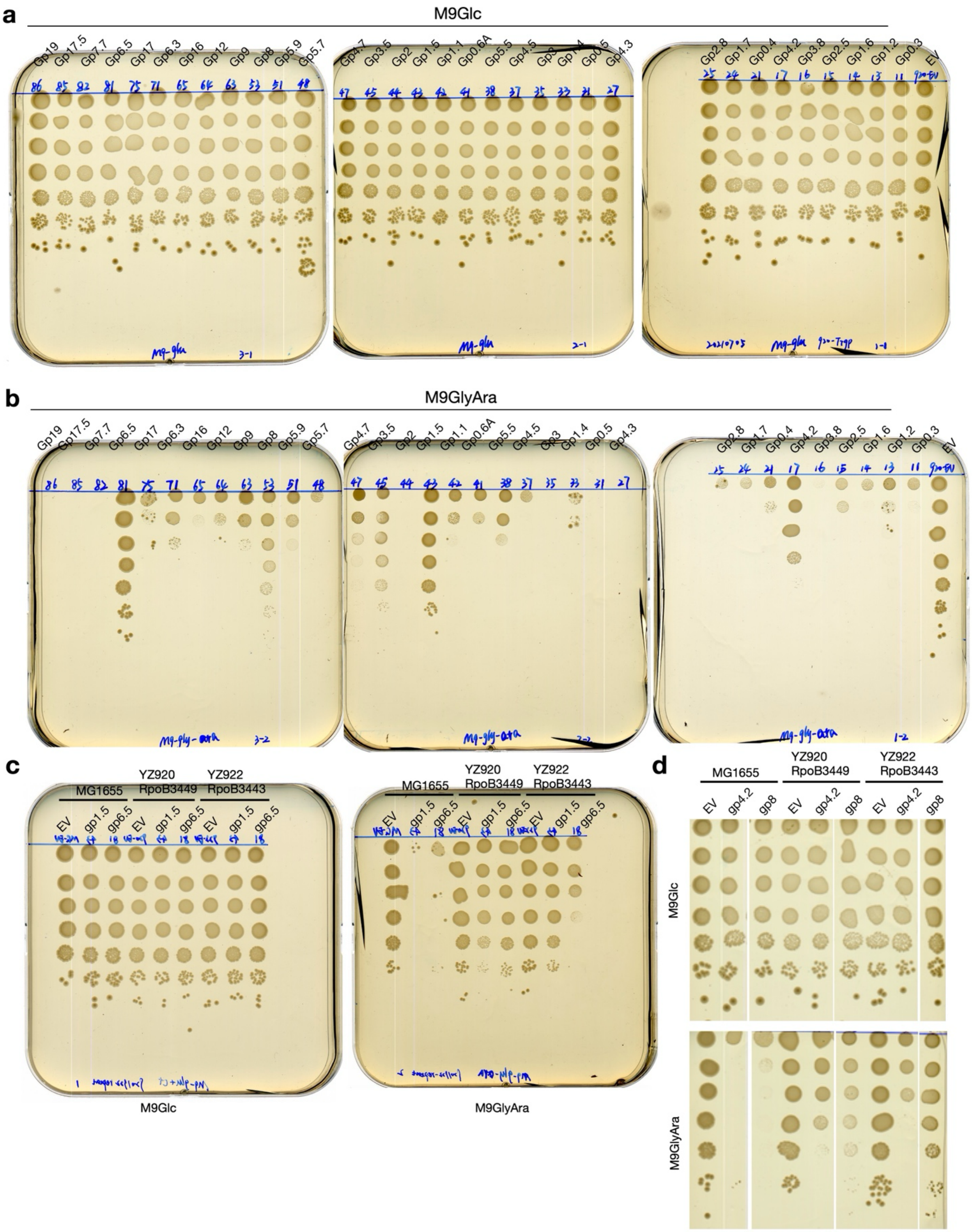
a
M9Glc
Gp19 Gp17.5 Gp7.7 Gp6.5 Gp17 Gp6.3 Gp16 Gp12 Gp9 Gp8 Gp5.9 Gp5.7
Gp4.7 Gp3.5 Gp2 Gp1.5 Gp1.1 Gp0.6A Gp5.5 Gp4.5 Gp3 Gp1.4 Gp0.5 Gp4.3
Gp2.8 Gp1.7 Gp0.4 Gp4.2 Gp3.8 Gp2.5 Gp1.6 Gp1.2 Gp0.3 EV
b
M9GlyAra
Gp19 Gp17.5 Gp7.7 Gp6.5 Gp17 Gp6.3 Gp16 Gp12 Gp9 Gp8 Gp5.9 Gp5.7
Gp4.7 Gp3.5 Gp2 Gp1.5 Gp1.1 Gp0.6A Gp5.5 Gp4.5 Gp3 Gp1.4 Gp0.5 Gp4.3
Gp2.8 Gp1.7 Gp0.4 Gp4.2 Gp3.8 Gp2.5 Gp1.6 Gp1.2 Gp0.3 EV
c
MG1655
YZ920 RpoB3449
YZ922 RpoB3443
EV gp1.5 gp6.5 EV gp1.5 gp6.5 EV gp1.5 gp6.5
M9Glc
M9GlyAra
d
MG1655
YZ920 RpoB3449
YZ922 RpoB3443
EV gp4.2 gp8 EV gp4.2 gp8 EV gp4.2 gp8
M9Glc
M9GlyAra

Supplementary Fig. 2

Suppression of T7 protein-induced growth defects by stringent-like RNA polymerase alleles. **a,b**, The YZ920 strain carrying the stringent-like *rpoB*3449 mutation was transformed with pBAD33 constructs expressing T7 proteins that were toxic in the wild-type *E. coli* MG1655 strain. Transformants were tested on M9 medium supplemented with glucose (M9Glc; **a**) or glycerol plus arabinose (M9GlyAra; **b**) and incubated for 72 h. **c,d**, Ten-fold serial dilutions (top to bottom) of wild-type MG1655 or YZ920 and YZ922 strains carrying the stringent-like *rpoB*3449 and *rpoB*3443 mutations, respectively, were spotted onto M9Glc or M9GlyAra plates. Cells expressed the indicated T7 genes (*gp4.2*, *gp1.5*, *gp8*, *gp6.5*) or an empty vector (EV). Plates were incubated at 37 °C and photographed after 72 h. Representative plates from three independent biological replicates are shown. Source data are provided as a Source Data file.

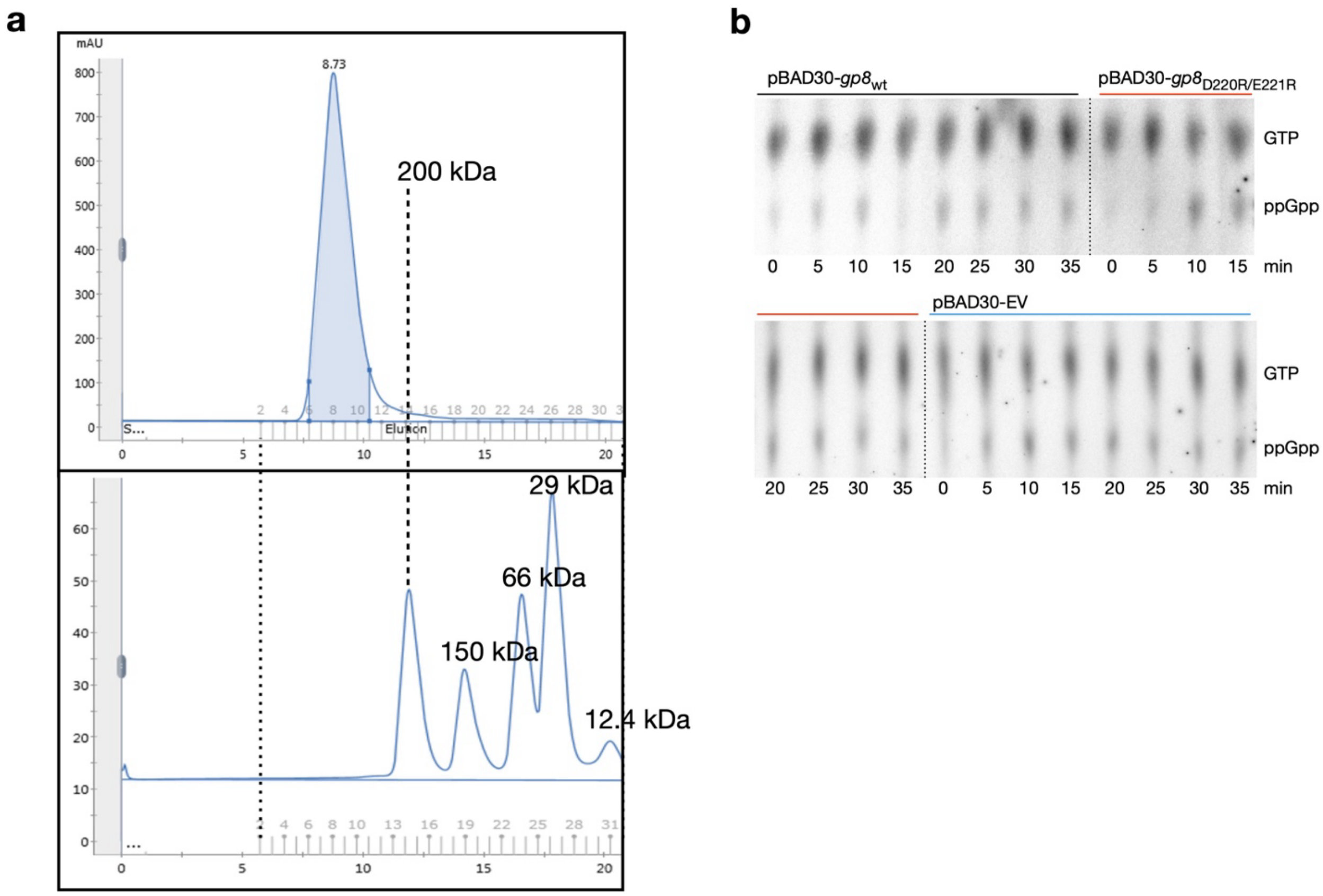

Supplementary Fig. 3

a. Size-exclusion chromatography profile of purified wild-type Gp8-His6. Purified wild-type Gp8-His6 eluted as a single major SEC peak earlier than the 200-kDa molecular-mass standard, indicating a relatively uniform species with an apparent molecular mass >200 kDa. As the Gp8 monomer is approximately 60 kDa, this profile is consistent with formation of a higher-order assembly, although SEC does not allow assignment of a defined oligomeric state. The molecular-mass standard profile is shown below for reference. Representative profile from three independent biological replicates is shown.

b. Gp8 expression reduces intracellular (p)ppGpp accumulation.
Thin-layer chromatography (TLC) analysis of **^{32}P**-labeled nucleotide pools during expression of wild-type Gp8 or the interaction-defective mutant Gp8$_{D220R/E221R}$ from the pBAD30 vector. Gp8 expression was induced with 0.2% arabinose, and 25 µL of each sample was analyzed by TLC (see Materials and Methods for details). Positions of GTP and ppGpp are indicated, along with the time points (minutes) following arabinose induction. Representative plates from two independent biological replicates are shown. Source data are provided as a Source Data file.

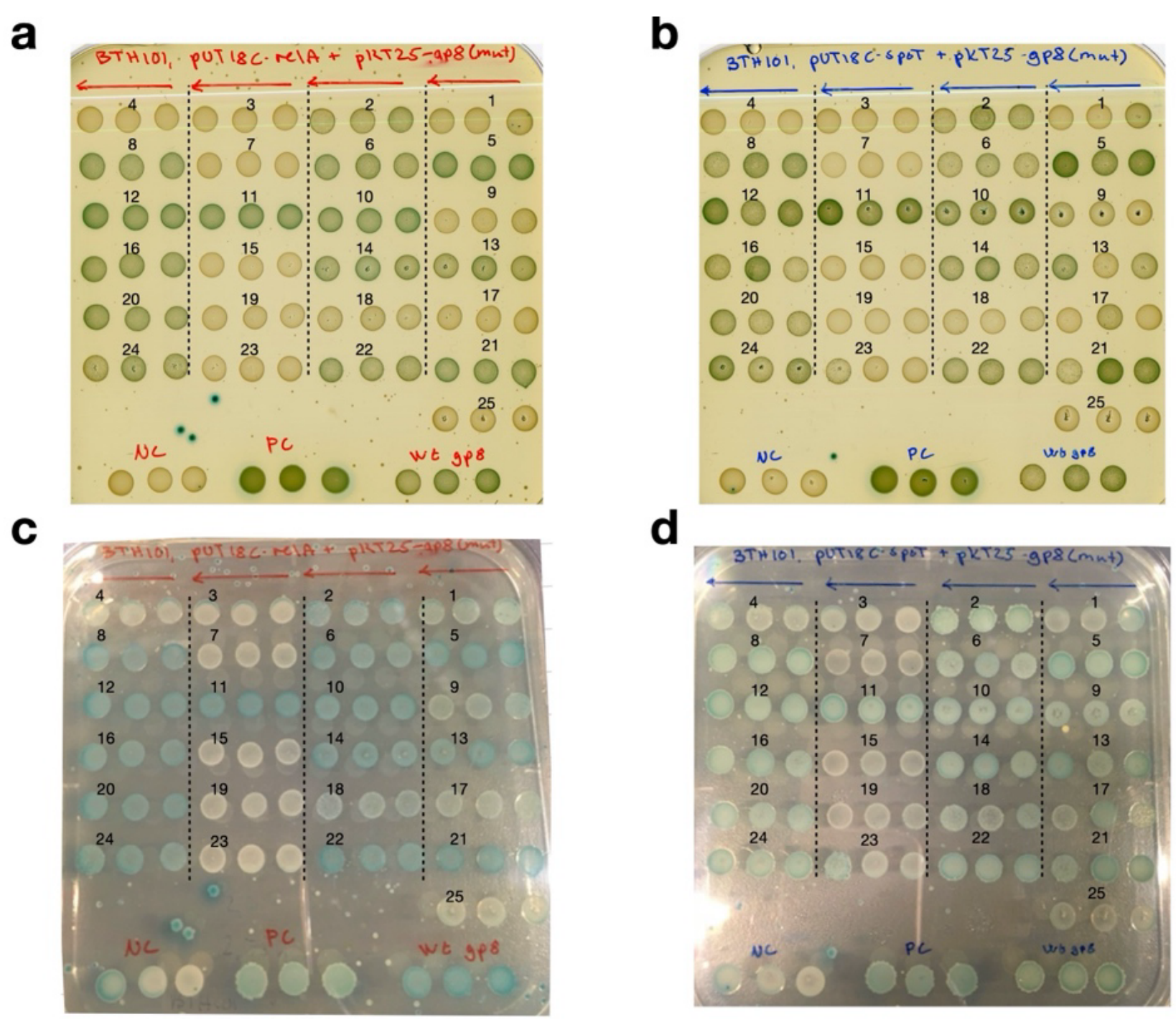

e

1	*D46K/D48K/D53K*
2	D99K/D101K
3	D220R/E221R/D222R/E225R
**4**	**D220R/E221R**
5	D222R/E225R
6	D241K
**7**	**D457K/D460K**
8	D502R
9	*E10K/D11K*
10	E149K/E151K
11	E192R/D193K
12	E199R/E204R
13	E236R
14	E247R
**15**	**E280R/E284R**
16	E416K/E423K
17	*E485K/E486K*
18	*E521R/D528R*
**19**	**K4D/R5D**
20	K94D
21	K105E
22	K196E
**23**	**K288D/K295D**
24	K419D
25	*R277D*

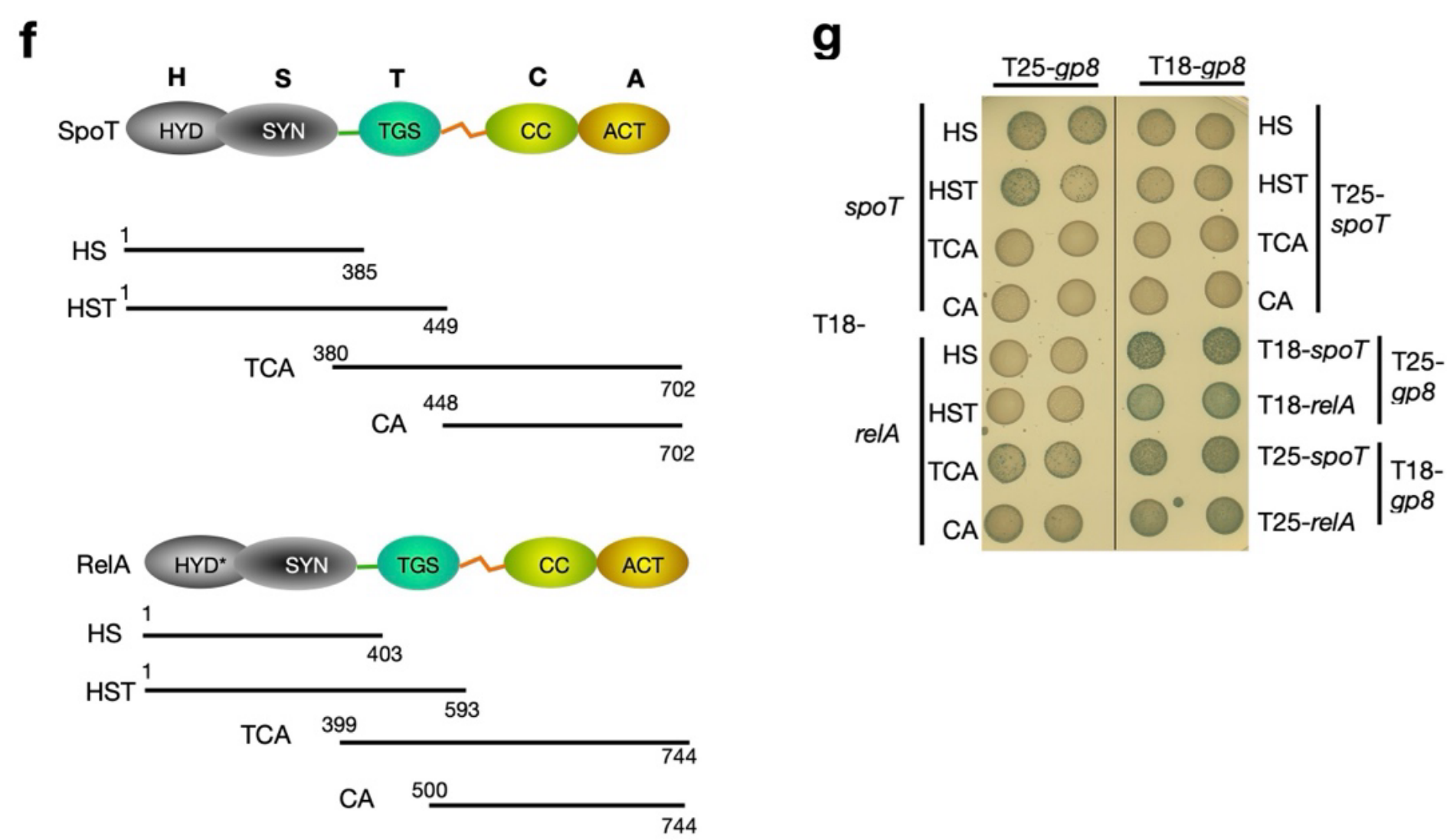

Supplementary Fig. 4

Charge-reversal mutations in Gp8 disrupt interaction with RelA and SpoT. (a-d) Bacterial two-hybrid assays assessing interactions between charge-reversal mutants of Gp8 and RelA **(a, c; 24 h images)** or SpoT **(b, d; 48 h images)**. Independent colonies (n =3) are shown for each mutant. Mutations are numbered as indicated in panel **(e)**. Mutants shown in bold denote strongly defective interactions, whereas mutants shown in italics exhibit partial defects. **(f)**. Schematic overview of RelA and SpoT domain architectures and the truncation constructs tested in panel (**g**). Start and end residue numbers are indicated. (**g**) Bacterial two-hybrid assays assessing

interactions between Gp8 and the RelA/SpoT truncations shown in (**f**). Independent colonies (n = 2) are shown side by side. Source data are provided as a Source Data file.

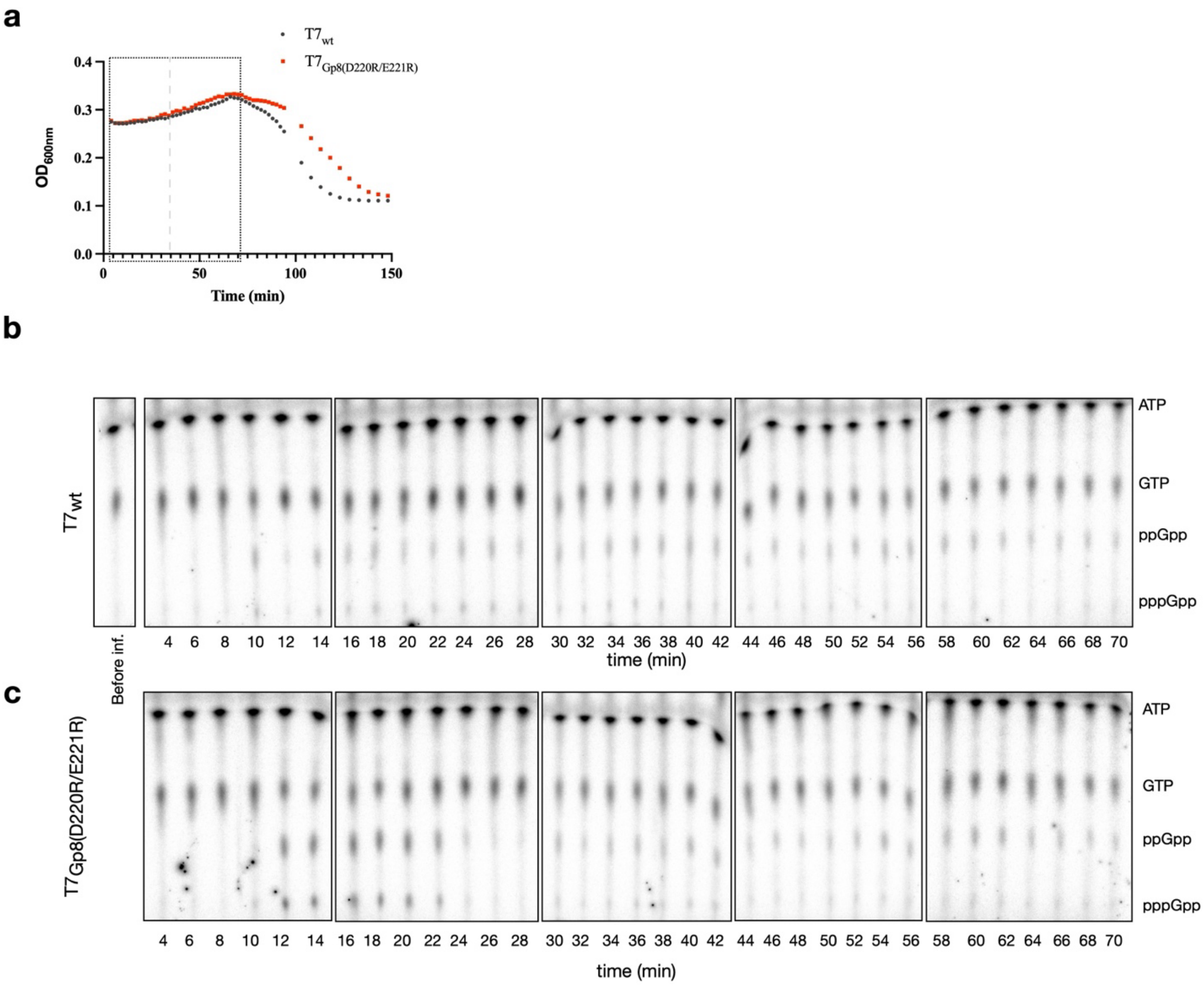

Supplementary Fig. 5

Sustained alarmone accumulation during infection with interaction-defective portal mutants. (a) Lysis kinetics of *E. coli* MG1655 growing in in MOPS minimal medium supplemented with low phosphate (0.2 mM K_2HPO_4), and 0.2% (v/v) glucose, and infected with wild-type T7 or gp8 interaction-defective mutant phages. The boxed region indicates the time window quantified in **(b,c)** and shown in **Fig. 5e**. **(b,c)** Radiolabeled nucleotide analysis of alarmone dynamics during phage infection. Intracellular nucleotide pools were ^{32}P-labeled during infection of *E. coli* MG1655 in MOPS medium with T7 encoding wild-type gp8 **(b)** or interaction-defective gp8 **(c)**. The positions of ATP, GTP, ppGpp, and pppGpp are indicated. Representative plates from two independent biological replicates are shown. Source data are provided as a Source Data file.

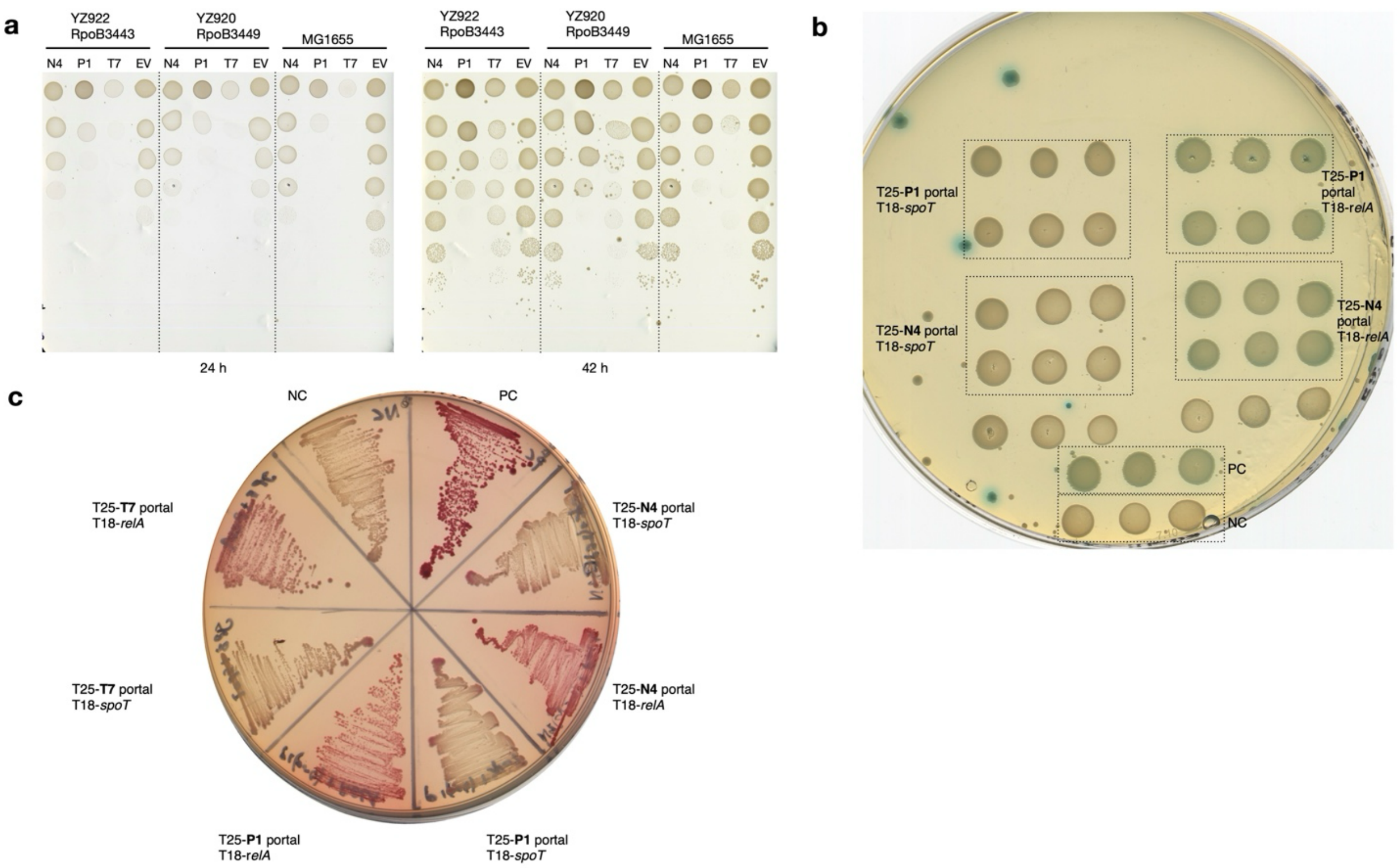

Supplementary Fig. 6

Portal proteins from P1 and N4 exhibit stringent-response-linked phenotypes.

a. Portal proteins from bacteriophages N4 and P1 were cloned into the arabinose-inducible pBAD33 vector and expressed in wild-type *Escherichia coli* MG1655 or in stringent-response-altered strains YZ920 and YZ922, carrying the *rpoB3449* and *rpoB3443* alleles, respectively. Toxicity was assessed on M9 minimal medium supplemented with 0.4% glycerol and 0.2% arabinose. Plates were imaged after 24 and 42 h of incubation at 37 °C. Labels N4, P1, and T7 denote portal proteins from the respective phages; EV indicates the empty-vector control. Representative of the independently replicated (n = 3) plates is shown. **b,c** Bacterial two-hybrid assays assessing interactions between the portal proteins from bacteriophages N4 and P1 and the *E. coli* MG1655 RelA/SpoT proteins, by either LB agar plate with X-gal (**b**) or MacConkey indicator plate (**c**). Independent colonies (n =3 or 6, in dotted frames) are shown side by side in **b**. NC and PC denote negative and positive controls, respectively. Source data are provided as a Source Data file.

Supplementary Table 1: PyMOL-rendered pLDDT confidence views and PAE matrices for AlphaFold-predicted phage portal protein models.

For each AlphaFold-predicted portal protein model, per-residue pLDDT values were visualized in PyMOL using the B-factor putty representation and shown from two orientations. The corresponding predicted aligned error (PAE) matrix is shown in the right column.

<table>
<tr><th>portal protein of phages</th><th>pLDDT/B-factor putty model, view 1</th><th>pLDDT/B-factor putty model, view 2</th><th>PAE matrix</th></tr>
<tr><td>N4</td><td>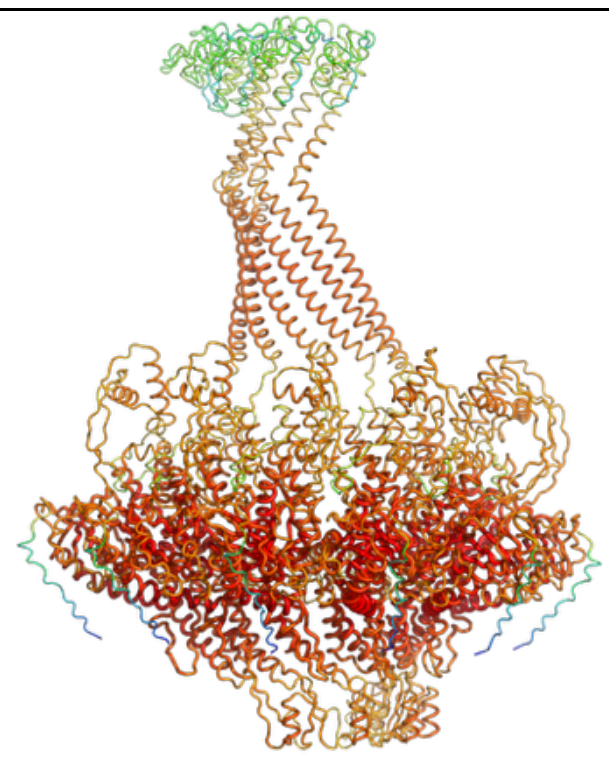</td><td>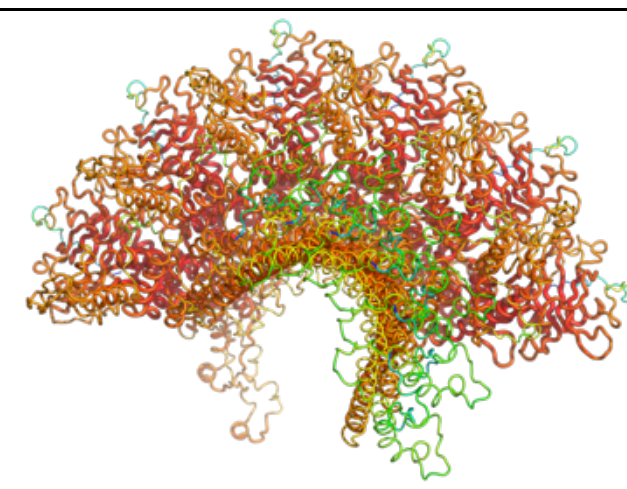</td><td>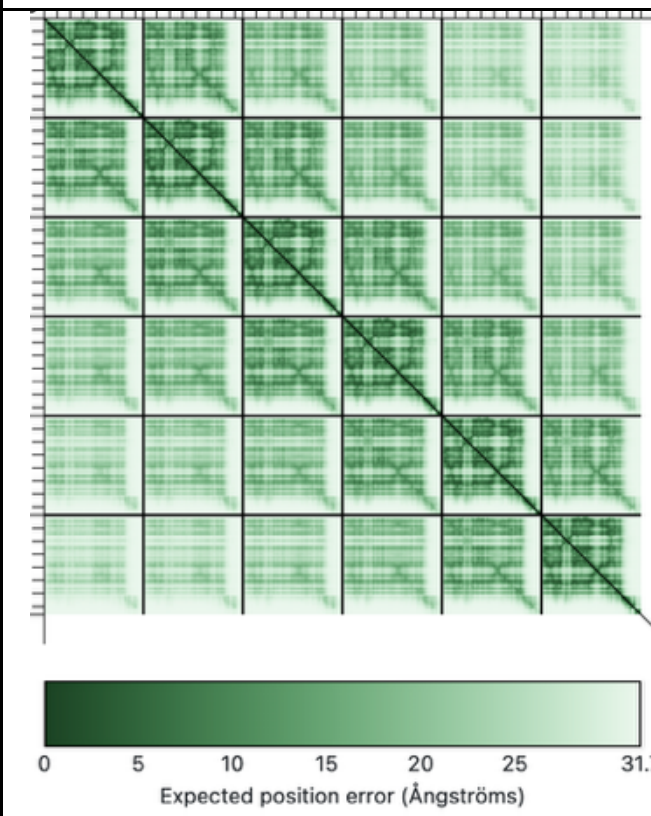
</td></tr>
<tr><td>P1</td><td>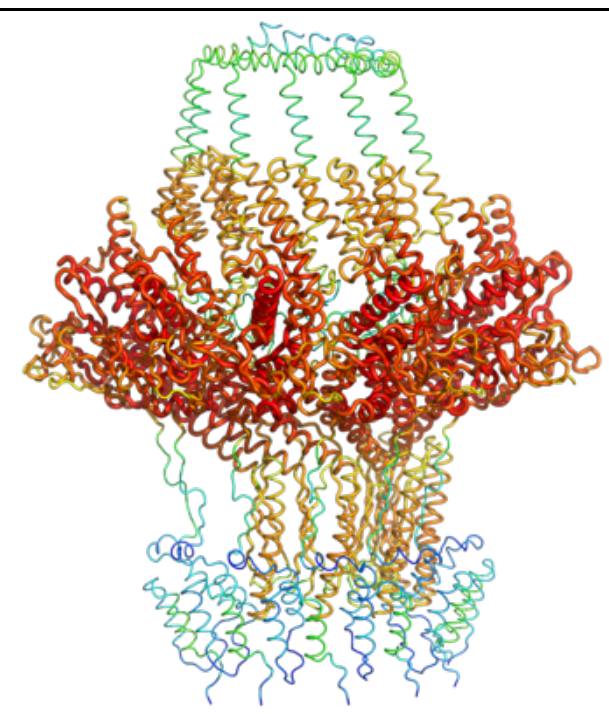</td><td>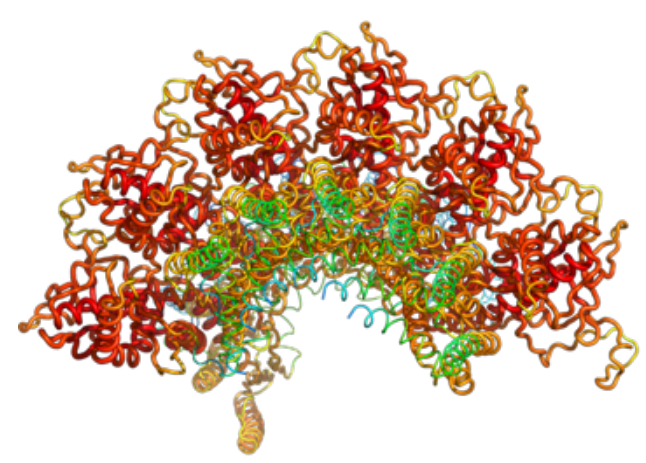</td><td>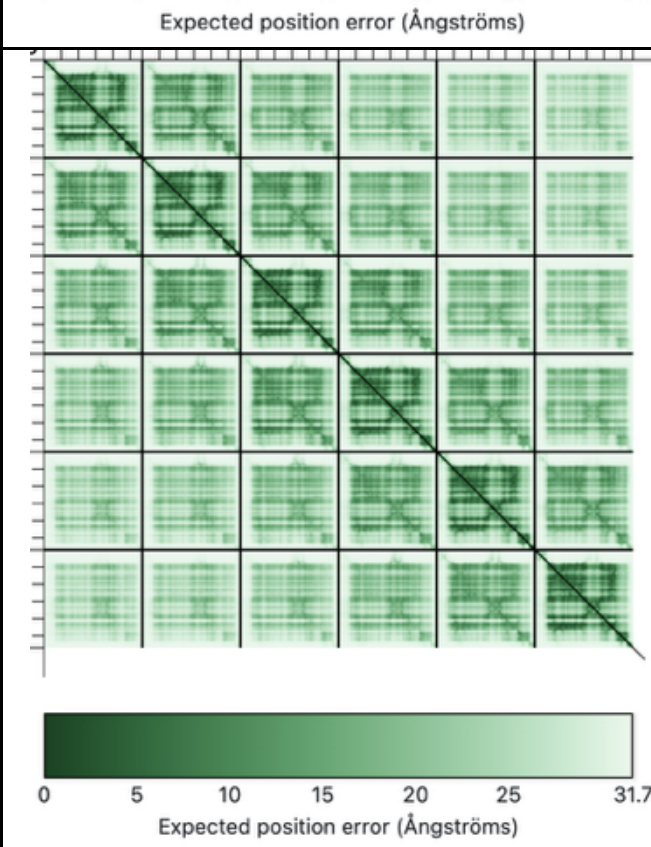
</td></tr>
<tr><td>T2</td><td>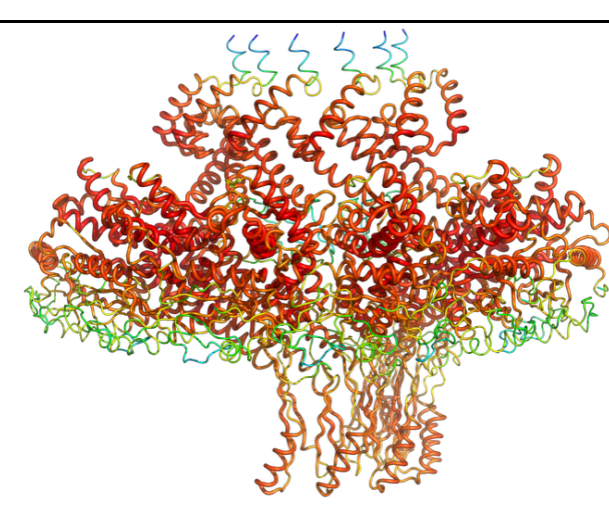</td><td>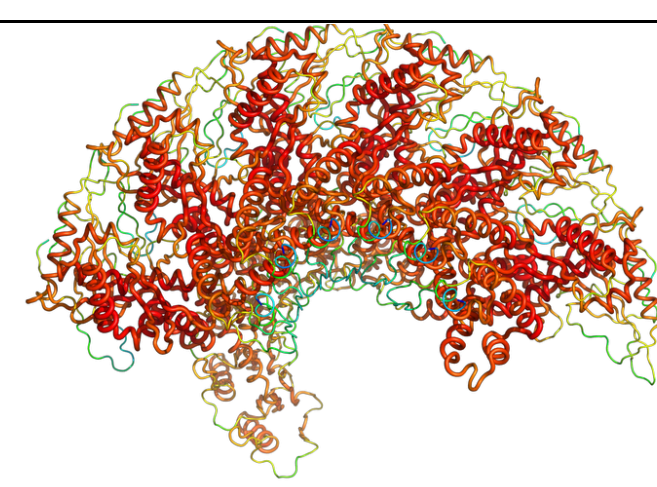</td><td>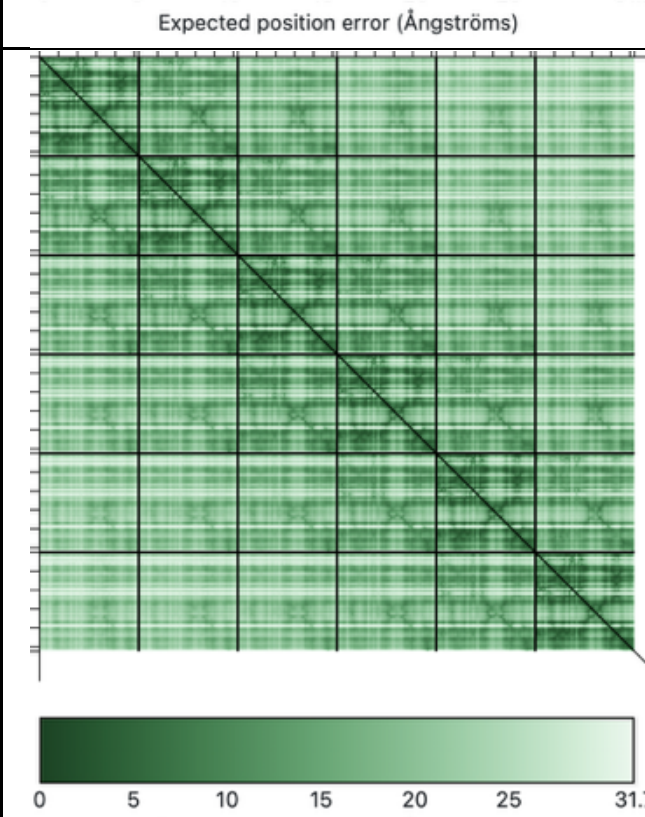
</td></tr>
</table>

Bas02	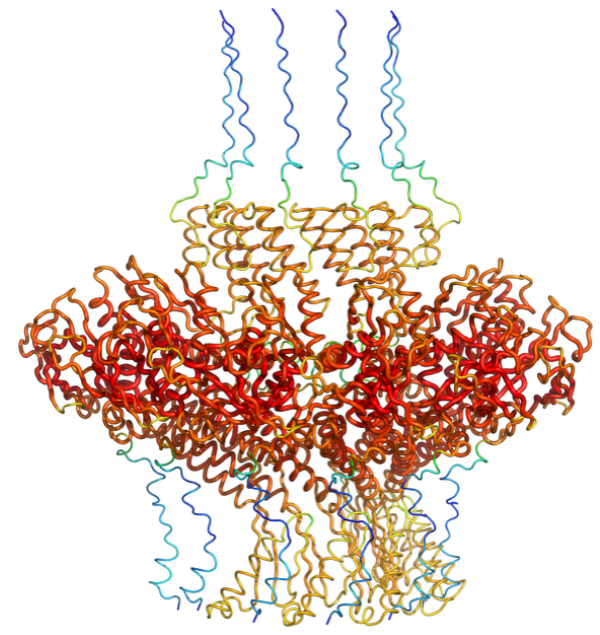	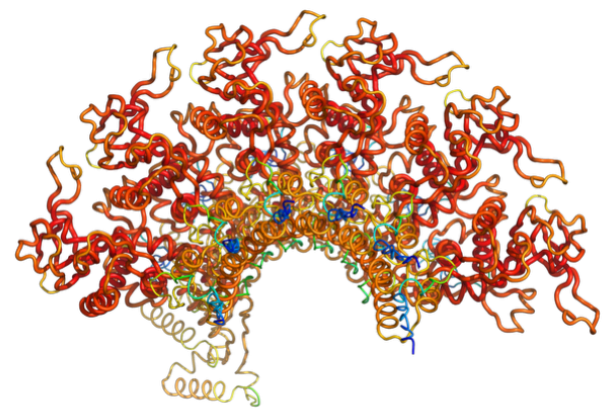	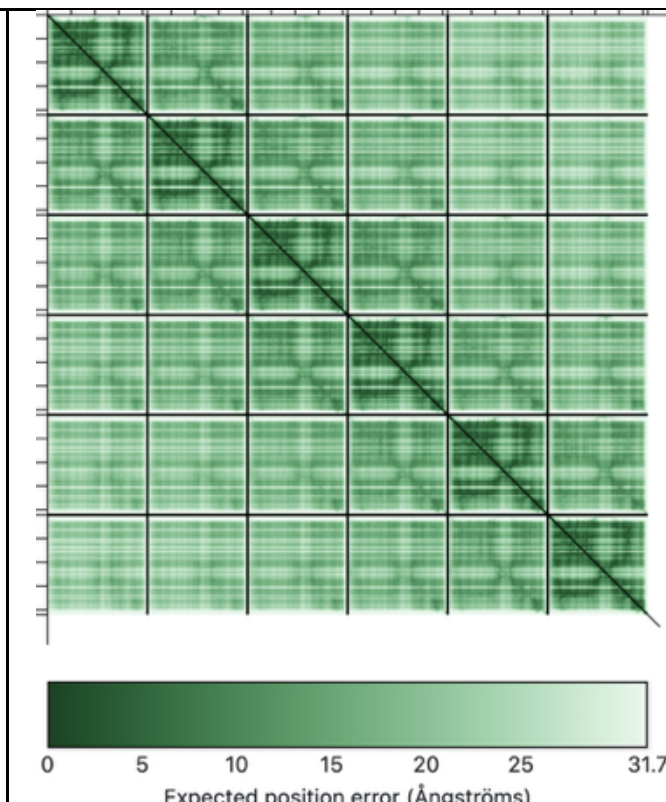
Bas11	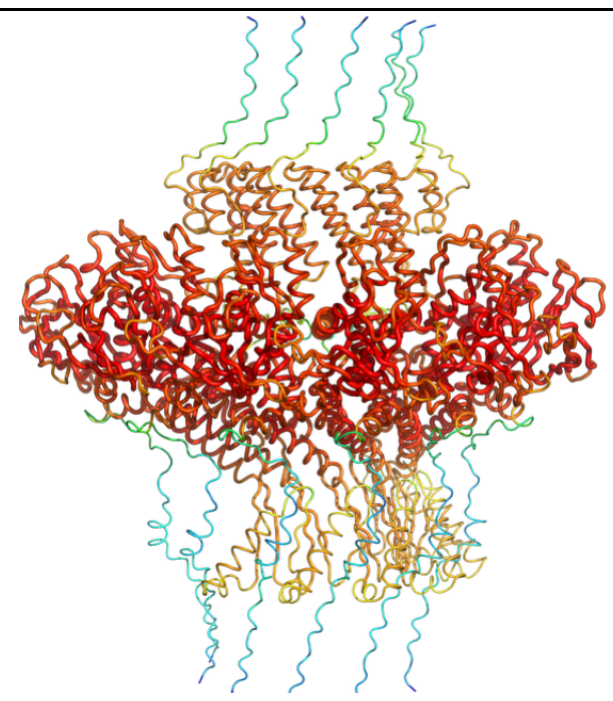	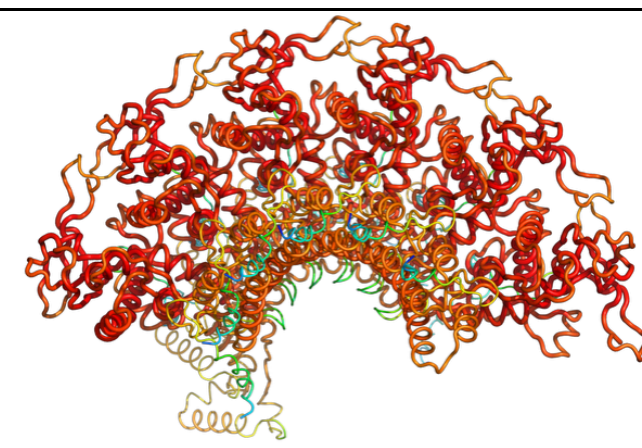	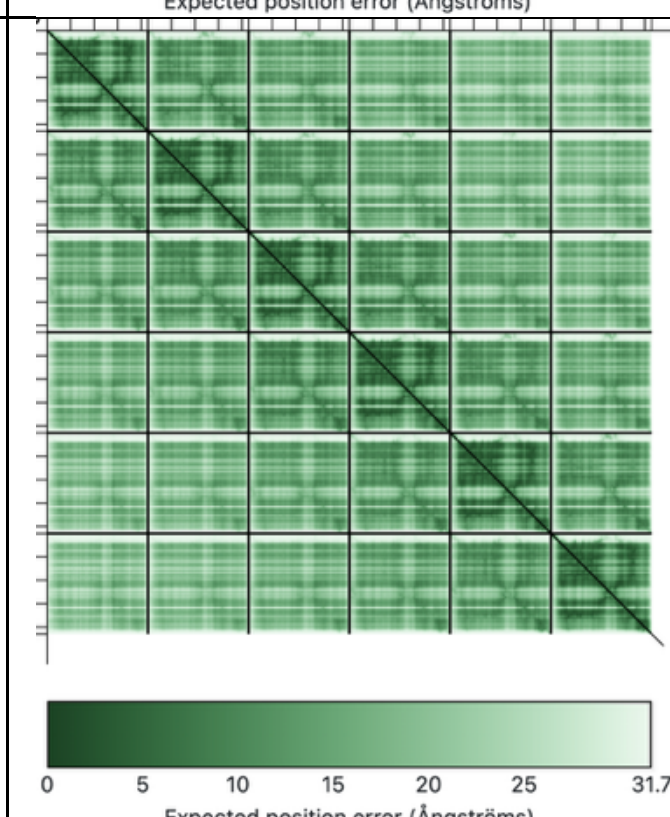
Bas12	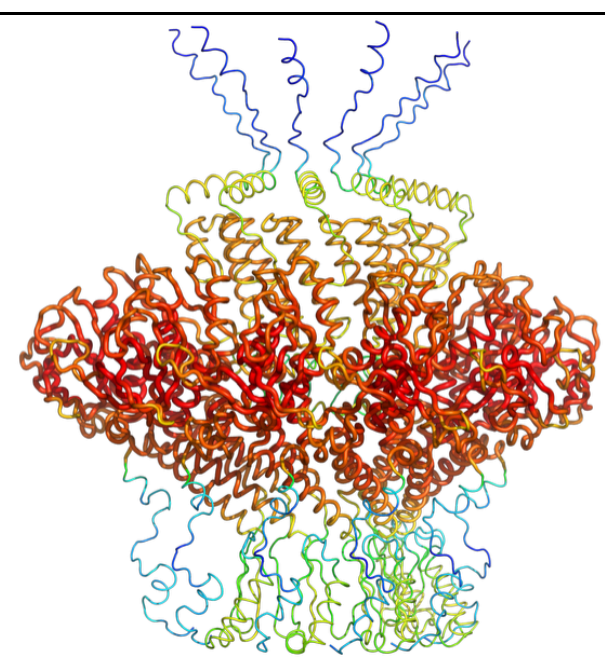	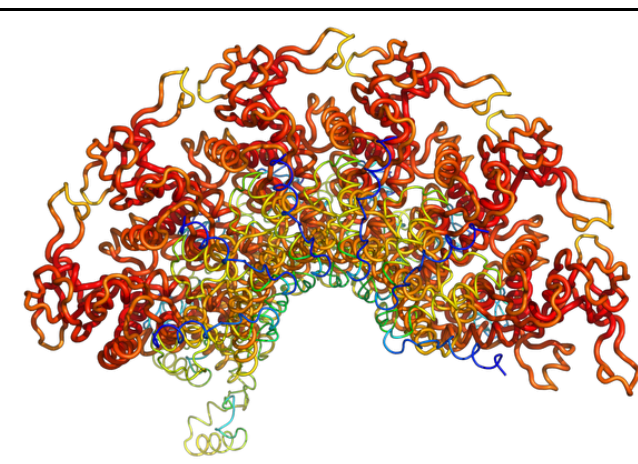	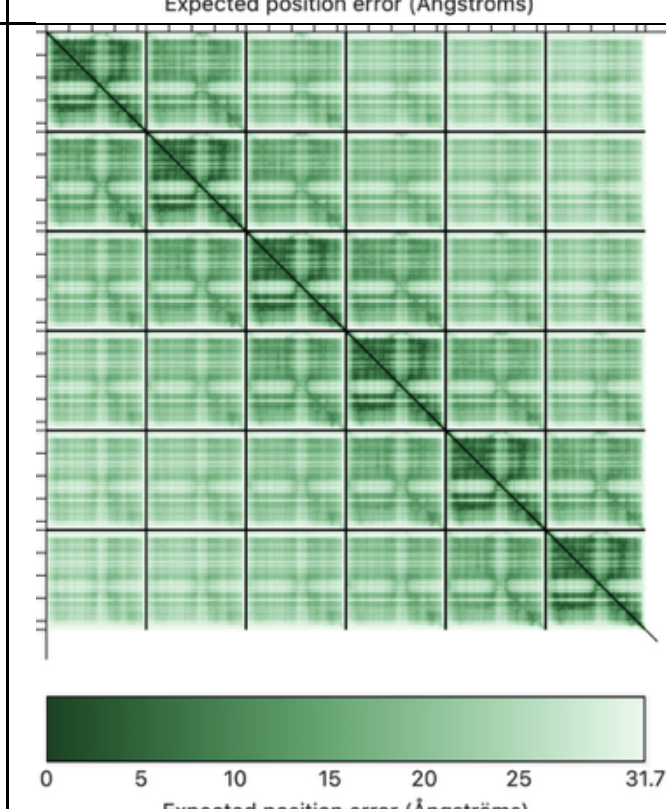
Bas17	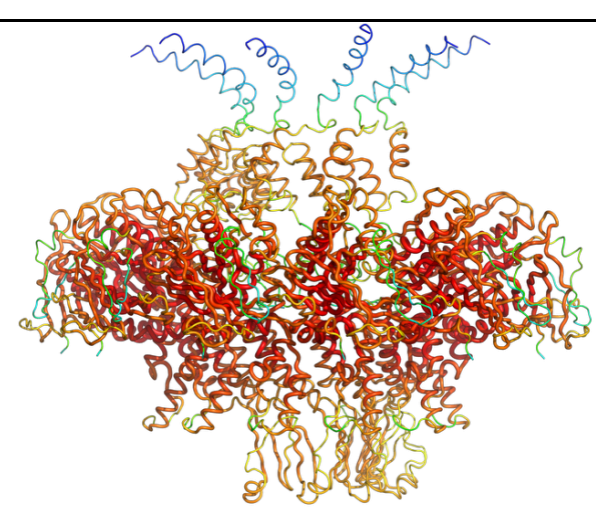	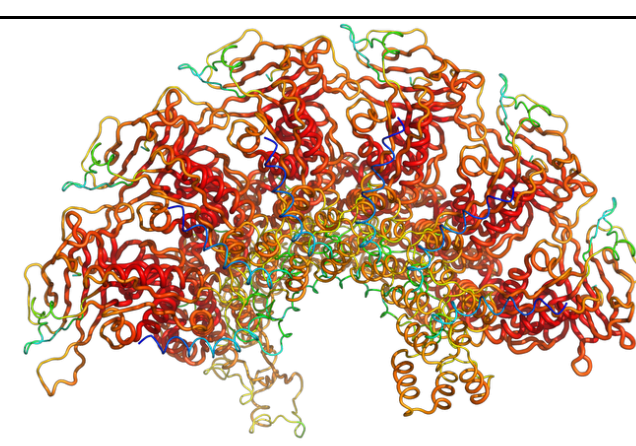	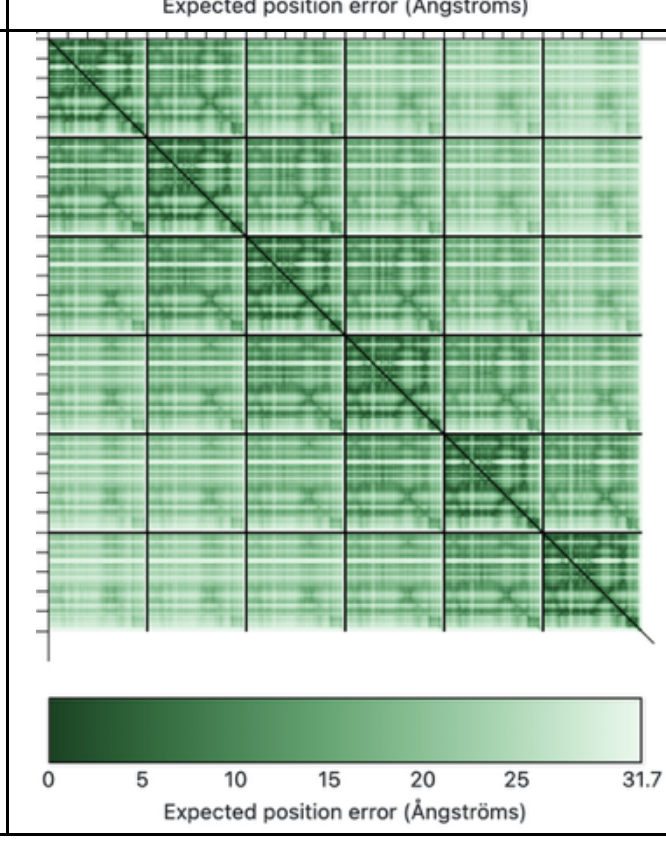

Bas23	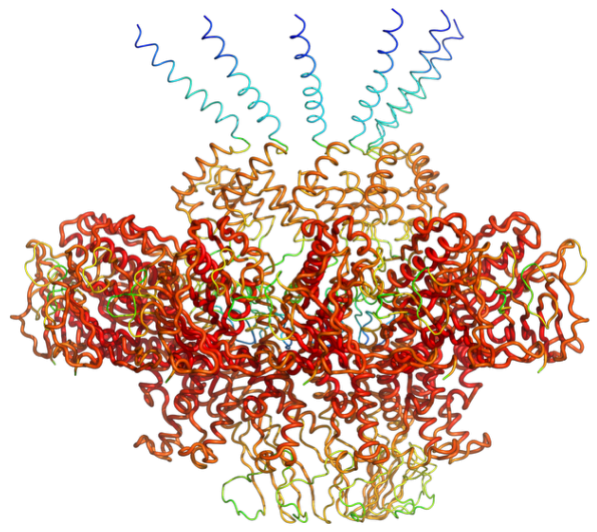	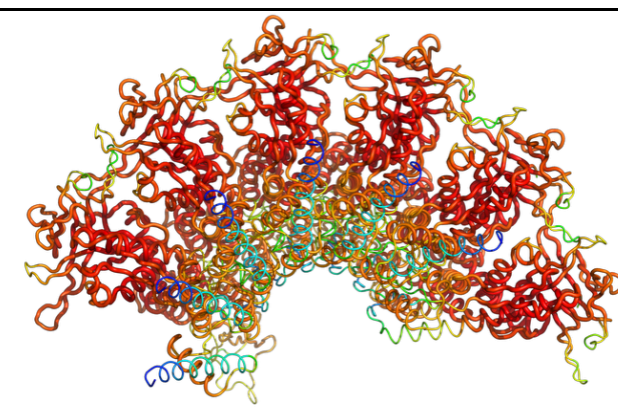	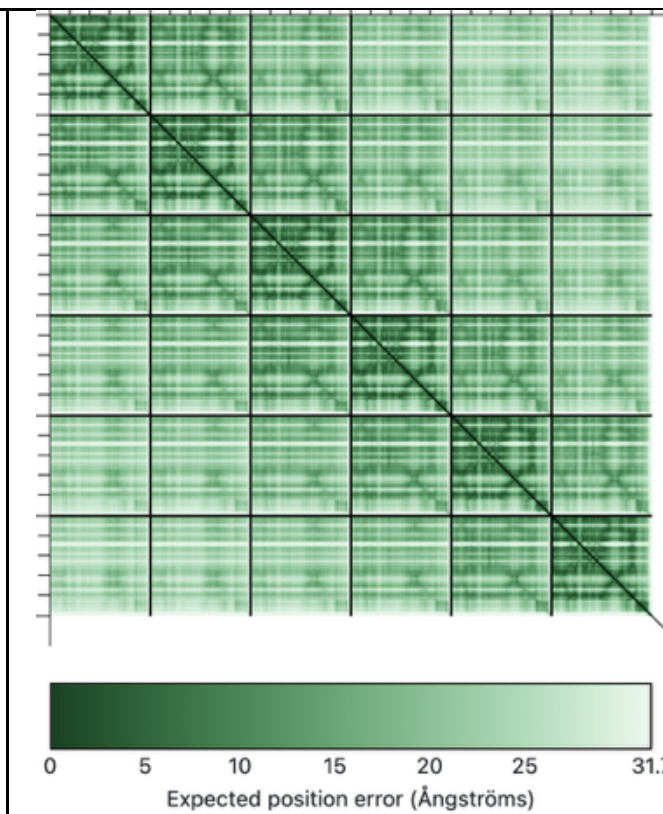
Bas26	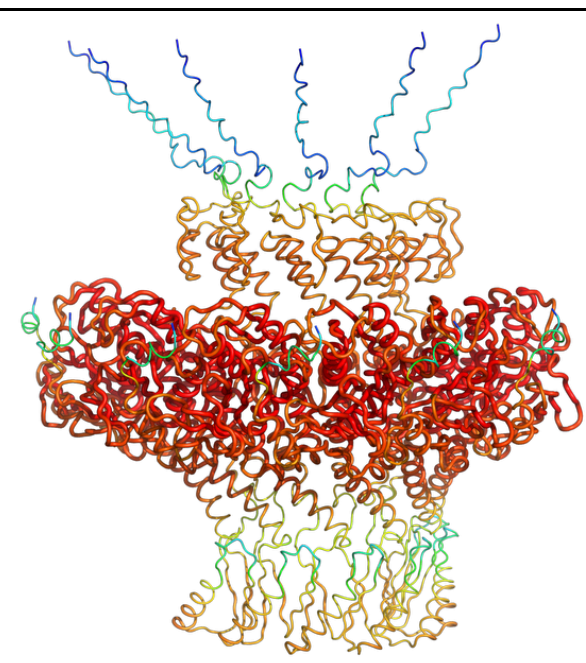	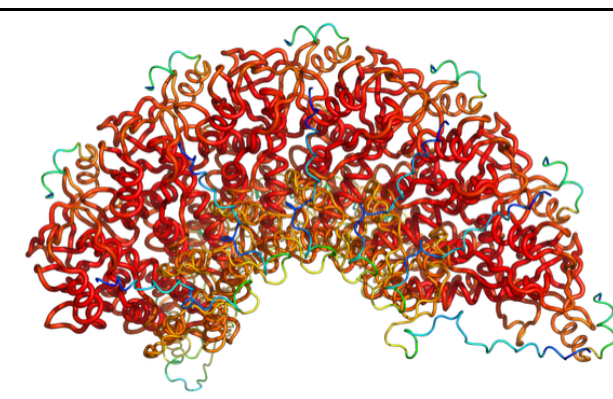	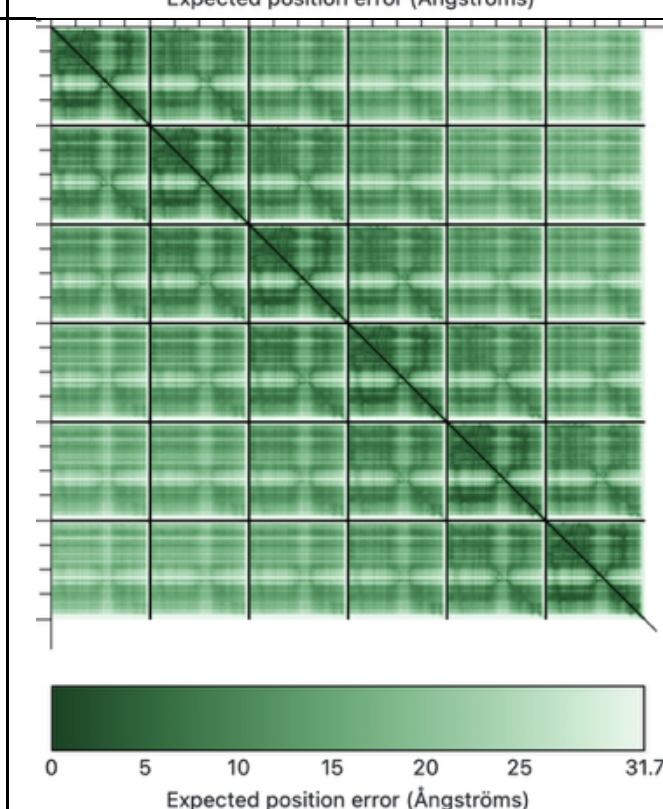
Bas51	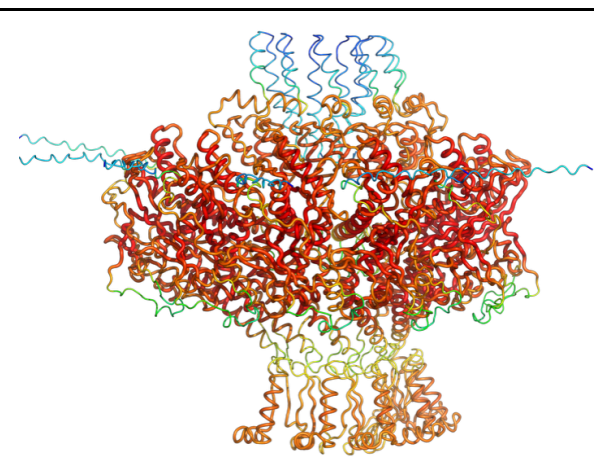	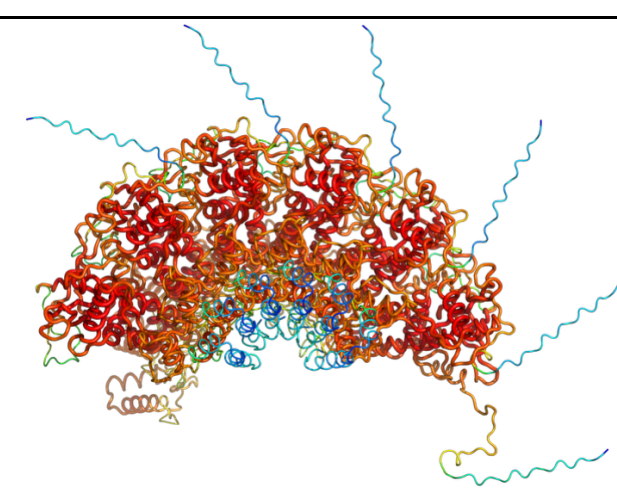	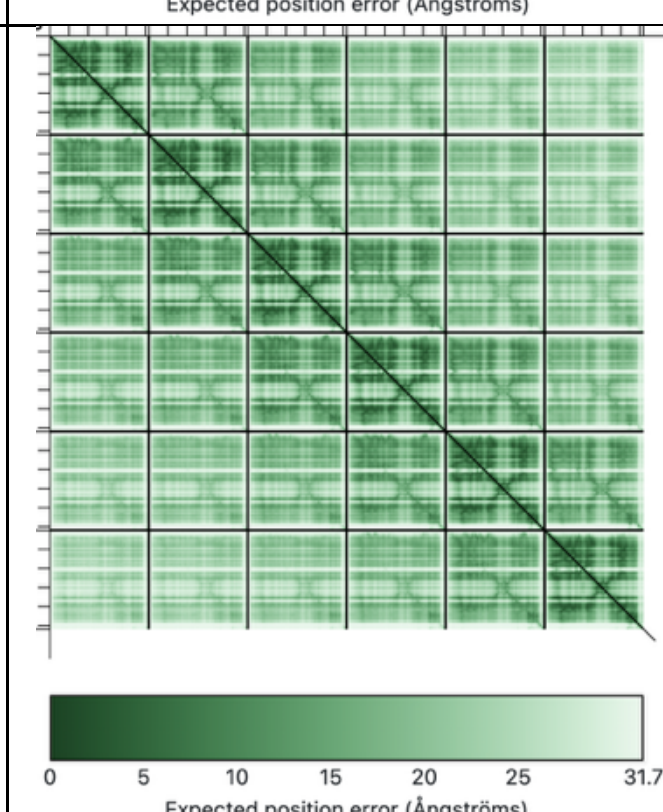
Bas63	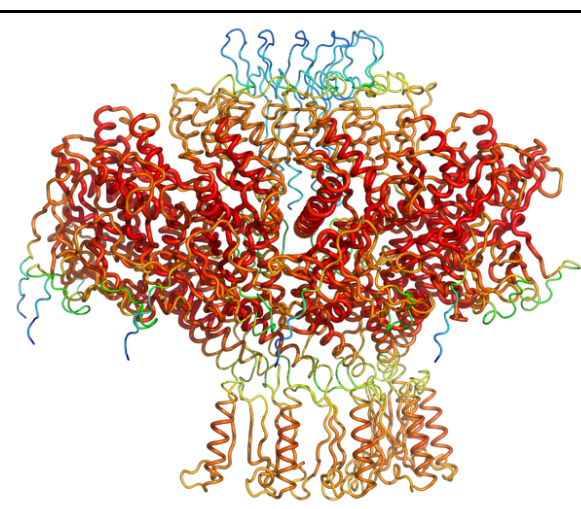	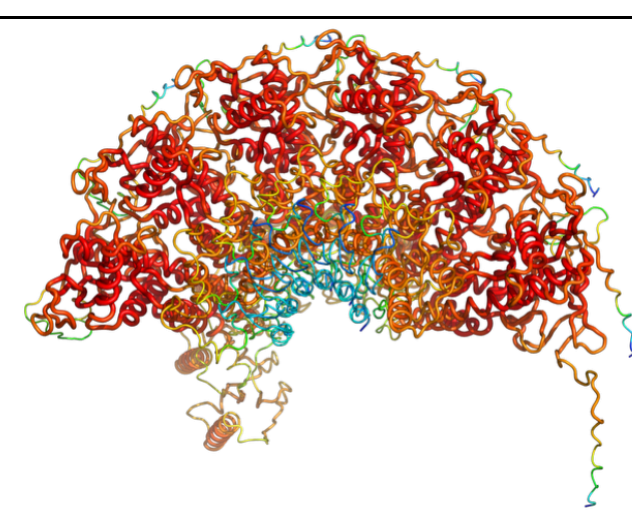	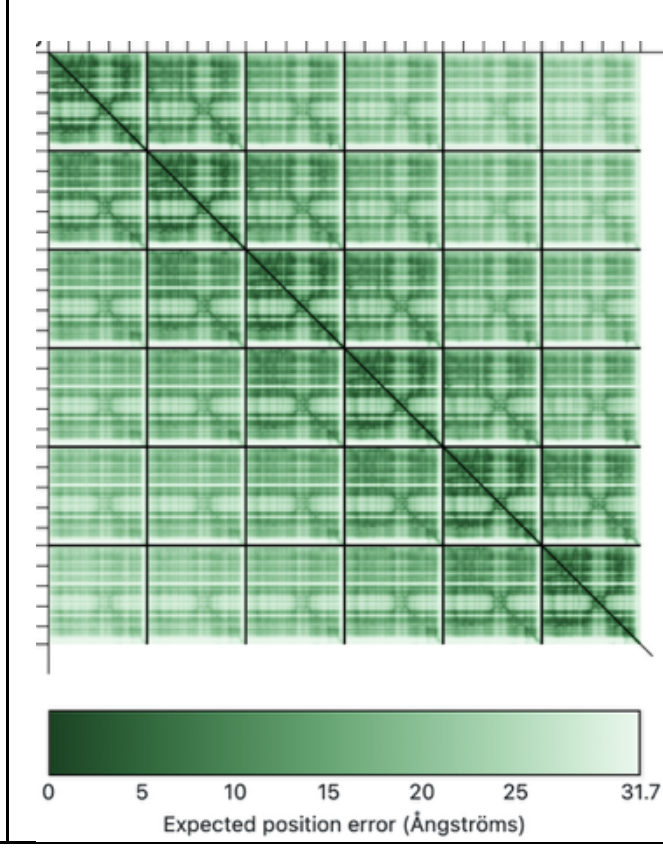

Bas64	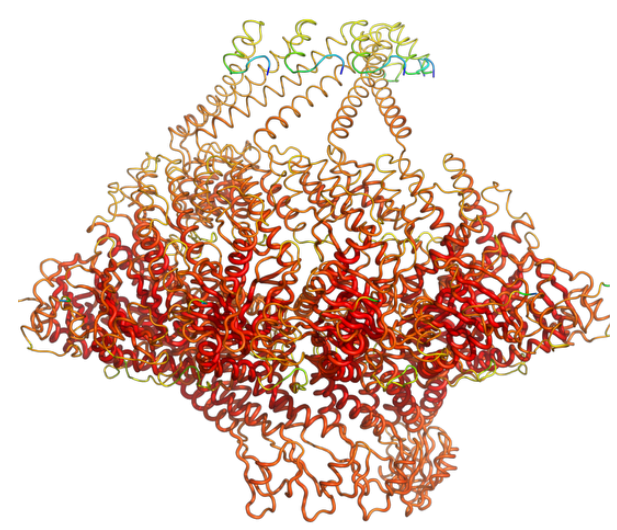	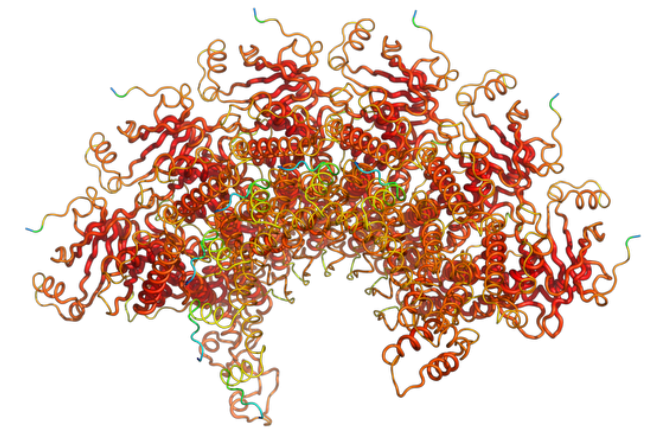	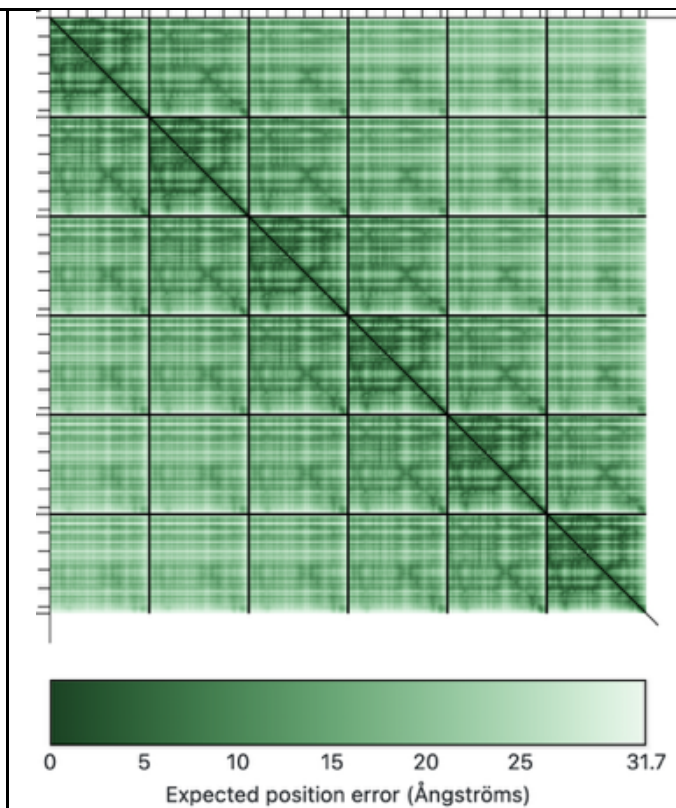
Bas65	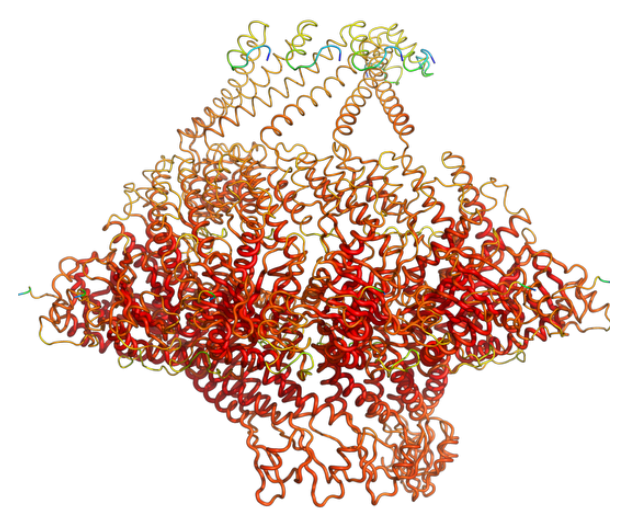	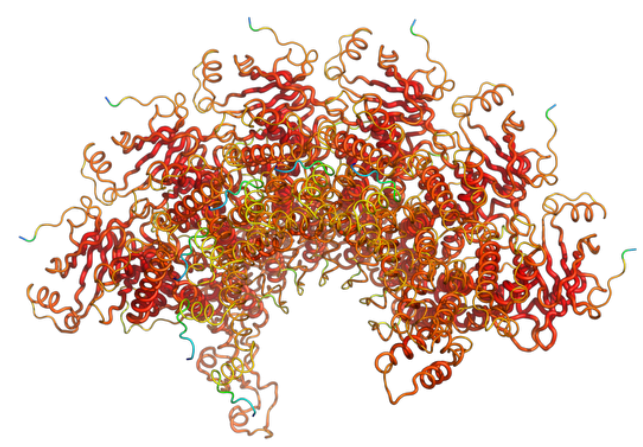	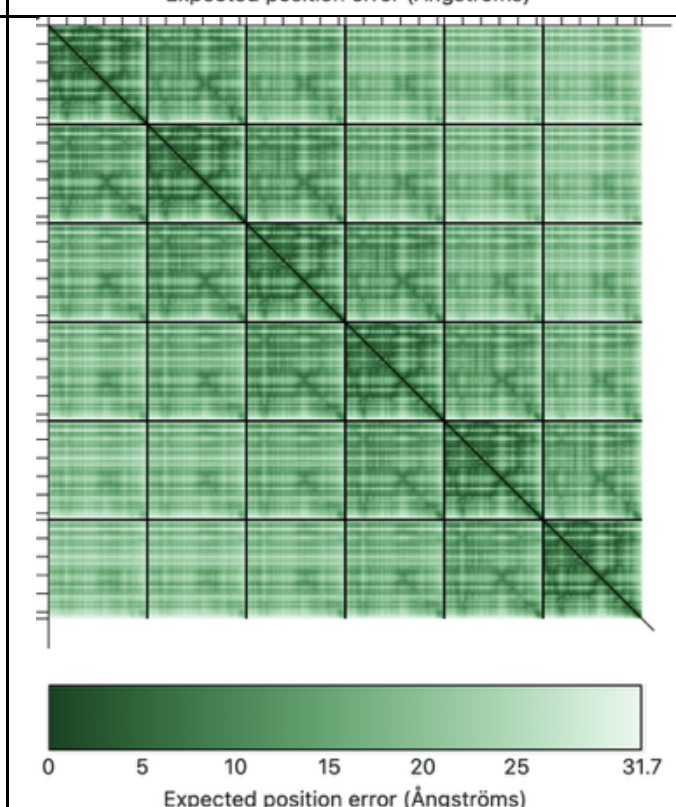
Bas66/<br>T3	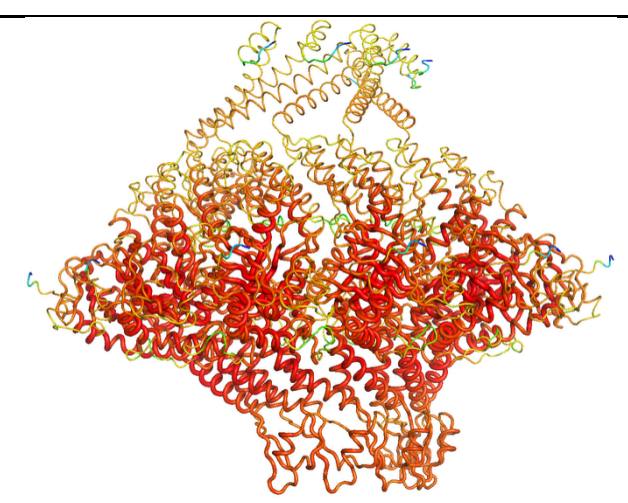	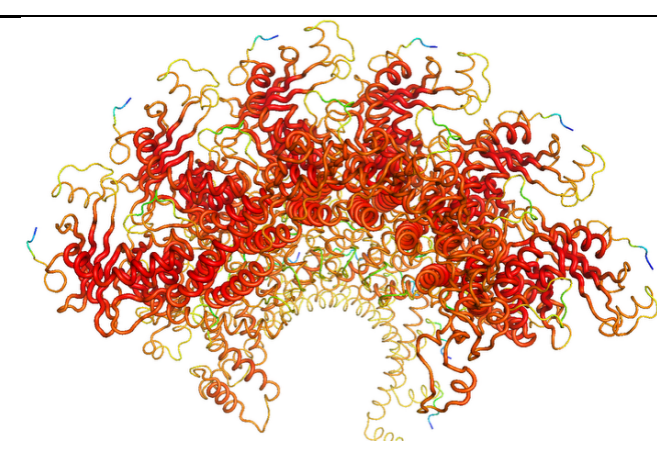	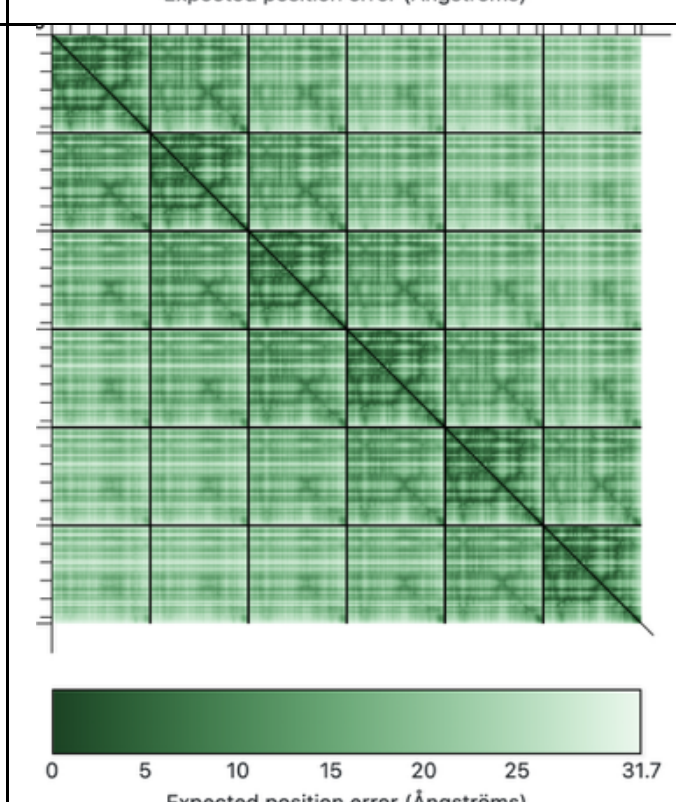
Bas68	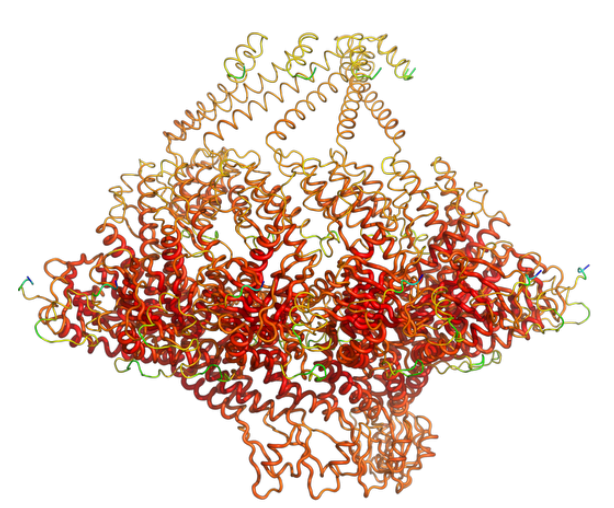	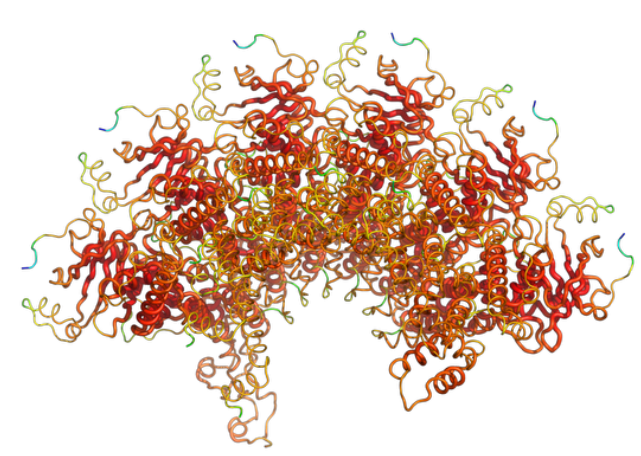	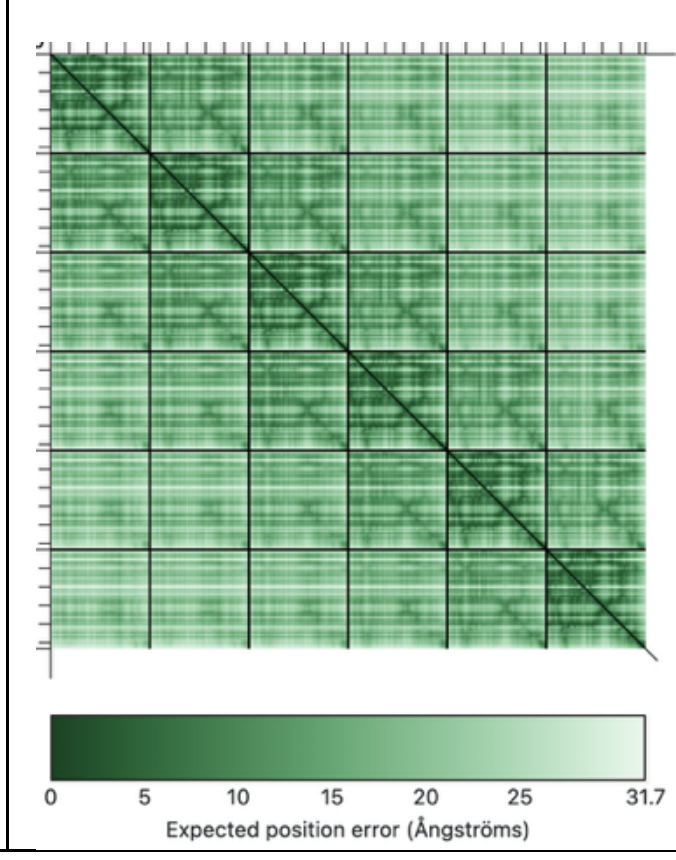

Bas69	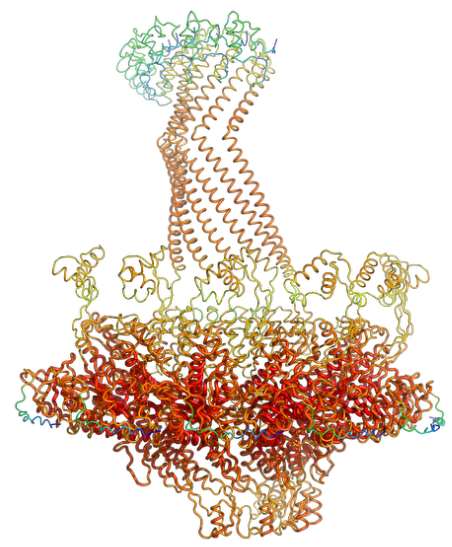	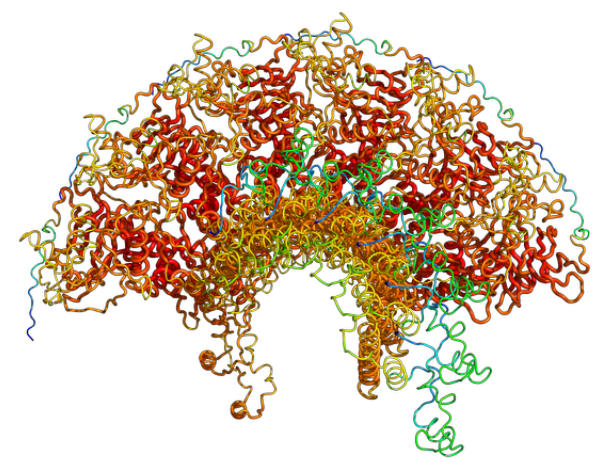	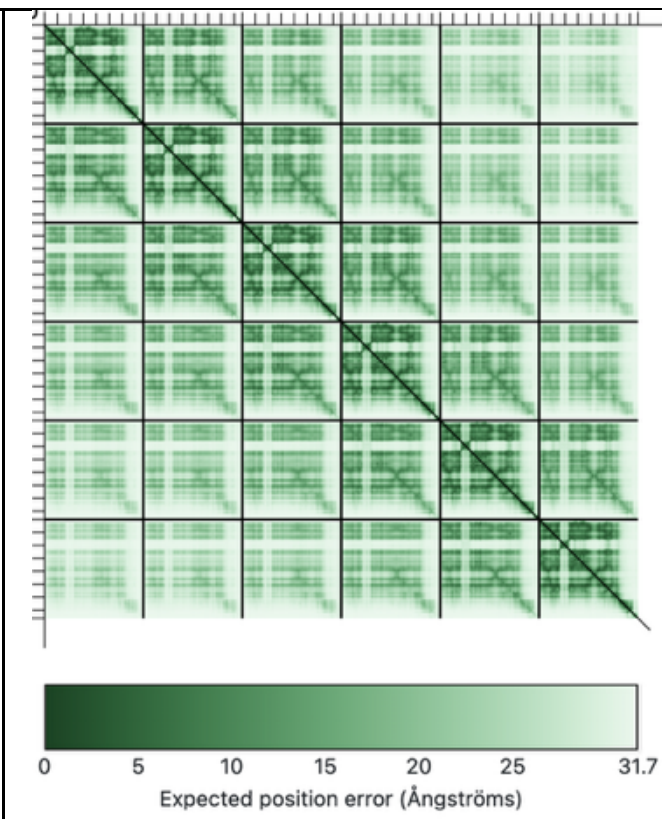

Supplementary table 2. Plaque formation of wt MG1655 or Δ*relA*Δ*spoT* by 16 different coliphages out of the BASEL collection

Representative plates from two independent biological replicates are shown.

Names of phage and surface charge of their predicted structures of portal ring	Plaques of infection of wild type MG1655	Plaques of infection of Δ*relA*Δ*spoT* of MG1655	*ΔrelAΔspoT is more sensitive than wt?*
T5 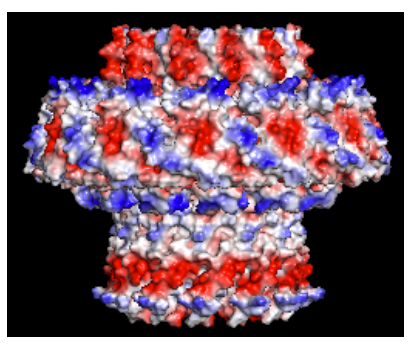	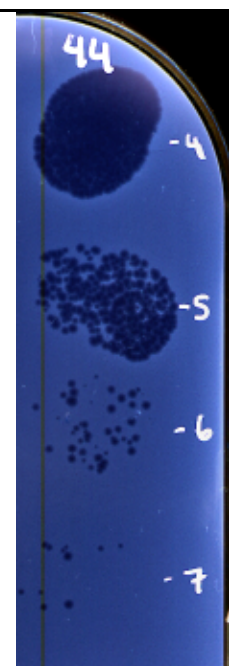	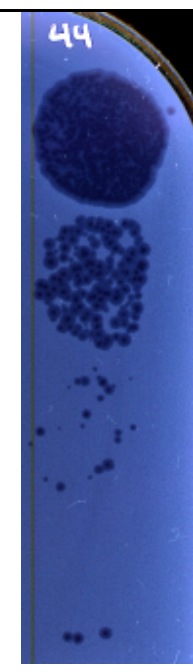	+/-
Lambdavir/HK 97 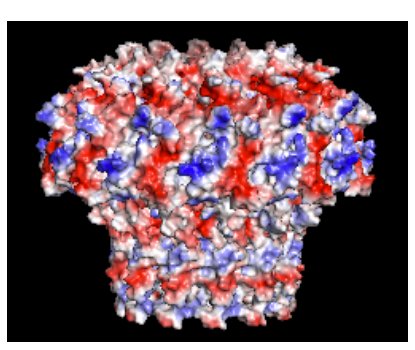	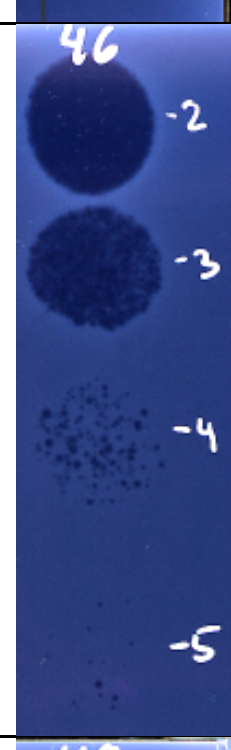	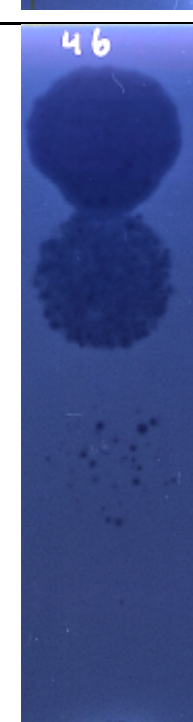	NO
T2 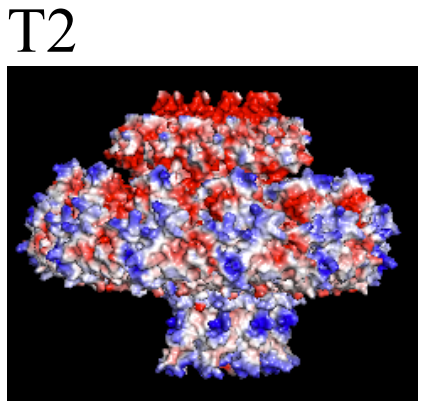	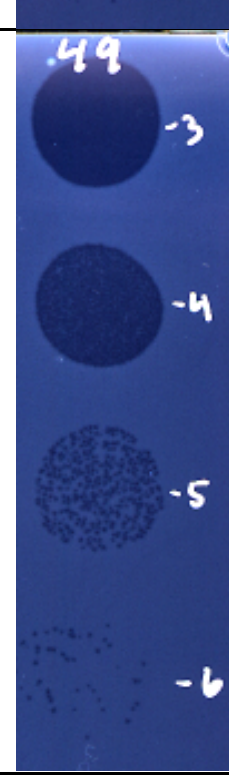	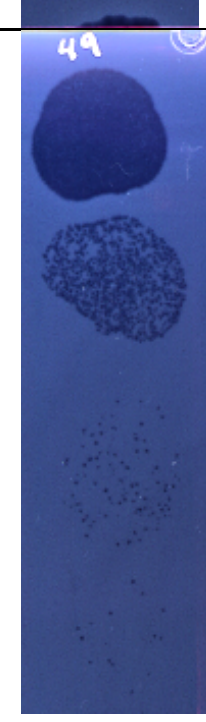	NO

Bas02 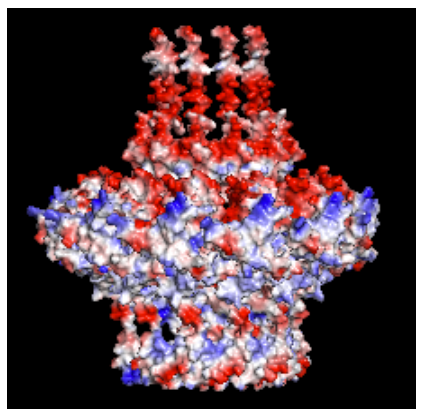	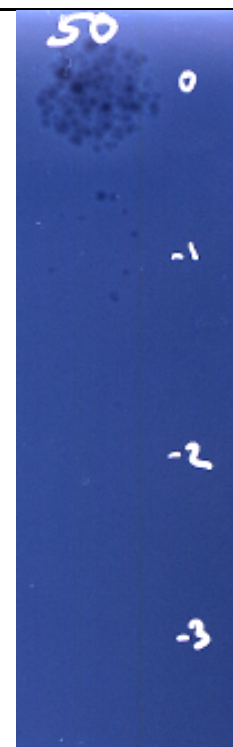	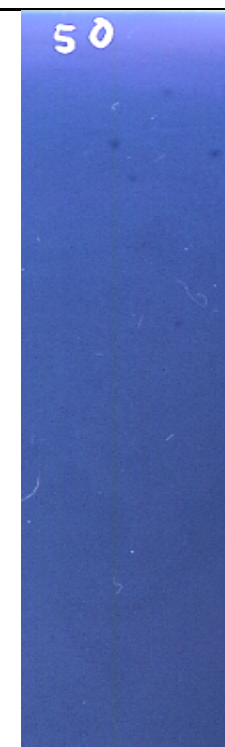	NO
Bas11 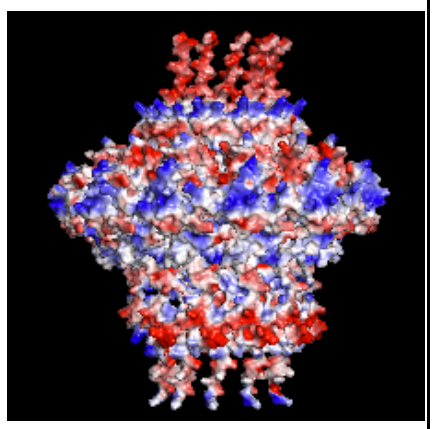	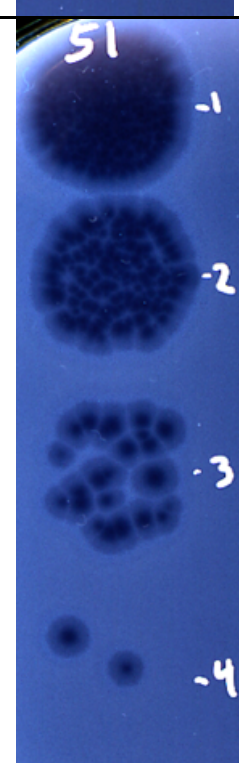	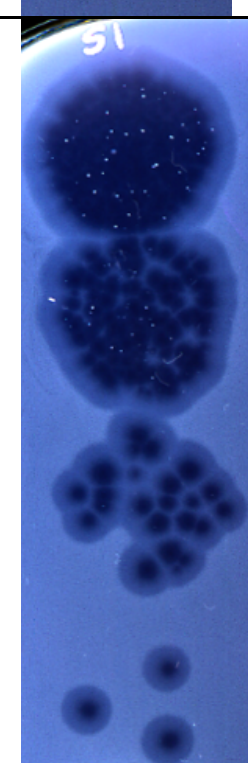	+/-
Bas12 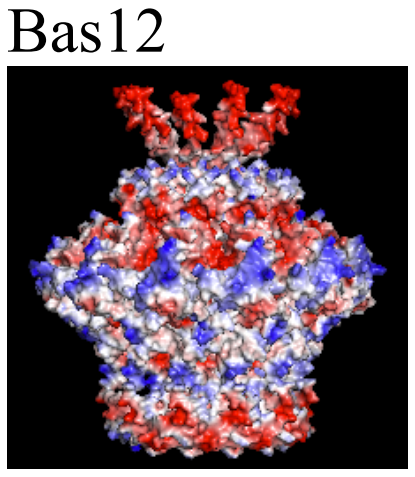	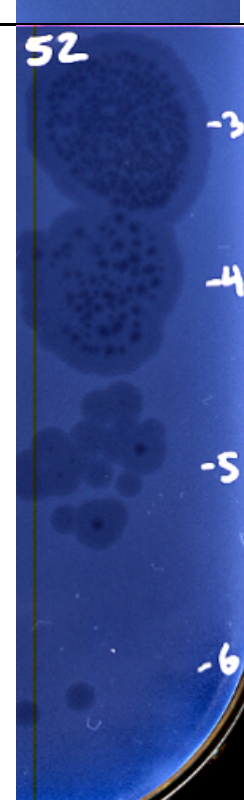	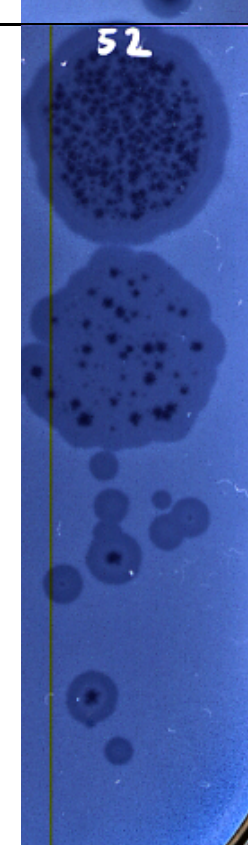	Yes, darker/clearer plaques
Bas17 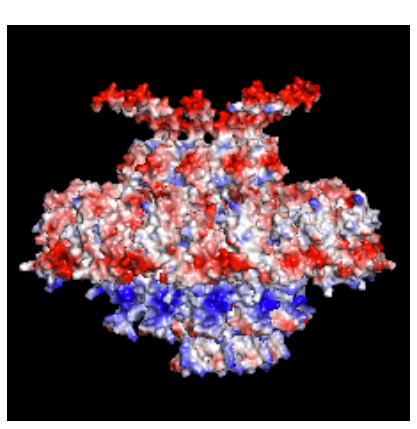	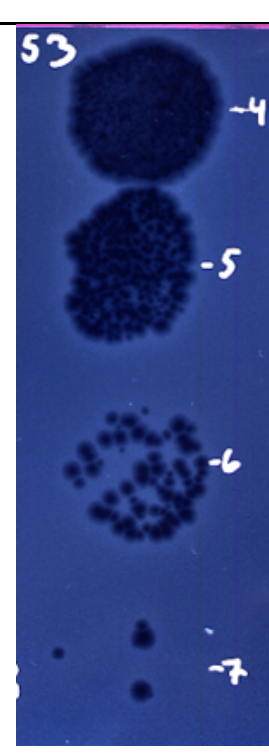	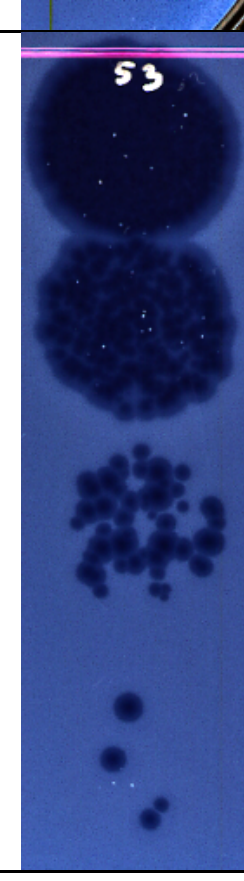	Yes, bigger and clearer plaques

Bas23 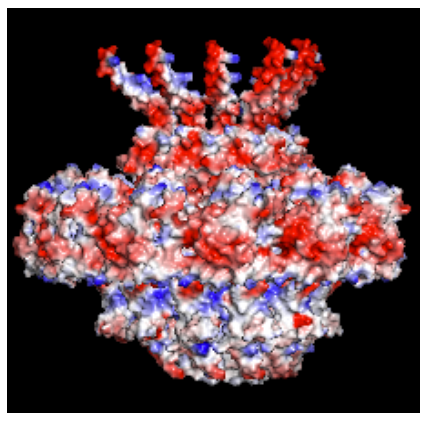	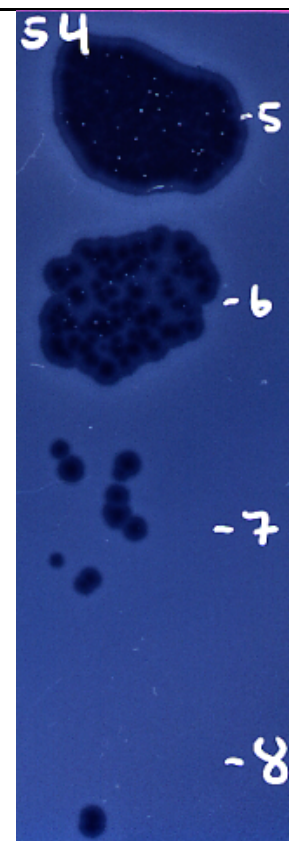	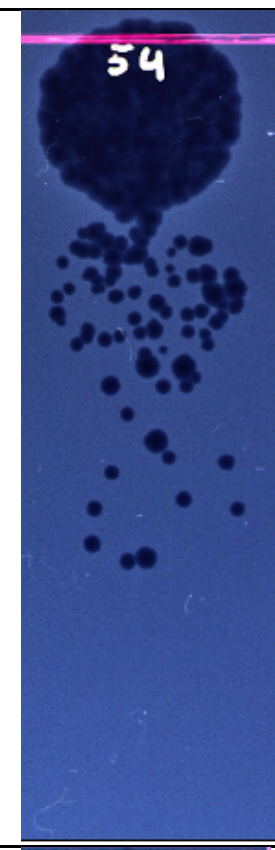	Yes?<br>darker plaques without halo
Bas26 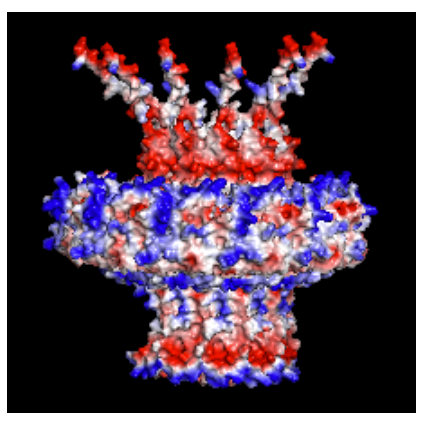	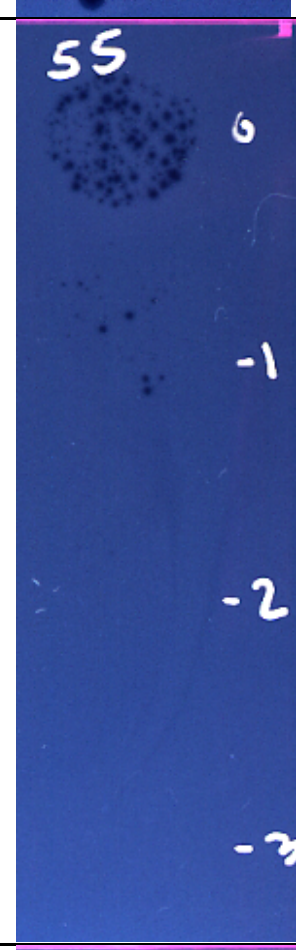	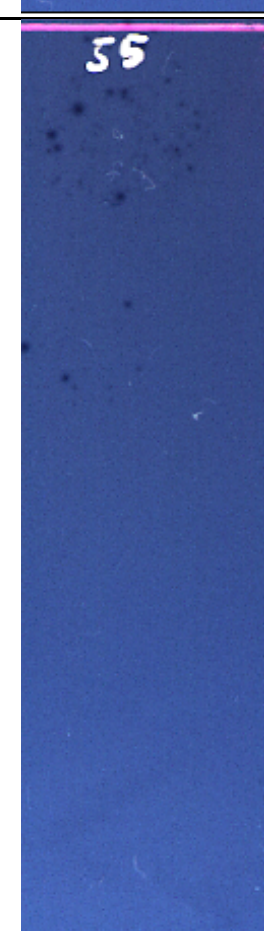	NO
Bas51 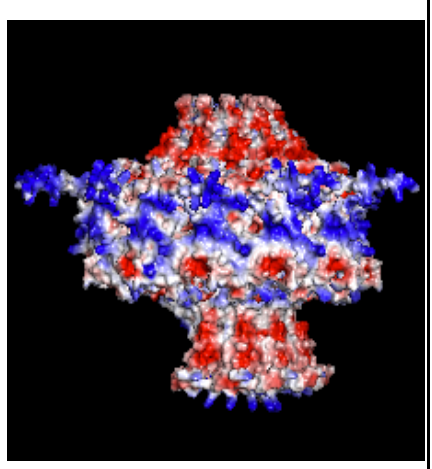	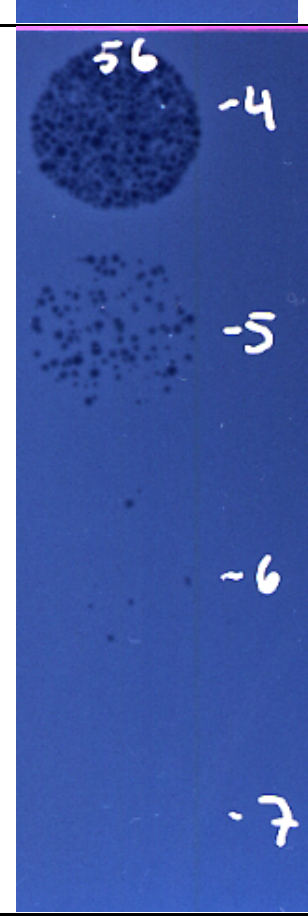	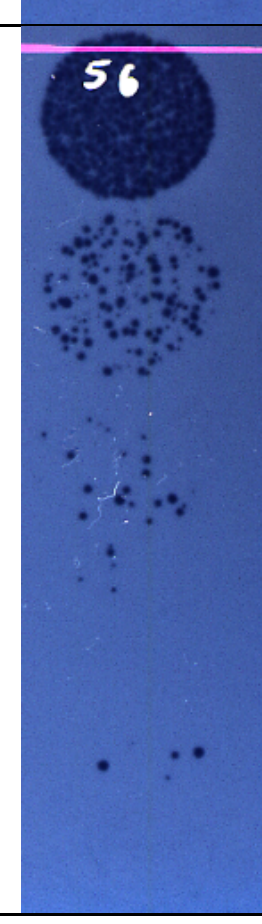	Yes, more PFU, bigger, darker plaques

Bas63 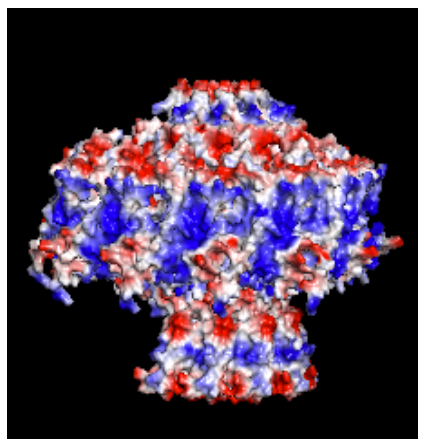	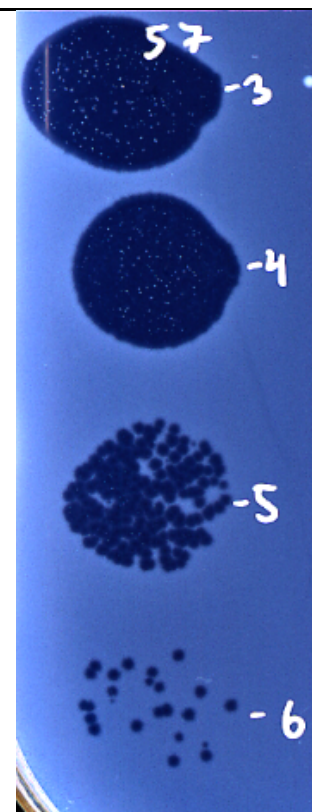	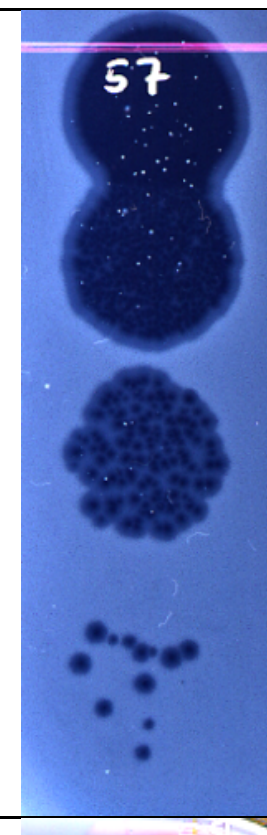	+/- plaques are bigger but unclear
Bas64 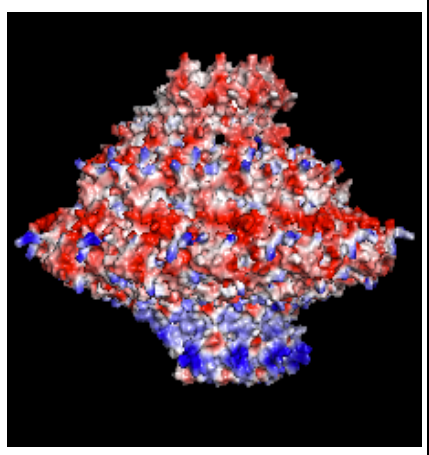	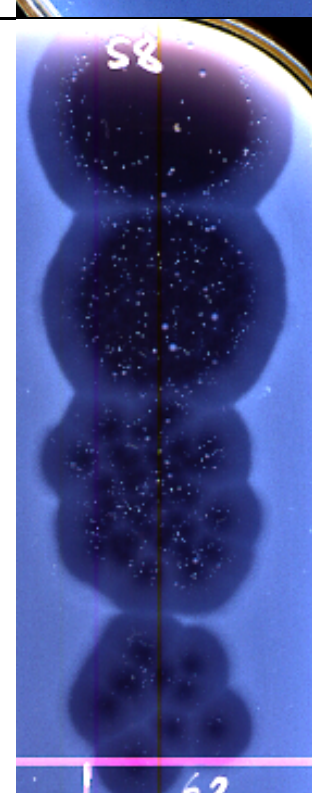 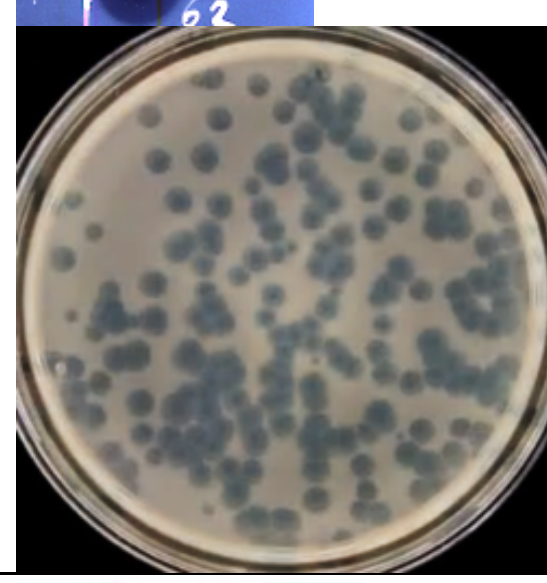	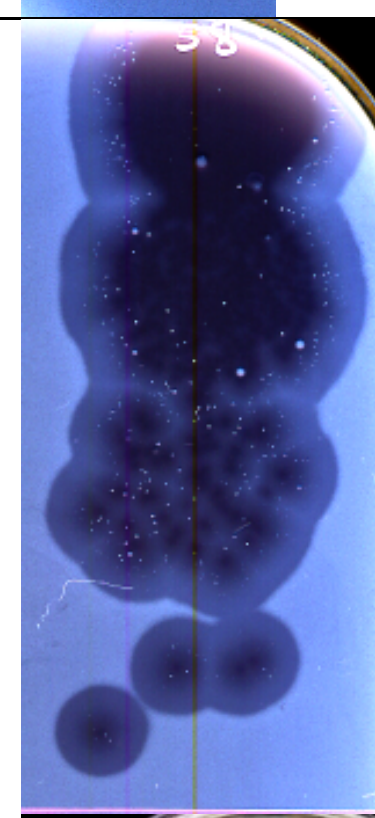 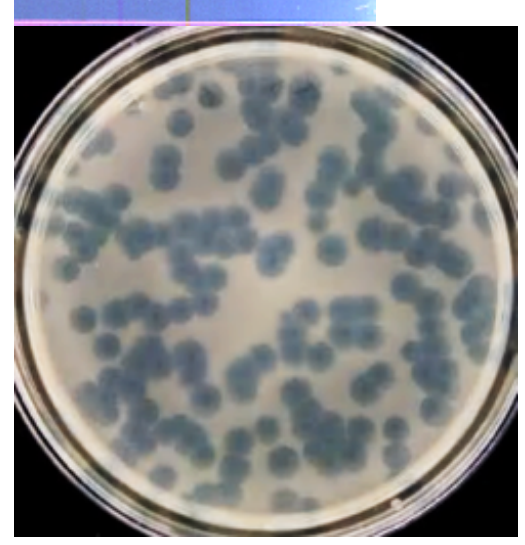	Yes, bigger plaques, more complete lysis
Bas65 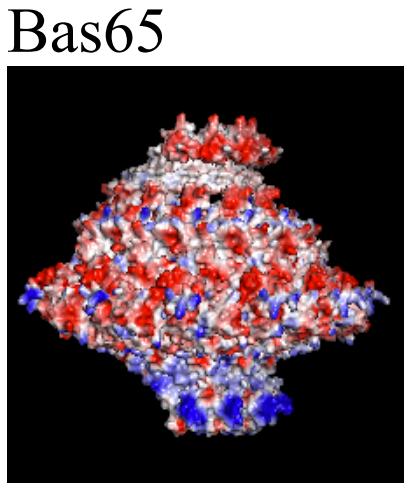	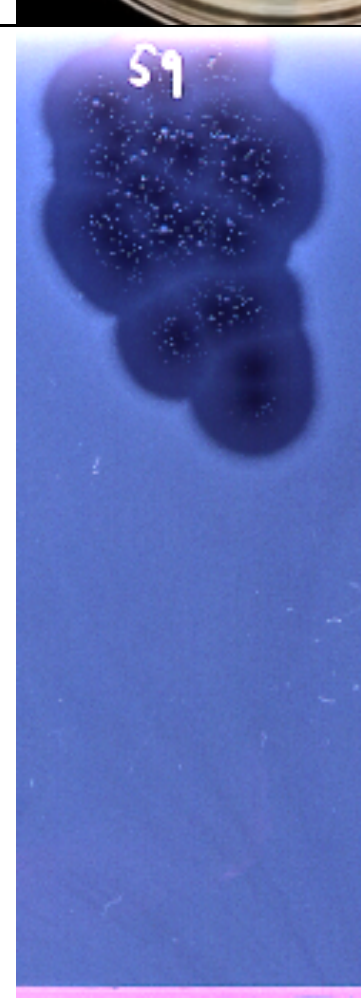	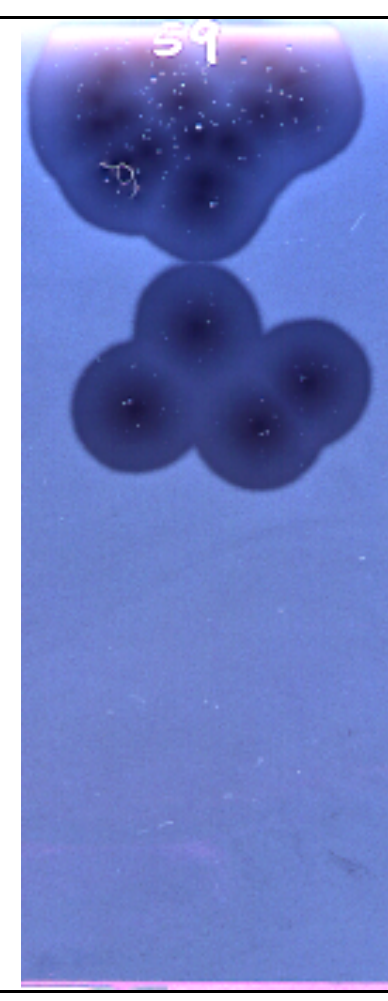	Yes, bigger plaques

Bas66/T3<br>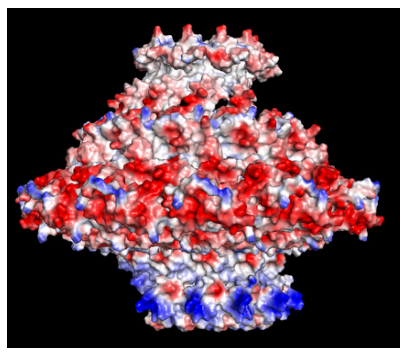	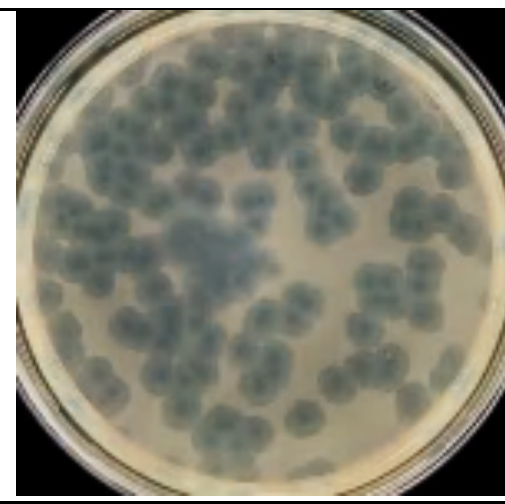	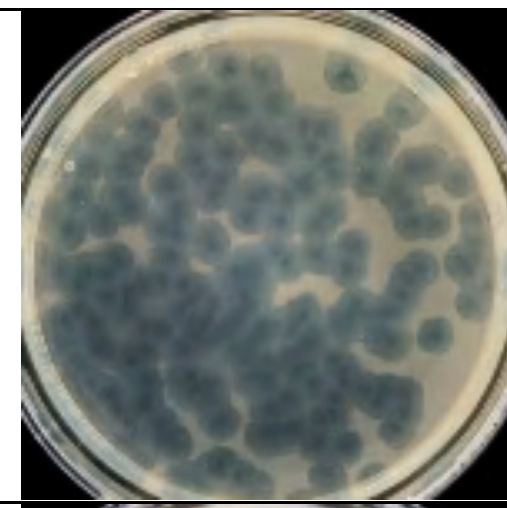	Yes, bigger and darker plaques
Bas68<br>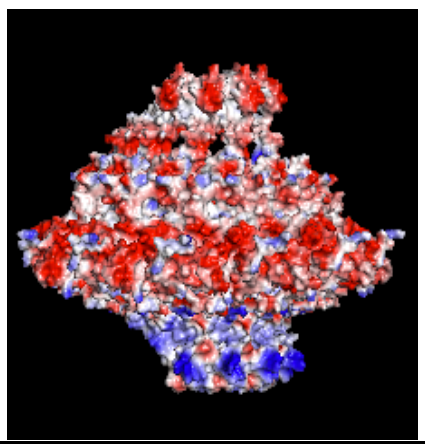	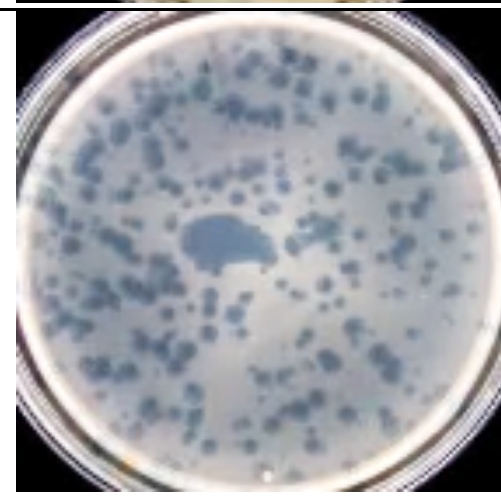	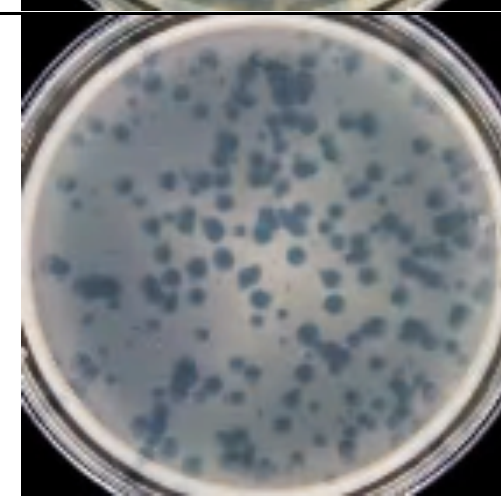	Yes, darker plaques
Bas69<br>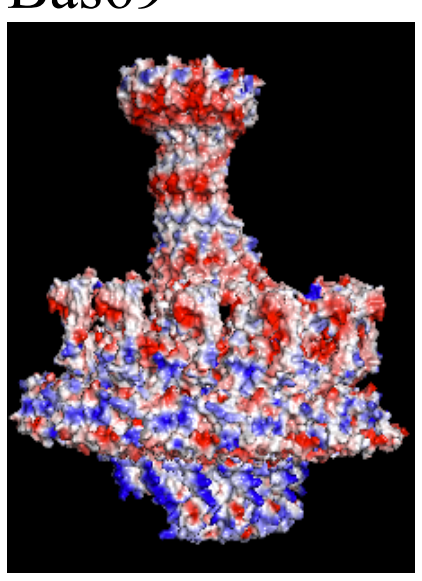	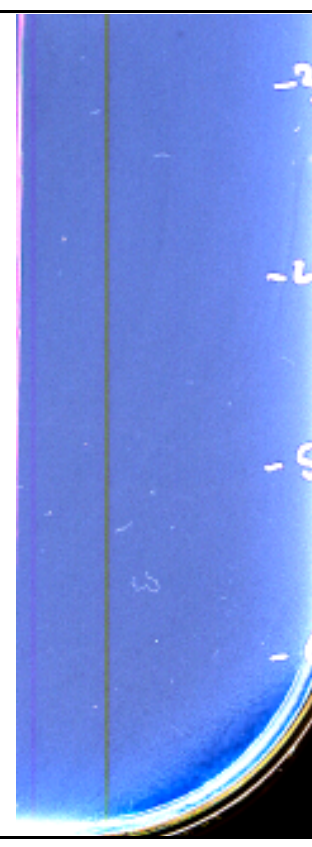	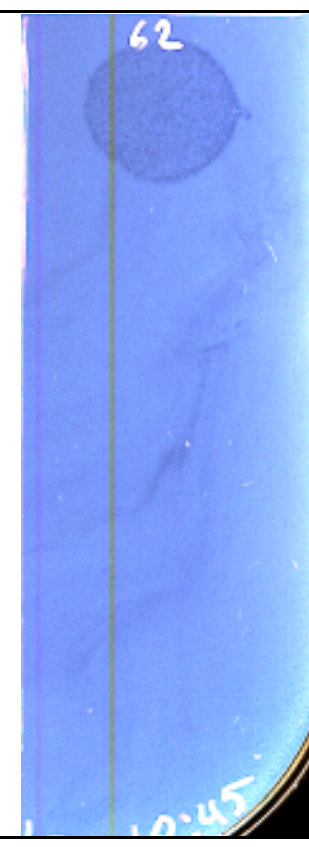	Yes, more PFU

Supplementary table 3 All strains used in this study

Number	Host	Plasmid or genotype	Reference
YZ8	BL21 DE3	pENH385-relseq.his	[1]
YZ15	DH5a	pCA24N	[2]
YZ38	MG1655	Δ*relA*	Laboratory strain
YZ44	BT340	pCP20	Laboratory strain
YZ142	MG1655	Wild-type strain	Laboratory strain
YZ213	DH5a	pBAD30	Laboratory strain
YZ307	DH5α	pET28-His6.SUMO.spoT	
YZ584	MG1655 Δ*relA*	*spoT203* (R140C)	[3]
YZ585	MG1655 Δ*relA*	*spoT202* (T78I) (4× basal levels of (p)ppGpp)	[3]
YZ586	MG1655 Δ*relA*	spoT (E319Q)	[4]
YZ666	BTH101	F-, *cya*-99, *araD*139, *galE*15, *galK*16, *rpsL*1 (Str^R), *hsdR*2, *mcrA*1, *mcrB*1	Laboratory strain
YZ705	DH5α	pKT25	Laboratory strain
YZ709	DH5α	pUT18C	Laboratory strain
YZ710	DH5a	pUT18C-*relA*	This study
YZ711	DH5a	pUT18C-*spoT*	This study
YZ712	DH5a	pKT25-*relA*	This study
YZ716	DH5a	pKT25-*spoT*	This study
YZ749	DH5a	pUT18C-*spoT*(HS)	This study
YZ750	DH5a	pUT18C-*spoT*(HST)	This study
YZ751	DH5a	pUT18C-*spoT*(TCA)	This study
YZ752	DH5a	pUT18C-*spoT*(CA)	This study
YZ753	DH5a	pKT25-*spoT*(HS)	This study
YZ754	DH5a	pKT25-*spoT*(HST)	This study
YZ755	DH5a	pKT25-*spoT*(TCA)	This study
YZ756	DH5a	pKT25-*spoT*(CA)	This study
YZ840	MG1655	Δ*relA*Δ*spoT*	[3]
YZ905	MG1655 Δ*relA*	spoT (H72AD73A)	[4]
YZ913	DH5α	pET28a-MBP	This study
YZ920	MG1655	*rpoB3449* (encoding RpoBΔAla532)	Gifted by Michael J Gray[5]

YZ921	MG1655 *ΔrelA782 spoT207::cat*	*rpoB3449*	Gifted by Michael J Gray[5]
YZ922	MG1655	*rpoB3443*	Gifted by Michael J Gray[6]
YZ1063	Rosetta	pET28-his6.MBP.SUMO.*spoT* (E319Q)	This study
YZ1070	Rosetta	pET28-his6.MBP.SUMO.*spoT* (H72D73AA)	This study
YZ1607	BL21 DE3	pET28b-his6.MBP.SUMO-*relA*	This study
YZ1381	DH5a	pUT18-*rpoZ*	This study
YZ1382	DH5a	pKNT25-*rpoZ*	This study
YZ1383	DH5a	pUT18C-T7*gp4.2*	This study
YZ1385	DH5a	pUT18C-T7*gp1.5*	This study
YZ1387	DH5a	pUT18C-T7*gp8*	This study
YZ1388	DH5a	pUT18C-T7*gp6.5*	This study
YZ1389	DH5a	pKT25-T7*gp4.2*	This study
YZ1391	DH5a	pKT25-T7*gp1.5*	This study
YZ1393	DH5a	pKT25-T7*gp8*	This study
YZ1394	DH5a	pKT25-T7*gp6.5*	This study
YZ1515	DH5a	pET28b-his.mbp.SUMO	This study
YZ1550	DH5a	pUT18C-*relA*(HS)	This study
YZ1551	DH5a	pUT18C-*relA*(HST)	This study
YZ1552	DH5a	pUT18C-*relA*(TCA)	This study
YZ1553	DH5a	pUT18C-*relA*(CA)	This study
YZ1615	BL21 DE3	pRARE, pET28b-HMS, pBAD30-T7*gp8*-Flag	This study
YZ1620	BL21 DE3	pRARE, pET28b-HMS-*relA*, pBAD30-T7*gp8*-Flag	This study
YZ1625	BL21 DE3	pRARE, pET28b-HMS-*spoT*, pBAD30-T7*gp8*-Flag	This study
YZ1632	BL21 DE3	pET28a-T7*gp8*-his7	This study
YZ1705	DH5a	pKT25-*gp8*(Δclip)	This study
YZ1706	DH5a	pKT25-*gp8*(Δ(clip-stem))	This study
YZ1707	DH5a	pKT25-*gp8*(ΔNt)	This study
YZ1708	DH5a	pKT25-*gp8*(ΔCt)	This study
KEIO collection	BW25113	*trxA::*Kan^R	Keio collection[7]
YZ1763	MG1655	*trxA::*Kan^R	This study
YZ1836	BL21 DE3	pRARE, pET28b-HMS, pBAD30-T7*gp8*(D220R/E221R)-Flag	This study
YZ1837	BL21 DE3	pRARE, pET28b-HMS, pBAD30-T7*gp8*(E10K/D11K)-Flag	This study

YZ1838	BL21 DE3	pRARE, pET28b-HMS-*relA*, pBAD30-T7*gp8*(D220R/E221R)-Flag	This study
YZ1839	BL21 DE3	pRARE, pET28b-HMS-*relA*, pBAD30-T7*gp8*(E10K/D11K)-Flag	This study
YZ1840	BL21 DE3	pRARE, pET28b-HMS-*spoT*, pBAD30-T7*gp8*(D220R/E221R)-Flag	This study
YZ1841	BL21 DE3	pRARE, pET28b-HMS-*spoT*, pBAD30-T7*gp8*(E10K/D11K)-Flag	This study
YZ1296	MG1655	pBAD30-*gp8*-Flag	This study
YZ1857	MG1655	pBAD30-*gp8* (D220R/E221R)-Flag	This study
YZ1878	DH5a	pBAD33-*gp59* (N4 portal protein)	This study
YZ 1879	iVEC	pBAD33-P1 portal protein	This study
YZ1993	DH5a	pKT25-*gp8*(D46K/D48K/D53K)	This study
YZ1994	DH5a	pKT25-*gp8*(D99K/D101K)	This study
YZ1995	DH5a	pKT25-*gp8*(D220R/E221R/D222R/E225R)	This study
YZ1996	DH5a	pKT25-*gp8*(D220R/E221R)	This study
YZ1997	DH5a	pKT25-*gp8*(D222R/E225R)	This study
YZ1998	DH5a	pKT25-*gp8*(D241K)	This study
YZ1999	DH5a	pKT25-*gp8*(D457K/D460K)	This study
YZ2000	DH5a	pKT25-*gp8*(D502R)	This study
YZ2001	DH5a	pKT25-*gp8*(E10K/D11K)	This study
YZ2002	DH5a	pKT25-*gp8*(E149K/E151K)	This study
YZ2003	DH5a	pKT25-*gp8*(E192R/D193K)	This study
YZ2004	DH5a	pKT25-*gp8*(E199R/E204R)	This study
YZ2005	DH5a	pKT25-*gp8*(E236R)	This study
YZ2006	DH5a	pKT25-*gp8*(E247R)	This study
YZ2007	DH5a	pKT25-*gp8*(E280R/E284R)	This study
YZ2008	DH5a	pKT25-*gp8*(E416K/E423K)	This study
YZ2009	DH5a	pKT25-*gp8*(E485K/E486K)	This study
YZ2010	DH5a	pKT25-*gp8*(E521R/D528R)	This study
YZ2011	DH5a	pKT25-*gp8*(K4D/R5D)	This study
YZ2012	DH5a	pKT25-*gp8*(K94D)	This study
YZ2013	DH5a	pKT25-*gp8*(K105E)	This study
YZ2014	DH5a	pKT25-*gp8*(K196E)	This study
YZ2015	DH5a	pKT25-*gp8*(K288D/K295D)	This study
YZ2016	DH5a	pKT25-*gp8*(K419D)	This study
YZ2017	DH5a	pKT25-*gp8*(R277D)	This study
YZ1794	MG1655 Δ*trxA*	pCA24N-*gp8*(D220R/E221R)	This study

YZ1796	MG1655 Δ*trxA*	pCA24N-*gp8*(E10KD11K)	This study
BP-E-A1	MG1655	pBAD33-*gp0.3*	This study
BP-E-A3	MG1655	pBAD33-*gp1.2*	This study
BP-E-A4	MG1655	pBAD33-*gp1.6*	This study
BP-E-A5	MG1655	pBAD33-*gp2.5*	This study
BP-E-A6	MG1655	pBAD33-*gp3.8*	This study
BP-E-A7	MG1655	pBAD33-*gp4.2*	This study
BP-E-B1	MG1655	pBAD33-*gp0.4*	This study
BP-E-B2	MG1655	pBAD33-*gp0.7*	This study
BP-E-B3	MG1655	pBAD33-*gp1.3*	This study
BP-E-B4	MG1655	pBAD33-*gp1.7*	This study
BP-E-B5	MG1655	pBAD33-*gp2.8*	This study
BP-E-B7	MG1655	pBAD33-*gp4.3*	This study
BP-E-C1	MG1655	pBAD33-*gp0.5*	This study
BP-E-C3	MG1655	pBAD33-*gp1.4*	This study
BP-E-C4	MG1655	pBAD33-*gp1.8*	This study
BP-E-C5	MG1655	pBAD33-*gp3*	This study
BP-E-C7	MG1655	pBAD33-*gp4.5*	This study
BP-E-C8	MG1655	pBAD33-*gp5.5*	This study
BP-E-D1	MG1655	pBAD33-*gp0.6A*	This study
BP-E-D2	MG1655	pBAD33-*gp1.1*	This study
BP-E-D3	MG1655	pBAD33-*gp1.5*	This study
BP-E-D4	MG1655	pBAD33-*gp2*	This study
BP-E-D5	MG1655	pBAD33-*gp3.5*	This study
BP-E-D6	MG1655	pBAD33-*gp4.1*	This study
BP-E-D7	MG1655	pBAD33-*gp4.7*	This study
BP-E-D8	MG1655	pBAD33-*gp5.7*	This study
BP-E-E1	MG1655	pBAD33-*gp5.9*	This study
BP-E-E2	MG1655	pBAD33-*gp6.7*	This study
BP-E-E3	MG1655	pBAD33-*gp8*	This study
BP-E-E4	MG1655	pBAD33-*gp11*	This study
BP-E-E5	MG1655	pBAD33-*gp15*	This study
BP-E-E6	MG1655	pBAD33-*gp18*	This study
BP-E-E7	MG1655	pBAD33-*gp19.2*	This study
BP-E-F1	MG1655	pBAD33-*gp6*	This study
BP-E-F2	MG1655	pBAD33-*gp7*	This study
BP-E-F3	MG1655	pBAD33-*gp9*	This study
BP-E-F4	MG1655	pBAD33-*gp12*	This study
BP-E-F5	MG1655	pBAD33-*gp16*	This study
BP-E-F6	MG1655	pBAD33-*gp18.5*	This study
BP-E-F7	MG1655	pBAD33-*gp19.3*	This study
BP-E-G1	MG1655	pBAD33-*gp6.3*	This study
BP-E-G2	MG1655	pBAD33-*gp7.3*	This study

BP-E-G3	MG1655	pBAD33-*gp10A*	This study
BP-E-G4	MG1655	pBAD33-*gp13*	This study
BP-E-G5	MG1655	pBAD33-*gp17*	This study
BP-E-G6	MG1655	pBAD33-*gp18.7*	This study
BP-E-G7	MG1655	pBAD33-*gp19.5*	This study
BP-E-H1	MG1655	pBAD33-*gp6.5*	This study
BP-E-H2	MG1655	pBAD33-*gp7.7*	This study
BP-E-H3	MG1655	pBAD33-*gp10B*	This study
BP-E-H4	MG1655	pBAD33-*gp14*	This study
BP-E-H5	MG1655	pBAD33-*gp17.5*	This study
BP-E-H6	MG1655	pBAD33-*gp19*	This study

Supplementary table 4 All primers used in this study

Primers	Description	Sequence
0.3-F	Amplify gp0.3 fromT7 and NcoI restriction sequence	CATGccatggCTATGTCTAACATGACTTAC
0.3-R	Amplify gp0.3 fromT7 and EcoRI restriction sequence	gGAATTCttactcttcatcctcctcgta
0.4-F	Amplify gp0.4 fromT7 and with 17 bp flanking sequence for iVEC cloning in pBAD33	GAAGGAGATATACCATGtctactaccaacgtgc
0.4-R	Amplify gp0.4 fromT7 and 17 bp flanking sequence for iVEC cloning in pBAD33	cgactctagaggatTTActcagcagattctaaagctattg
0.5-F	Amplify gp0.5fromT7 and NcoI restriction sequence	CATGccatggGTTATATGCTTACTATCGGTCT ACTC
0.5-R	Amplify gp0.5 fromT7 and EcoRI restriction sequence	gGAATTCtcatttgcgtagtgcc
0.6A-F	Amplify gp0.6A andgp0.6B fromT7 and NcoI restriction sequence	CATGccatggGTATGAAGCACTACGTTATGC
0.6A-R	Amplify gp0.6A fromT7 and EcoRI restriction sequence	gGAATTCtcagtgcgttctgtcatag
0.7-F	Amplify gp0.7 fromT7 and with 17 bp flanking sequence for iVEC cloning in pBAD33	GAAGGAGATATACCATGaacattaccgacatcat
0.7-R	Amplify gp0.7 fromT7 and 17 bp flanking sequence for iVEC cloning in pBAD33	cgactctagaggatTTAgcccattaacattgcg
1.1-F	Amplify gp1.1 fromT7 and NcoI restriction sequence	CATGccatggGTCGTAACTTCGAAAAGATGA C
1.1-R	Amplify gp1.1 fromT7 and EcoRI restriction sequence	gGAATTCttactgaccctcccagctac
1.2-F	Amplify gp1.2 fromT7 and NcoI restriction sequence	CATGccatggGACGTTTATATAGTGGTA
1.2-R	Amplify gp1.2 fromT7 and EcoRI restriction sequence	gGAATTCttacttccagtccttcaact
1.3-F	Amplify gp1.3 fromT7 and NcoI restriction sequence	CATGccatggGTATGAACATTAAGACTAACC CG
1.3-R	Amplify gp1.3fromT7 and EcoRI restriction sequence	gGAATTCttacattttctcttgagggttg

1.4-F	Amplify gp1.4 fromT7 and NcoI restriction sequence	CATGccatggGTTTTAAGAAGGTTGGTAAATTCC
1.4-R	Amplify gp1.4 fromT7 and EcoRI restriction sequence	gGAATTCttaccagttaactatactccacacg
1.5-F	Amplify gp1.5 fromT7 and NcoI restriction sequence	CATGccatggGTATGTACTTAATGCCATTACTCATC
1.5-R	Amplify gp1.5 fromT7 and EcoRI restriction sequence	gGAATTCttaagcgtgaccatctgg
1.6-F	Amplify gp1.6 fromT7 and NcoI restriction sequence	CATGccatggGTTTTCGACTTCATTACAACAAAG
1.6-R	Amplify gp1.6 fromT7 and EcoRI restriction sequence	gGAATTCtcagaacacctccttgattc
1.7-F	Amplify gp1.7 fromT7 and NcoI restriction sequence	CATGccatggGACTGTTAGATGGTG
1.7-R	Amplify gp1.7fromT7 and EcoRI restriction sequence	gGAATTCttatgcataacatttatccttatg
1.8-F	Amplify gp1.8 fromT7 and NcoI restriction sequence	CATGccatggGTCATAACTTCAAGTCAACCCC
1.8-R	Amplify gp1.8 fromT7 and EcoRI restriction sequence	gGAATTCtcattgacctctcgatttc
2-F	Amplify gp2 fromT7 and NcoI restriction sequence	CATGccatggGTTCAAACGTAAATACAGGTTCAC
2-R	Amplify gp2fromT7 and EcoRI restriction sequence	gGAATTCttacttcggtgctacacaag
2.5-F	Amplify gp2.5 fromT7 and NcoI restriction sequence	CATGccatggCTAAGAAGATTTTCACC
2.5-R	Amplify gp2.5 fromT7 and EcoRI restriction sequence	gGAATTCttagaagtctccgtcttcgtc
2.8-F	Amplify gp2.8 fromT7 and NcoI restriction sequence	CATGccatggAACTGCGGGAGAA
2.8-R	Amplify gp2.8 fromT7 and EcoRI restriction sequence	gGAATTCttagcgccgtaacctg
3-F	Amplify gp3 fromT7 and NcoI restriction sequence	CATGccatggCAGGTTACGGCG
3-R	Amplify gp3 fromT7 and EcoRI restriction sequence	gGAATTCttatttctttcctcctttcctt
3.5-F	Amplify gp3.5 fromT7 and NcoI restriction sequence	CATGccatggCTCGTGTACAGTTTAAAC
3.5-R	Amplify gp3.5 fromT7 and EcoRI restriction sequence	gGAATTCttatccacggtcagaagtg

3.8-F	Amplify gp3.8 fromT7 and NcoI restriction sequence	CATGccatggGTCGCAAGTCTTATAAACAATTCTAT
3.8-R	Amplify gp3.8 fromT7 and EcoRI restriction sequence	gGAATTCctatcggaatcgtgcg
4.1-F	Amplify gp4.1 fromT7 and NcoI restriction sequence	CATGccatggGAACTCGCTGTTCT
4.1-R	Amplify gp4.1 fromT7 and EcoRI restriction sequence	gGAATTCtcattggtttacctcctgag
4.2-F	Amplify gp4.2 fromT7 and NcoI restriction sequence	CATGccatggGTAGGAAGGTCGCCCCGTTT
4.2-R	Amplify gp4.2 fromT7 and EcoRI restriction sequence	gGAATTCttagaatgggactctccagc
4.3-F	Amplify gp4.3 fromT7 and NcoI restriction sequence	CATGccatggGTTTCAAACTGATTAAGAAGTTAGGC
4.3-R	Amplify gp4.3 fromT7 and EcoRI restriction sequence	gGAATTCttactcaaagaatttggaaagc
4.5-F	Amplify gp4.5 fromT7 and NcoI restriction sequence	CATGccatggGTTCTAACGTAGCTGAAACTATCC
4.5-R	Amplify gp4.5 fromT7 and EcoRI restriction sequence	gGAATTCttagttatcaatcttgtcgagg
4.7-F	Amplify gp4.7 fromT7 and NcoI restriction sequence	CATGccatggGTCGTGACCCTAAAGTTATCC
4.7-R	Amplify gp4.7 fromT7 and EcoRI restriction sequence	gGAATTCttatcgtgacttaacaatctcttc
5.5-F	Amplify gp5.5 fromT7 and NcoI restriction sequence	CATGccatggCTATGACAAAGAAATTTAAAG
5.5-R	Amplify gp5.5 fromT7 and EcoRI restriction sequence	gGAATTCtcagaacacctcccgtact
5.7-F	Amplify gp5.7 fromT7 and NcoI restriction sequence	CATGCCATGGGTTCTGACTACCTGAAAGTGCTG
5.7-R	Amplify gp5.7 fromT7 and EcoRI restriction sequence	gGAATTCttagacacatcctcccattc
5.9-F	Amplify gp5.9fromT7 and NcoI restriction sequence	CATGccatggGTTCTCGTGACCTTGTGAC
5.9-R	Amplify gp5.9 fromT7 and EcoRI restriction sequence	gGAATTCtcaagaagtgccattaagtttc
6-F	Amplify gp6.0 fromT7 and NcoI restriction sequence	CATGccatggCACTTCTTGACCT
6-R	Amplify gp6.0 fromT7 and EcoRI restriction sequence	gGAATTCctacggtctccacaggtaaat

6.3-F	Amplify gp6.3 fromT7 and NcoI restriction sequence	CATGccatggAGACCGTAGCGT
6.3-R	Amplify gp6.3 fromT7 and EcoRI restriction sequence	gGAATTCtcacttagtgtcgtgtaacg
6.5-F	Amplify gp6.3 fromT7 and NcoI restriction sequence	CATGccatggGTTTAACACCTATTAACCAATTACTTAAG
6.5-R	Amplify gp6.3 fromT7 and EcoRI restriction sequence	gGAATTCtcaatcctccccatcatc
6.7-F	Amplify gp6.7 fromT7 and NcoI restriction sequence	CATGccatggGTTGTTTCTCACCGAAAATTAAAAC
6.7-R	Amplify gp6.7 fromT7 and EcoRI restriction sequence	gGAATTCtcacttcttacctccaaatg
7-F	Amplify gp7 fromT7 and NcoI restriction sequence	CATGccatggGTTCTGAGTTCACATGTGTGG
7-R	Amplify gp7 fromT7 and EcoRI restriction sequence	gGAATTCttatacctccttaaagtatacacgc
7.3-F	Amplify gp7.3 fromT7 and NcoI restriction sequence	CATGccatggGTAAGAAAGTTAAGAAGG
7.3-R	Amplify gp7.3 fromT7 and EcoRI restriction sequence	gGAATTCttaaatgttgataccgccac
7.7-F	Amplify gp7.7 fromT7 and NcoI restriction sequence	CATGccatggAAGACTGCATTGAATG
7.7-R	Amplify gp7.7 fromT7 and EcoRI restriction sequence	gGAATTCttaaatgtgtctccatgtcttac
8-F	Amplify gp8 fromT7 and NcoI restriction sequence	CATGccatggCTGAGAAACGAAC
8-R	Amplify gp8 fromT7 and EcoRI restriction sequence	gGAATTCttaaattcccggctgtaaac
9-F	Amplify gp9 fromT7 and NcoI restriction sequence	CATGccatggCTGAATCTAATGCAGAC
9-R	Amplify gp9 fromT7 and EcoRI restriction sequence	gGAATTCtcagaagttcgaatcgattac
10A-F	Amplify gp10A and gp10B fromT7 and NcoI restriction sequence	CATGccatggCTAGCATGACTGGT
10A-R	Amplify gp10A fromT7 and EcoRI restriction sequence	gGAATTCttactccactttgaaaaccac
10B-R	Amplify gp10B fromT7 and EcoRI restriction sequence	gGAATTCttattgctcagcggtg

11-F	Amplify gp11 fromT7 and NcoI restriction sequence	CATGccatggGTCGCTCATACGATATGAACG
11-R	Amplify gp11 fromT7 and EcoRI restriction sequence	gGAATTCttagcgagtcagtagaccag
12-F	Amplify gp12 fromT7 and NcoI restriction sequence	CATGccatggCACTCATTAGCC
12-R	Amplify gp12 fromT7 and EcoRI restriction sequence	gGAATTCttaaataccggaacttctcc
13-F	Amplify gp13 fromT7 and NcoI restriction sequence	CATGccatggGTATGACTATAAGACCTACTAAAAGTACAG
13-R	Amplify gp13 fromT7 and EcoRI restriction sequence	gGAATTCttatcctcctttcgtgattg
14-F	Amplify gp14 fromT7 and NcoI restriction sequence	CATGccatggGTTGTTGGGCAGCCGCAATAC
14-R	Amplify gp14 fromT7 and EcoRI restriction sequence	gGAATTCttacctccccgtcttg
15-F	Amplify gp15 fromT7 and with 21bp flanking sequence for iVEC cloning in pET28b	TAAGAAGGAGATATACCATGGgtagtaaaattgaatctgcccttc
15-R	Amplify gp15 fromT7 and 21 bp flanking sequence or iVEC cloning in pET28b	ttgtcgacggagctcgaattcTTACTCCTTACGTCCGTAGATG
16-F	Amplify gp16 fromT7 and NcoI restriction sequence	CATGccatggATAAGTACGATAAGAACG
16-R	Amplify gp16 fromT7 and EcoRI restriction sequence	gGAATTCttatttcctacgctccctc
17-F	Amplify gp17 fromT7 and NcoI restriction sequence	CATGccatggCTAACGTAATTAAAACC
17-R	Amplify gp17 fromT7 and EcoRI restriction sequence	gGAATTCttactcgttctccaccat
17.5-F	Amplify gp17.5 fromT7 and NcoI restriction sequence	CATGccatggGTCTATCATTAGACTTTAACAACGAAT
17.5-R	Amplify gp17.5 fromT7 and EcoRI restriction sequence	gGAATTCtcactccttattggctttct
18-F	Amplify gp18 fromT7 and NcoI restriction sequence	CATGccatggAAAAGGATAAGAGCC
18-R	Amplify gp18 fromT7 and EcoRI restriction sequence	gGAATTCtcactgtaatgtgtaaatatcatc
18.5-F	Amplify gp18.5 fromT7 and NcoI restriction sequence	CATGccatggGTCTAGAATTTTTACGTAAGCTAATCC

18.5-R	Amplify gp18.5 fromT7 and EcoRI restriction sequence	gGAATTCctacttacgttgcagttcac
18.7-F	Amplify gp18.7 fromT7 and NcoI restriction sequence	CATGccatggGTAGTACGTTAAGAGAGTTGAGGCT
18.7-R	Amplify gp18.7 fromT7 and EcoRI restriction sequence	gGAATTCtcactgcgagaatacgtttag
19-F	Amplify gp19 fromT7 and NcoI restriction sequence	CATGccatggGTTCTACTCAATCCAATCGTAATG
19-R	Amplify gp19 fromT7 and EcoRI restriction sequence	gGAATTCtcaccactcaatgaaagac
19.2-F	Amplify gp19.2 fromT7 and NcoI restriction sequence	CATGccatggGTACACAACCATTATCTG
19.2-R	Amplify gp19.2 fromT7 and EcoRI restriction sequence	gGAATTCtcagcgggtacttctcg
19.3-F	Amplify gp19.3 fromT7 and NcoI restriction sequence	CATGccatggCTACTCCGATAAGACC
19.3-R	Amplify gp19.3 fromT7 and EcoRI restriction sequence	gGAATTCtcataccacgggcac
19.5-F	Amplify gp19.5 fromT7 and NcoI restriction sequence	CATGccatggGTTTCCGCTTATTGTTGAAC
19.5-R	Amplify gp19.5 fromT7 and EcoRI restriction sequence	gGAATTCctaatcgctacaagtgagtatagag
pYZ473	XbaI-spoTNt-F	gCTCTAGAGCcttatctgtttg
pYZ474	XmaI-spoTSyn-R	tcccCCCGGGgTTACGGGAAGAGATCGGA
pYZ475	XmaI-spoTtgs-R	tcccCCCGGGgTTACGGAGCGGTAATGATTTC
pYZ476	XmaI-spoTCt-R	tcccCCCGGGGTTAATTTCG
pYZ477	XbaI-spoTtgs-F	gcTCTAGAGCctGATCTCTTCCCGGATGAG
pYZ478	XbaI-spoTcc-F	gcTCTAGAGCctGGCGCTCGCCCGAAT
pYZ479	XbaI-Spot-F	gcTCTAGAGCCTTATCTGTTTGAAAGCCTG
pYZ480	XmaI-Spot-R	tcccCCCGGGGTTAATTTCGGTTTCGGGTG
pYZ481	XbaI-relA-F	gcTCTAGAGCCTGTTGCGGTAAGAAGTG

pYZ482	XmaI-relA-R	tcccCCCGGGATTTAACTCCCGTGCAACC
pYZ684	EcoRI-SUMO-F	gGAATTCggttcggactcagaagtcaa
pYZ685	EcoRI-SUMO-R	gGAATTCtaataccgtcgtacaagaat
pYZ692	NcoI-His-sumo-F	ggccatggttcggactcagaagtcaatc
pYZ693	sumo-spoT-R	ttggtggttatctgtttgaaagcctgaatcaac
pYZ694	sumo-spoT-F	aacagataaccaccaatctgttctctgtgag
pYZ695	HindIII-spoT-R	ccgcaagcTTAATTTCGGTTTCGGGTGACTTTAATC
pYZ702	spoT-iVEC-cloning-F1	CAGGCGTATCTCGTTGCAC
pYZ703	spoT-iVEC-cloning-R1	CACTGCATAAGCGAAGTCG
pYZ862	QC-rmvNcoI-pkt25-R	CGTGACCgATGGCGATGCCTGCT
pYZ863	QC-rmvNcoI-pkt25-F	TCGCCATcGGTCACGACGAGATCCTC
pYZ864	QC-insNcoI-B2H-R	GAATTCccatgGGTACCCGGGGATCC
pYZ865	QC-insNcoI-pKT-F	GTACCcatggGAATTCACTGGCCGTCG
pYZ866	QC-insNcoI-pUT-F	GTACCcatggGAATTCATCGATATAACTAAGTAATATGGTG
pYZ867	HindIII-rpoZ-F	CCCaagcttaGCACGCGTAACTGTTCAG
pYZ868	XbaI-rpoZ-R	GCtctagactACGACGACCTTCAGCAATA
pYZ869	pK(N)T25-seq-R	ccttcgccacggccttgatg
pYZ870	pK(N)T25-seq-F	TTCCGGCTCGTATGTTG
pYZ871	QC-respKNT25-R	GAATTCGAGCTCGGTACCCGGGGATCCTCTAGAGTCGACCTGCAGGCATGCAAGCTTGGCGTAATCATGGTCATAGC

pYZ872	QC-respKNT25-F	GTACCGAGCTCGAATTCAATGACCATGCAGCAATCG
pYZ905	QCpET-HMS-BamHI-F	tggtGGATCCTAAGCTTGCGGCCGC
pYZ906	QCpET-HMS-BamHI-R	agcttAGGATCCACCAATCTGTTCTCTGTG
pYZ917	XbaI-relApUT18C-F	gcTCTAGAGCctGTTGCGGTA
pYZ918	XmaI-relAHST-R	TCCCcccgggaATCTTTACTGCGGTTTTGCG
pYZ919	XmaI-relAHS-R	TCCCcccgggaGTCAAAGACCTGACTACGTACTTC
pYZ920	XmaI-relApUT18C-R	TCCCcccgggaACTCCCGTGCAACCG
pYZ921	XbaI-relA(TCA)-F	gcTCTAGAGCctGTCTTTGACGACCGGGT
pYZ922	XbaI-relA(CA)-F	gcTCTAGAGCctGACAAAAACATTCTGGCTGG
pYZ927	BamHI-relA-F	CGggatccGTTGCGGTAAGAAGTGCA
pYZ928	HindIII-relA-R	CCCaagcttaACTCCCGTGCAACC
pYZ1063	*gp8*(clip deltion)-R	TCCAGTTGGCCGATAACCTTAGAGCTAATCA
pYZ1064	*gp8*(clip deltion)-F	ttatcggcCAACTGGAGAAGCAAGCAG
pYZ1065	*gp8*(clip and stem deltion)-R	AACATAAAgtacgaacgaccgtaggatt
pYZ1066	*gp8*(clip and stem deltion)-F	gttcgtacTTTATGTTGAACTCTGCGGTT
pYZ1067	*gp8*(N-terminus deletion)-R	TTAGGGAACATGGGTACCCGGGG
pYZ1068	*gp8*(N-terminus deletion)-F	TACCcatgTTCCCTAAGGACTCCGATAAC
pYZ1069	*gp8*(C-terminus deletion)-R	aattcTTAggtgagtagaataccagaagtgtc
pYZ1070	*gp8*(C-terminus deletion)-F	tactcaccTAAgaattcACTggccgtc
pYZ1187	Confirm deletion of trxA-F	CGAAAGCGTATCCGGTG

pYZ11 88	Confirm deletion of trxA-R	GCGTCCAGTTTTTAGCGAC
pYZ11 89	Amplify trxA from MG1655 with 40 bp gp8 flanking sequence-F	taacgcgaatactacagcgtaagacatggagacacatttaatgagcg ataaaattattcacc
pYZ11 90	Amplify trxA from MG1655 with 40 bp gp8 flanking sequence-R	cgctcatttcaaagatgaggtctccctatagtgagtcgtattacgccag gttagcg
pYZ11 91	Amplify gp8+350 bp flanking region and XhoI restriction sequence-F	CCGctcgagGGTTATGGTCGTAAGTGG
pYZ11 92	Amplify gp8+350 bp flanking region and HindIII restriction sequence-R	CCCaagcttCTCAGAGCCATCACCG
pYZ11 93	Confirm gp8 in cPCR-R	TCTTGTCCTCGACCAATTG
pYZ11 94	Confirm gp8 in cPCR-F	TATCTGGATGAGGACTCAGG
pYZ11 95	Confirm trxA gene in T7-F	AGCGTAAGACATGGAGACAC
pYZ11 96	Confirm trxA gene in T7-R	TCATTTCAAAGATGAGGTCTC
pYZ34	Confirm gp8 sequence on pCA24N-F	GGCCCTTTCGTCTTCACCTC
pYZ35	Confirm gp8 sequence on pCA24N-R	GGCAACCGAGCGTTCTGAAC
pYZ10 85	QC of gp8-D46KD48KD53K-F	AAGTCCAAGAACGCCTCTACAAAGTATCA AACTCCGTGGCAAG
pYZ10 86	QC of gp8-D46KD48KD53K-R	CTTTGTAGAGGCGTTCTTGGACTTCTTAGG GAACAATGATGGGA
pYZ10 87	QC of gp8-D99K/D101K-F	TGAGCAAACCCAAAGGACTCGCTAAGGTC GATG
pYZ10 88	QC of gp8-D99K/D101K-R	AGTCCTTTGGGTTTGCTCAGTAACTGCTTT GCTTC
pYZ10 89	QC of gp8-D220R/E221R/D222R/E225R-F	AGACGTAGGTCAGGTAGGTACCTCCGATA CGAAGAGGT
pYZ10 90	QC of gp8-D220R/E221R/D222R/E225R-R	CCTACCTGACCTACGTCTCAGATAGATGT GAGTGTACACGTC
pYZ10 91	QC of gp8-D220R/E221R-F	ATCTGCGACGTGACTCAGGTGAATACCTC CG

pYZ10 92	QC of gp8-D220R/E221R-R	GAGTCACGTCGCAGATAGATGTGAGTGTA CACGTC
pYZ10 93	QC of gp8-D222R/E225R-F	AGCGTTCAGGTAGGTACCTCCGATACGAA GAGGT
pYZ10 94	QC of gp8-D222R/E225R-R	TACCTACCTGAACGCTCATCCAGATAGAT GTGAGTGTA
pYZ10 95	QC of gp8-D241K-F	AGGCTCCAAAGGGACTTATCCTAAAGAGG CTT
pYZ10 96	QC of gp8-D241K-R	AAGTCCCTTTGGAGCCTTGGACTTCCA
pYZ10 97	QC of gp8-D457D460KK-F	GGAAAGACCCTAAGATTAACCTTGCGATG ATTAAGTTAC
pYZ10 98	QC of gp8-D457D460KK-R	ATCTTAGGGTCTTTCCGCATAGGTGCCAG T
pYZ10 99	QC of gp8-D502R-F	GTATGAGGAATGGTGCTGCTGCG
pYZ11 00	QC of gp8-D502R-R	CACCATTCCTCATACCCATTTGCATAGACT GT
pYZ11 01	QC of gp8-E10KD11K-F	TTGCGAAGAAAGGCGCAAAGTCTGTCTAT G
pYZ11 02	QC of gp8-E10KD11K-R	GCGCCTTTCTTCGCAAGTCCTGTTCGTTT
pYZ11 03	QC of gp8-E149K/E151K-F	ACCGAAGCCGAAGGGGTCAAACTATAAT CCCATGA
pYZ11 04	QC of gp8-E149K/E151K-R	ACCCCTTCGGCTTCGGTAGGTACAGCAGG AC
pYZ11 05	QC of gp8-E192R/D193K-F	CTAGAAAGATCCGTAAGGCTGTAGAAGGT
pYZ11 06	QC of gp8-E192R/D193K-R	TACGGATCTTTCTAGGGAGAGCACCAAAA GC
pYZ11 07	QC of gp8-E199R/E204R-F	AGGGGTCAAGGTGGTAGAAAGAAAGCTG ATGAGACAATCG
pYZ11 08	QC of gp8-E199R/E204R-R	TCTACCACCTTGACCCCTTACAGCCTTACG GATGTCCTC
pYZ11 09	QC of gp8-E236R-F	GGGTATGAGAGTCCAAGGCTCCGATG
pYZ11 10	QC of gp8-E236R-R	TTGGACTCTCATACCCTCGACCTCTTCG
pYZ11 11	QC of gp8-E247R-F	TCCTAAAAGGGCTTGCCCATACATCC

pYZ11 12	QC of gp8-E247R-R	GCAAGCCCTTTTAGGATAAGTCCCATCGG
pYZ11 13	QC of gp8-E280R/E284R-F	TCGCAATCTCCAACGCGCTATCGTCAAGA TGTCCAT
pYZ11 14	QC of gp8-E280R/E284R-R	CGCGTTGGAGATTGCGAAGGGACCGTAA GTCACCTA
pYZ11 15	QC of gp8-E416KE423K-F	AAGTTACCTAAGGAAGCCGTAAAGCCAA CCATTAGTACAGGTCTG
pYZ11 16	QC of gp8-E416KE423K-R	TTACGGCTTCCTTAGGTAACTTAGGAATC TGTTGCGTGGC
pYZ11 17	QC of gp8-E485E486KK-F	TCACCAAGAAGCAGAAGCAACAGAAGAT GGC
pYZ11 18	QC of gp8-E485E486KK-R	TTCTGCTTCTTGGTGAGTAGAATACCAGA AGTGTCA
pYZ11 19	QC of gp8-E521R/D528R-F	CGAGCTATGGCTGCTGCCGCTCGATCCGT AGGTTTACAGCCG
pYZ11 20	QC of gp8-E521R/D528R-R	TCGAGCGGCAGCAGCCATAGCTCGAGGTG AAGCTGTAGCTTGTG
pYZ11 21	QC of gp8-K4D/R5D-F	CTGAGGACGACACAGGACTTGCGGAGGA
pYZ11 22	QC of gp8-K4D/R5D-R	CCTGTGTCGTCCTCAGCCATGGGTACCC
pYZ11 23	QC of gp8-K94D-F	TGAAGCAGACCAGTTACTGAGCGACCCC
pYZ11 24	QC of gp8-K94D-R	AACTGGTCTGCTTCATATTCAGATATAGT AAGTCG
pYZ11 25	QC of gp8-K105E-F	ACTCGCTGAGGTCGATGAGGGCCT
pYZ11 26	QC of gp8-K105E-R	TCGACCTCAGCGAGTCCATCGGG
pYZ11 27	QC of gp8-K196E-F	CATCCGTGAGGCTGTAGAAGGTCAAGGTG
pYZ11 28	QC of gp8-K196E-R	ACAGCCTCACGGATGTCCTCAGGGAG
pYZ11 31	QC of gp8-K288D/K295D-F	GATATGTCCATGATTAGCTCTGATGTTATC GGCTTAGTGAATCCTG
pYZ11 32	QC of gp8-K288D/K295D-R	ATCAGAGCTAATCATGGACATATCGACGA TAGCCTCTTGGAGATT
pYZ11 35	QC of gp8-R277D-F	GTGACTTAGACTCCCTTGAAAATCTCCAA G

pYZ1136	QC of gp8-R277D-R	AGGGAGTCTAAGTCACCTAAGTATTCCTCAATGTA
pYZ1318	iVEC-N4-gp59-F2	gaattcAAGAAGGAGATATACCatgGAACAAAACACTGATTCTATGGTTC
pYZ1319	iVEC-N4-gp59-R2	TCTCATCCGCCAAAACAGCCAAGCTTAATTACCTAATCTAATTCCTGGGT
pYZ1320	iVEC-P1-portalp-F	gaattcAAGAAGGAGATATACCatgGCAGACAATAAAATCACGCTATC
pYZ1321	iVEC-P1-portalp-R	CTCATCCGCCAAAACAGCCAAGCTTAGTCATTATCGTTTCCCTCTTT
pYZ1285	iVEC-P1-portalp-F	GTCGACTCTAGAGGATCCCCGGGTAGCAGACAATAAAATCACGCTATC
pYZ1286	iVEC-P1-portalp-R	GTTGtaaaacgacggccAGTgaatTCAGTCATTATCGTTTCCCTC
pYZ1287	iVEC-N4-gp59-F	GTCGACTCTAGAGGATCCCCGGGTAGAACAAAACACTGATTCTATGGTTC
pYZ1288	iVEC-N4-gp59-R	GTTGtaaaacgacggccAGTgaaTTAATTACCTAATCTAATTCCTGGGT
pYZ1293	N4gp59-seqF	GTTCCTTTAGCAAACCACC
pYZ1294	P1portalP-seqF	GGCCTAAGTCAAACCTTATGC

Supplementary References

1 Zhang, Y., Zborníková, E., Rejman, D. & Gerdes, K. Novel (p)ppGpp Binding and Metabolizing Proteins of Escherichia coli. *mBio* **9** (2018). https://doi.org/10.1128/mBio.02188-17

2 Kitagawa, M. *et al.* Complete set of ORF clones of Escherichia coli ASKA library (A Complete Set of E. coli K-12 ORF Archive): Unique Resources for Biological Research. *DNA Research* **12**, 291-299 (2006). https://doi.org/10.1093/dnares/dsi012

3 Grucela, P. K. & Zhang, Y. E. Basal level of ppGpp coordinates Escherichia coli cell heterogeneity and ampicillin resistance and persistence. *Microb Cell* **10**, 248-260 (2023). https://doi.org/10.15698/mic2023.11.808

4 Liu, Y. *et al.* Basal ppGpp regulation by SpoT coordinates metabolic homeostasis and acid resistance. *bioRxiv*, 2026.2001.2026.700336 (2026). https://doi.org/10.64898/2026.01.26.700336

5 Gray Michael, J. Inorganic Polyphosphate Accumulation in Escherichia coli Is Regulated by DksA but Not by (p)ppGpp. *Journal of Bacteriology* **201**, 10.1128/jb.00664-00618 (2019). https://doi.org/10.1128/jb.00664-18

6 Gray Michael, J. Interactions between DksA and Stress-Responsive Alternative Sigma Factors Control Inorganic Polyphosphate Accumulation in Escherichia coli. *Journal of Bacteriology* **202**, 10.1128/jb.00133-00120 (2020). https://doi.org/10.1128/jb.00133-20

7 Baba, T. *et al.* Construction of Escherichia coli K-12 in-frame, single-gene knockout mutants: the Keio collection. *Mol Syst Biol* **2**, 2006.0008 (2006). https://doi.org/10.1038/msb4100050
