# Supplementary material for: Phage portal proteins counteract stringent-response–mediated restriction
Source: Nat Commun. 2026 Jul 11;17:8557. doi: 10.1038/s41467-026-75532-5 (PMC13483052; doi:10.1038/s41467-026-75532-5)
Supplement: Supplementary file 3 [file 41467_2026_75532_MOESM3_ESM.pdf]

nature portfolio

Peer Review File

Phage portal proteins counteract stringent-response–mediated restriction

Corresponding Author: Dr Yong Zhang

This file contains all reviewer reports in order by version, followed by all author rebuttals in order by version.

Version 0:

Reviewer comments:

Reviewer #1

(Remarks to the Author)
The manuscript by Kronborg, Wang, et al. presents a nice set of data to demonstrate how phage T7 portal protein Gp8 binds and inhibits the ppGpp synthesis enzymes RelA and SpoT in Escherichia coli to facilitate phage infection. This is significant as the first proof of phage proteins directly engaging a central bacterial stress response pathway as a counterdefense mechanism.

The data appears to be interpreted properly with different in vivo and in vitro experiments supporting the main conclusions in agreement. The methods section thoroughly describes the practical work and the statistical analyses are appropriate for the types of data.
• My only question regarding direct data interpretation is on lines 254-255 and Fig. 6c.
Were these two hybrid plates quantified? On a computer screen the spoT binding cultures do not seem to show any reasonable colouring and I would question the claim that both RelA and SpoT interact with the portal proteins here.
Also, can the authors offer any explanations for the variability in two hybrid results, considering Fig. 3a shows the T7 gp8 interacting more strongly with SpoT than RelA, then Fig 4b showing less signal for SpoT than RelA and then here in Fig. 6c the T7 protein shows practically no interaction with SpoT? Why was MacConkey agar used in the last case instead of X-gal (and not mentioned in the methods section)?
• Figs 4 & 5, please specify the meaning of error bars and shaded areas (presumably s.d. as in previous figures?)

My main suggestion is for the authors to critically review the breadth of implications that are drawn from the presented data. As it stands, the picture painted is one of the stringent response being a near-universal bacterial defense mechanism that phages specifically and conditionally repress to benefit their infection cycle.
• L273 and the following model of biphasic ppGpp modulation. While it sounds plausible, there is no experimental support that the phages actually benefit from the initial activation of the stringent response, as all presented infection data show the ΔrelAΔspoT strain as being infected more efficiently by T7 than the wild type E. coli. Therefore, I would be cautious with the following discussion regarding infection phase transitions and active stress response repurposing during the phage replication cycle (L291-295).
• Secondly, the manuscript currently does not address the possibility of some phages requiring the stringent response for efficient infection. The authors themselves show that from the Basel collection, phages T2, Bas02, and possibly Bas26 infect the ΔrelAΔspoT strain less efficiently than the wild type bacteria. Also, Lewańczyk et al. (https://doi.org/10.1093/femsml/uqaf048) recently showed that in Pseudomonas putida, across a variety of phages, around 50% of them are less infectious in a ppGpp0 host.

The manuscript is generally well-written and remarkably well proofread, with extremely few typographical errors. A few questions/suggestions for even higher clarity:
• L104-105 – What is meant here by “Across genetic backgrounds”? The wording makes the results seem more broad than the strains presented in Fig.1, which, I would argue, mostly constitute the same ΔrelA background with different spoT alleles (+ the wild type).
• L126-127 “Expression of four T7 proteins, Gp1.5, Gp4.2, Gp6.5, and Gp8, showed reproducible suppression of toxicity“ – consider rephrasing for clarity, in this case expression of T7 proteins does not suppress toxicity, rather the existence of a mutant rpoB allele does.
Minor notes:

• L217, 223 – References should be to Fig. S5a and b, not S7a and b
• L254 – Reference should be to Fig. S6, not S8
• L789 – Please correct spelling of Laemmli.

Reviewer #2

(Remarks to the Author)
In this manuscript, the authors sought to identify phage proteins that antagonize the stringent response (SR) in the bacterial host to ensure rapid phage particle replication and lytic cycling. They screened T7 genes for the ability to specifically block E. coli growth in minimal-nutrient medium, which requires an active SR. Through this screen, they identified the portal protein Gp8 as an inhibitor of the synthesis of the SR alarmone (p)ppGpp by RelA and SpoT. Using bacterial two-hybrid and in vitro pulldown assays, they showed that several charged residues of Gp8 are essential for binding to RelA and SpoT, allowing the authors to generate Gp8 mutants that selectively ablate its inhibition of the stringent response. T7 phage encoding such mutant Gp8 caused significant (p)ppGpp accumulation in host E. coli and slower lysis. The authors further examined the portal proteins of coliphages P1 and N4 and claimed that the SR-antagonizing function is conserved in these homologs. The implications of this finding for phage–host interactions were discussed thoroughly and elegantly.

As the “emergency brake” of bacterial physiology, SR promotes survival by preventing excessive macromolecule synthesis that could otherwise deplete essential metabolites and disrupt metabolic regulation. How bacteriophages override this intrinsic mechanism to maximize their exploitation of host resources has been a long-standing mystery and is of general interest in the fields of bacteriology and evolutionary biology. Thus, I feel this manuscript warrants publication in Nature Communications after a few technical issues are addressed.

My main concern relates to the conclusion that Gp8 inhibits RelA and SpoT by direct interactions.

1. Does RelA or SpoT bind the Gp8 monomer or the homododecamer? In particular, if Gp8 forms an obligate dodecamer, how can Gp8 achieve saturation of SpoT inhibition at a 2:1 molar ratio in Fig. 3c (I assume Gp8 is counted as monomers)?

2. Also related to Fig. 3c, RelA is predominantly activated through interactions with uncharged tRNA and the ribosome. How relevant is the in vitro synthetase activity of full-length, purified RelA to its mechanism of action in vivo?

3. While Fig. 4d suggests that Gp8 targets the NTD (HD–SYNTH domains) of SpoT and the CTD (TGS–RRM–ACT domains) of RelA, the effects of all Gp8 mutations in Fig. 4c on SpoT and RelA binding are strikingly similar. Because the SpoT NTD and RelA CTD are very unlikely to present a similar recognition interface for Gp8, I suggest that the authors consider two alternative possibilities:

(A) The mutations may affect the folding or the monomer–dodecamer equilibrium of Gp8. It would therefore be important to test whether recombinant WT and D220R/E221R Flag-Gp8 exhibit comparable solution behavior at their cellular concentrations and concentrations used for pull-down assay, and whether the mutant remains inhibitory in the in vitro synthetase assay shown in Fig. 3c.

(B) Bacterial two-hybrid assays can detect indirect interactions bridged by other factors. Gp8 may bind a shared interaction partner of RelA and SpoT (potentially the ribosome), thereby producing the positive two-hybrid result. Such an interaction could also abolish activation of both synthetases by their cognate ribosome-associated states. The authors may consider performing a two-hybrid experiment between Gp8 and a ribosomal protein to help rule out ribosome-mediated indirect interaction.

Minor points
Line 61 — Please define RSH at first use.
Line 287 — (p)ppGpp accumulation depletes GTP, but not ATP.
Fig. 6b, P1 panel — The image is difficult to interpret clearly, especially in print. The plaque-size difference is not obvious. Could the authors increase plaque size by using longer incubation time?
Fig. 6c — The two-hybrid assays involving SpoT, including the assay between T7 Gp8 and SpoT, do not look convincingly positive. Could the authors repeat these experiments using the same assay format as in Fig. 3a?

Version 1:

Reviewer comments:

Reviewer #1

(Remarks to the Author)
The revised manuscript adequately addresses my concerns regarding the initial submitted version and I am happy to recommend it for publication in Nature Communications.

Reviewer #2

(Remarks to the Author)
The authors have satisfactorily addressed my concerns except the major concern 3 of the first review--

I agree that the D220R/E221R is unlikely to affect dodecamer formation of Gp8. According to the electrostatic surface map in Fig. 4a, however, mutations like E280R/E284R and K288D/K294D almost leads to crowding of positive and negative charges in the stem/clip region and may cause electrostatic repulsion to destabilize Gp8 dodecamers.

In lines 174-177 the authors state that--
"Several mutations - including D220R/E221R, D457K/D460K, E280R/E284R, K4D/R5D, and K288D/K295D - abolished interaction entirely, identifying discrete charged surface patches (Fig. 4a) that are jointly required for targeting both enzymes.
"

which I think should be revised slightly to consider the possibility that some of these surface patches may be instead required for Gp8 dodecamer stability.

Open Access This Peer Review File is licensed under a Creative Commons Attribution 4.0 International License, which permits use, sharing, adaptation, distribution and reproduction in any medium or format, as long as you give appropriate credit to the original author(s) and the source, provide a link to the Creative Commons license, and indicate if changes were made.
In cases where reviewers are anonymous, credit should be given to 'Anonymous Referee' and the source.
The images or other third party material in this Peer Review File are included in the article's Creative Commons license, unless indicated otherwise in a credit line to the material. If material is not included in the article's Creative Commons license and your intended use is not permitted by statutory regulation or exceeds the permitted use, you will need to obtain permission directly from the copyright holder.
To view a copy of this license, visit https://creativecommons.org/licenses/by/4.0/

Rebuttal

Reviewer #1 (Remarks to the Author):

The manuscript by Kronborg, Wang, et al. presents a nice set of data to demonstrate how phage T7 portal protein Gp8 binds and inhibits the ppGpp synthesis enzymes RelA and SpoT in Escherichia coli to facilitate phage infection. This is significant as the first proof of phage proteins directly engaging a central bacterial stress response pathway as a counterdefense mechanism.

The data appears to be interpreted properly with different in vivo and in vitro experiments supporting the main conclusions in agreement. The methods section thoroughly describes the practical work and the statistical analyses are appropriate for the types of data.
• My only question regarding direct data interpretation is on lines 254-255 and Fig. 6c. Were these two hybrid plates quantified? On a computer screen the spoT binding cultures do not seem to show any reasonable colouring and I would question the claim that both RelA and SpoT interact with the portal proteins here.
Also, can the authors offer any explanations for the variability in two hybrid results, considering Fig. 3a shows the T7 gp8 interacting more strongly with SpoT than RelA, then Fig 4b showing less signal for SpoT than RelA and then here in Fig. 6c the T7 protein shows practically no interaction with SpoT? Why was MacConkey agar used in the last case instead of X-gal (and not mentioned in the methods section)?

Response:

We thank the reviewer for this careful observation. We agree that the original Fig. 6c, which used a MacConkey-based readout, did not allow sufficiently clear evaluation of the SpoT interaction signal and was not ideal for comparison with the other bacterial two-hybrid assays. We have therefore replaced the original Fig. 6c with the corresponding LB/X-gal bacterial two-hybrid assay, newly performed in the same format as the other B2H assays in the manuscript, and have added quantification of the interaction signals. The original MacConkey plate has been moved to Supplementary Fig. 6c as a qualitative supporting image, and the Methods section has been amended to describe this assay format (L553-557).

The revised and quantified X-gal data show the same overall trend: portal proteins from both P1 and N4 interact more strongly with RelA than with SpoT under these assay

conditions, while SpoT interaction signals remain detectable. We have revised the Results text accordingly to state this more precisely (L249-253).

Regarding the apparent variability among Figs. 3a, 4b and new 6c, we agree that absolute color intensity should not be compared directly across different bacterial two-hybrid experiments, especially when different constructs, expression level, incubation times, plate-to-plate variation, and image acquisition are known to affect the readout. We are aware of these factors and therefore interpret these assays only as *qualitative* or semi-quantitative interaction readouts within each experiment, rather than as directly comparable quantitative measurements across different panels and experiments. Importantly, our conclusion does not rely on bacterial two-hybrid data alone, but is supported by the concordance of repeated two-hybrid assays, pull-down experiments and in vitro inhibition assays. In response to the reviewer's comment, we have also globally toned down statements that previously implied quantitative comparison of interaction strength across independent B2H experiments.

• Figs 4 & 5, please specify the meaning of error bars and shaded areas (presumably s.d. as in previous figures?)

We thank the reviewer for noting this. The legends for all Figures now explicitly define the error bars as mean ± s.d. The shaded areas in the lysis curves likewise indicate ± s.d. around the mean.

My main suggestion is for the authors to critically review the breadth of implications that are drawn from the presented data. As it stands, the picture painted is one of the stringent response being a near-universal bacterial defense mechanism that phages specifically and conditionally repress to benefit their infection cycle.
• L273 and the following model of biphasic ppGpp modulation. While it sounds plausible, there is no experimental support that the phages actually benefit from the initial activation of the stringent response, as all presented infection data show the ΔrelAΔspoT strain as being infected more efficiently by T7 than the wild type E. coli. Therefore, I would be cautious with the following discussion regarding infection phase transitions and active stress response repurposing during the phage replication cycle (L291-295).

Response:

We thank the reviewer for this thoughtful and constructive comment. We agree that the specific fitness benefit of early (p)ppGpp accumulation during T7 infection is not directly isolated or functionally demonstrated in the present study. At the same time, we do not view the biphasic scenario as completely unsupported speculation. Rather, it is a biologically motivated interpretation based on both prior work and our current data. Previous studies have shown that efficient shutdown of host RNAP activity is important for productive T7 infection (Qimron et al, 2008), while ppGpp preferentially inhibits *E. coli* RNAP and has little effect on T7 RNAP (Kingston et al, 1981; Tabib-Salazar et al, 2022). In addition, early alarmone accumulation during infection has been reported previously (Tabib-Salazar et al, 2018) and is also consistent with our observations (supplementary Fig 5b). Taken together, these considerations make it reasonable to propose that early SR activation may transiently favor host takeover, whereas continued alarmone accumulation would become detrimental at later stages, when phage replication, structural protein synthesis, and genome packaging impose high biosynthetic demands.

We nevertheless agree with the reviewer that this early beneficial role is not directly proven in the present work. We have therefore toned down and revised (L270-273, L289-298) the manuscript to present the biphasic scenario explicitly as a “working model” that is supported by prior literature and consistent with our data, rather than as an experimentally established requirement. In the revised Fig. 7 legend (L1013-1021), we have also toned down wording accordingly.

• Secondly, the manuscript currently does not address the possibility of some phages requiring the stringent response for efficient infection. The authors themselves show that from the Basel collection, phages T2, Bas02, and possibly Bas26 infect the ΔrelAΔspoT strain less efficiently than the wild type bacteria. Also, Lewańczyk et al. (https://doi.org/10.1093/femsml/uqaf048) recently showed that in Pseudomonas putida, across a variety of phages, around 50% of them are less infectious in a ppGpp0 host.

Response:
We thank the reviewer for this important comment and fully agree that the relationship between phage infection and the stringent response is unlikely to be uniform across all phages. We also agree that our original wording could have given the impression that portal-mediated suppression of the stringent response represents a broadly conserved or near-universal phage strategy, which was not our intention. We have therefore revised the manuscript throughout to avoid overly broad generalizations and to frame our findings more cautiously.

In the revised manuscript (L227, L289-298), we now state that the mechanism described here is supported for T7 and the representative coliphages examined in this study, and that the broader role of (p)ppGpp during phage infection is likely to be phage-specific, host-specific, and temporally regulated. This revised framing also accommodates the reviewer's point that a subset of phages may benefit from, or even require, the stringent response for efficient infection. Indeed, although a majority of the phages tested in our Basel collection panel (L235) showed a T7-like pattern, phages such as T2, Bas02 and Bas26 infected the ΔrelAΔspoT strain less efficiently than wild-type bacteria. The recent findings of Lewańczyk et al. in *Pseudomonas putida* (now cited as ref 46, L293) further support the idea that (p)ppGpp can be beneficial for infection in some phage–host contexts.

We now discuss these observations in the context of our biphasic model (L289-298). In this model, early alarmone accumulation may in some cases facilitate host takeover, for example by promoting host transcriptional repression or metabolic reprogramming, whereas sustained or late-stage (p)ppGpp accumulation may become detrimental by limiting biosynthesis, nucleotide availability, genome packaging and virion production. Thus, our intention is not to argue that all phages antagonize the stringent response, but rather that portal-mediated targeting of stringent-response enzymes represents one demonstrable strategy by which phages can modulate alarmone levels during infection. We have also revised the whole Abstract and introduction (L79, L99-101) accordingly to make this more nuanced interpretation explicit.

The manuscript is generally well-written and remarkably well proofread, with extremely few typographical errors. A few questions/suggestions for even higher clarity:

• L104-105 – What is meant here by "Across genetic backgrounds"? The wording makes the results seem more broad than the strains presented in Fig.1, which, I would argue, mostly constitute the same ΔrelA background with different spoT alleles (+ the wild type).

Response:
We thank the reviewer for this suggestion and agree that the original wording was broader than warranted by the strains shown in Fig. 1. We have revised this sentence (L 95-97) to specify the scope of the comparison more precisely.

• L126-127 "Expression of four T7 proteins, Gp1.5, Gp4.2, Gp6.5, and Gp8, showed reproducible suppression of toxicity" – consider rephrasing for clarity, in this case expression of T7 proteins does not suppress toxicity, rather the existence of a mutant

rpoB allele does.
Response:
We agree that the original wording could be misread. We have revised the sentence (L118-120) to make clear that the suppression is conferred by the $RNAP^{SR}$ background rather than by expression of the T7 proteins themselves.

Minor notes:
• L217, 223 – References should be to Fig. S5a and b, not S7a and b
Response: The references have been corrected.

• L254 – Reference should be to Fig. S6, not S8
Response: The references have been corrected.

• L789 – Please correct spelling of Laemmli.
Response: corrected

Reviewer #2 (Remarks to the Author):

In this manuscript, the authors sought to identify phage proteins that antagonize the stringent response (SR) in the bacterial host to ensure rapid phage particle replication and lytic cycling. They screened T7 genes for the ability to specifically block E. coli growth in minimal-nutrient medium, which requires an active SR. Through this screen, they identified the portal protein Gp8 as an inhibitor of the synthesis of the SR alarmone (p)ppGpp by RelA and SpoT. Using bacterial two-hybrid and in vitro pulldown assays, they showed that several charged residues of Gp8 are essential for binding to RelA and SpoT, allowing the authors to generate Gp8 mutants that selectively ablate its inhibition of the stringent response. T7 phage encoding such mutant Gp8 caused significant (p)ppGpp accumulation in host E. coli and slower lysis. The authors further examined the portal proteins of coliphages P1 and N4 and claimed that the SR-antagonizing function is conserved in these homologs. The implications of this finding for phage–host interactions were discussed thoroughly and elegantly.

As the “emergency brake” of bacterial physiology, SR promotes survival by preventing excessive macromolecule synthesis that could otherwise deplete essential metabolites and disrupt metabolic regulation. How bacteriophages override this intrinsic mechanism to maximize their exploitation of host resources has been a long-standing mystery and is of general interest in the fields of bacteriology and evolutionary biology. Thus, I feel this manuscript warrants publication in Nature Communications after a few technical issues are addressed.

My main concern relates to the conclusion that Gp8 inhibits RelA and SpoT by direct interactions.

1. Does RelA or SpoT bind the Gp8 monomer or the homododecamer? In particular, if Gp8 forms an obligate dodecamer, how can Gp8 achieve saturation of SpoT inhibition at a 2:1 molar ratio in Fig. 3c (I assume Gp8 is counted as monomers)?

Response:
We thank the reviewer for raising this important point. Structural studies of bacteriophage T7 show that Gp8 forms a dodecameric portal in the virion. To better show the purified Gp8 used in our biochemical assays, we have now included a size-exclusion chromatography profile of wild-type Gp8 as new Supplementary Fig. 3a. Purified Gp8 eluted as a single major peak earlier than the largest molecular-mass standard used, 200 kDa, indicating a relatively uniform higher-order species with an

apparent molecular mass > 200 kDa. Given the approximately 60-kDa size of the Gp8 monomer, this profile is consistent with Gp8 existing as an oligomeric assembly under the biochemical conditions used here, although SEC alone does not define the precise oligomeric state.

We have also clarified in the Fig. 3c legend that the molar ratios are expressed relative to RelA or SpoT using Gp8 monomer equivalents (L943-947). Thus, the 2:1 Gp8:SpoT ratio refers to monomer-equivalent concentrations and should not be interpreted as a defined 2:1 binding stoichiometry between assembled Gp8 particles and SpoT. Rather, the purpose of this experiment was to test whether purified Gp8 inhibits RelA and SpoT in a dose-dependent manner. If the inhibitory species is the assembled portal, its multivalent surface could in principle engage more than one target enzyme; however, our current data do not resolve the stoichiometry of that interaction.

To further aid interpretation, we have now added the IC50 values for inhibition of RelA and SpoT (Fig 3c, and L947). Together with the in vivo infection phenotypes and alarmone measurements, these biochemical data support the physiological relevance of Gp8-mediated inhibition while leaving the precise binding stoichiometry and architecture of the Gp8–RelA/SpoT complexes to future structural and mechanistic work (L340-342).

2. Also related to Fig. 3c, RelA is predominantly activated through interactions with uncharged tRNA and the ribosome. How relevant is the in vitro synthetase activity of full-length, purified RelA to its mechanism of action in vivo?

Response:
We thank the reviewer for this important point. We agree that physiological activation of *E. coli* RelA is classically associated with starved ribosomes bearing deacylated tRNA, and that our purified-protein assay does not reconstitute this full cellular activation context. We have therefore clarified this point in the revised manuscript (L143-147). Our intention with the assay in Fig. 3c is not to claim complete reconstitution of the in vivo mechanism of RelA activation, but rather to test whether Gp8 can suppress the synthetase activity of RelA in a simplified biochemical setting, analogous to its effect on SpoT. In this respect, the assay demonstrates that purified Gp8 is sufficient to inhibit purified RelA and SpoT in the absence of additional cellular components.

Importantly, this biochemical result is consistent with the in vivo data. During infection, the interaction-defective gp8 mutant fails to suppress alarmone accumulation (Fig. 5e) and shows delayed lysis relative to wild-type T7 (supplementary Fig. 5a), and these defects are alleviated in SR-deficient hosts (Fig. 5d). We therefore interpret the biochemical assay as evidence for a direct inhibitory capacity of Gp8, while

acknowledging that the precise physiological context of RelA targeting in vivo remains to be defined more fully.

3. While Fig. 4d suggests that Gp8 targets the NTD (HD–SYNTH domains) of SpoT and the CTD (TGS–RRM–ACT domains) of RelA, the effects of all Gp8 mutations in Fig. 4c on SpoT and RelA binding are strikingly similar. Because the SpoT NTD and RelA CTD are very unlikely to present a similar recognition interface for Gp8, I suggest that the authors consider two alternative possibilities:
(A) The mutations may affect the folding or the monomer–dodecamer equilibrium of Gp8. It would therefore be important to test whether recombinant WT and D220R/E221R Flag-Gp8 exhibit comparable solution behavior at their cellular concentrations and concentrations used for pull-down assay, and whether the mutant remains inhibitory in the in vitro synthetase assay shown in Fig. 3c.

Response:

We thank the reviewer for raising this important alternative explanation. We agree that, in principle, the gp8 mutations could affect portal properties beyond the RelA/SpoT interaction itself. The recovery of viable mutant phage particles, however, argues against a gross defect in portal assembly, and the substituted residues are surface-exposed in the structural model, consistent with a role in intermolecular recognition. However, we cannot fully exclude more subtle portal-associated effects.

We have therefore moderated the wording in the revised manuscript accordingly (L137-139). Importantly, the D220R/E221R mutant loses detectable interaction with RelA and SpoT in both bacterial two-hybrid and pull-down assays (Fig 4c, 5a), fails to suppress alarmone accumulation efficiently during infection (Fig 5e), and its infection defect is alleviated in SR-deficient hosts (Fig 5d). Taken together, these findings consistently support a specific contribution of RelA/SpoT targeting to the observed phenotype, even if additional portal-associated effects cannot be completely excluded.

(B) Bacterial two-hybrid assays can detect indirect interactions bridged by other factors. Gp8 may bind a shared interaction partner of RelA and SpoT (potentially the ribosome), thereby producing the positive two-hybrid result. Such an interaction could also abolish activation of both synthetases by their cognate ribosome-associated states. The authors may consider performing a two-hybrid experiment between Gp8 and a ribosomal protein

to help rule out ribosome-mediated indirect interaction.

Response:
We thank the reviewer for this thoughtful suggestion. We agree that bacterial two-hybrid assays alone cannot distinguish direct interactions from interactions bridged by other cellular factors. We have carefully checked the wording in the manuscript to present the bacterial two-hybrid data more cautiously as an in vivo interaction readout rather than as sole evidence for direct binding.

Importantly, our purified-component biochemical assays show that Gp8 inhibits the synthetase activities of purified RelA and SpoT in vitro, indicating that this inhibitory effect does not require ribosomes or other cellular proteins. We therefore think that the key concern — whether ribosome-associated factors are required for Gp8-mediated inhibition — is addressed by these biochemical data.

We also carefully considered the suggested ribosomal-protein two-hybrid experiment. However, a comprehensive robust test would require constructing at least 60–70 additional two-hybrid vectors and may still not provide a definitive mechanistic answer. We also explored an unbiased mutagenesis approach to identify RelA or SpoT variants that restore interaction with the interaction-defective Gp8 D220R/E221R mutant, but this did not yield informative suppressors. We therefore believe that higher-resolution approaches, such as structural analysis and detailed interface mapping, will be more informative for defining the Gp8–RelA/SpoT interaction mechanism. We have added this point to the Discussion as a limitation and future direction (L337-343).

Minor points
Line 61 — Please define RSH at first use.

Response:
RSH is now defined at first use as “RelA-SpoT homolog” (L53).

Line 287 — (p)ppGpp accumulation depletes GTP, but not ATP.

Response:
We thank the reviewer for this correction. We have corrected the texts accordingly (L219, L283).

Fig. 6b, P1 panel — The image is difficult to interpret clearly, especially in print. The plaque-size difference is not obvious. Could the authors increase plaque size by using

longer incubation time?

Response:
We thank the reviewer for this suggestion. We tested longer incubation, but the P1 plaques remained intrinsically small under our assay conditions. We have therefore improved the image presentation in the revised figure to make the phenotype easier to visualize.

Fig. 6c — The two-hybrid assays involving SpoT, including the assay between T7 Gp8 and SpoT, do not look convincingly positive. Could the authors repeat these experiments using the same assay format as in Fig. 3a?

Response:

We thank the reviewer for this helpful suggestion. We have replaced the original Fig. 6c with an LB/X-gal bacterial two-hybrid assay performed in the same format as Fig. 3a and have added quantification of the interaction signals. The previous MacConkey-based plate image has been moved to Supplementary Fig. 6c as a qualitative supporting readout, and the Methods section has been amended accordingly (L553-557).

The revised data (new Fig 6c) show detectable interaction signals between the P1 and N4 portal proteins and both RelA and SpoT, with RelA producing stronger interaction signals than SpoT under these conditions. We have revised the Results text to reflect this more precise interpretation (L249-253).

Response to remaining reviewer comment

REVIEWERS' COMMENTS

Reviewer #1 (Remarks to the Author):

The revised manuscript adequately addresses my concerns regarding the initial submitted version and I am happy to recommend it for publication in Nature Communications.

Reviewer #2 (Remarks to the Author):

The authors have satisfactorily addressed my concerns except the major concern 3 of the first review--

I agree that the D220R/E221R is unlikely to affect dodecamer formation of Gp8. According to the electrostatic surface map in Fig. 4a, however, mutations like E280R/E284R and K288D/K294D almost leads to crowding of positive and negative charges in the stem/clip region and may cause electrostatic repulsion to destabilize Gp8 dodecamers.

In lines 174-177 the authors state that--
"Several mutations - including D220R/E221R, D457K/D460K, E280R/E284R, K4D/R5D, and K288D/K295D - abolished interaction entirely, identifying discrete charged surface patches (Fig. 4a) that are jointly required for targeting both enzymes. "

which I think should be revised slightly to consider the possibility that some of these surface patches may be instead required for Gp8 dodecamer stability.

Response:

We thank the reviewer for this helpful clarification. We agree that, some charge-reversal mutations in the stem/clip region could in principle alter local electrostatics and affect portal conformation or oligomer stability. We have therefore revised the wording in the Results to avoid implying that all interaction-defective mutations necessarily define direct RelA/SpoT-binding surfaces.

The revised text now states:

"Several mutations, including D220R/E221R, D457K/D460K, E280R/E284R, K4D/R5D and K288D/K295D, abolished detectable interaction with both RelA and SpoT in the bacterial two-hybrid assay, indicating that these charged regions contribute to the Gp8-dependent interaction readout (Fig. 4a). Because some of these charge-reversal mutations lie within the stem/clip region, altered local electrostatics may also affect portal conformation or oligomer stability, potentially explaining the lack of viable mutant phage recovery described below."

This change can be found at L174–180 and L970 in the revised manuscript and has been highlighted.
